# An efficient Pd–NHC catalyst system in situ generated from Na_2_PdCl_4_ and PEG-functionalized imidazolium salts for Mizoroki–Heck reactions in water

**DOI:** 10.3762/bjoc.13.168

**Published:** 2017-08-21

**Authors:** Nan Sun, Meng Chen, Liqun Jin, Wei Zhao, Baoxiang Hu, Zhenlu Shen, Xinquan Hu

**Affiliations:** 1 College of Chemical Engineering, Zhejiang University of Technology, Hangzhou 310032, China

**Keywords:** aqueous homogeneous catalysis, Mizoroki–Heck reaction, Na_2_PdCl_4_, PEG-functionalized imidazolium salts

## Abstract

Three PEG-functionalized imidazolium salts **L1**–**L3** were designed and prepared from commercially available materials via a simple method. Their corresponding water soluble Pd–NHC catalysts, in situ generated from the imidazolium salts **L1**–**L3** and Na_2_PdCl_4_ in water, showed impressive catalytic activity for aqueous Mizoroki–Heck reactions. The kinetic study revealed that the Pd catalyst derived from the imidazolium salt **L1**, bearing a pyridine-2-methyl substituent at the N3 atom of the imidazole ring, showed the best catalytic activity. Under the optimal conditions, a wide range of substituted alkenes were achieved in good to excellent yields from various aryl bromides and alkenes with the catalyst TON of up to 10,000.

## Introduction

Nowadays, both increasing environmental concerns and drastic commercial competition are the driving forces to develop more sustainable and economic processes for important chemicals syntheses in both academic and industrial fields [1–2]. In fine chemical industries, organic solvents still dominate in modern synthetic processes since they are capable of dissolving a wide range of organic compounds and controlling the reaction selectivity and rate. However, they are often volatile, toxic, flammable and expensive as well as might introduce a bulk of hazardous waste treatment issues. Thus, great efforts have been put into reducing or eliminating those organic solvents by replacing them with more environmentally acceptable alternatives [3]. It is beyond doubt that water is a preferred choice because of its abundance, non-toxicity, non-flammability, as well as minimum environmental impacts. In addition, using water as medium often leads to exceptional chemical reactivity and selectivity owing to its unique physicochemical properties [4–6].

The palladium-catalyzed cross-coupling reactions to form C–C bonds are very powerful synthetic tools in modern organic synthesis [7]. With their increasing applications in the synthesis of pharmaceuticals, natural products and functional materials [8–10], moving these useful transformations to occurring in aqueous media became more and more attractive [11]. Despite there are several strategies for palladium-catalyzed cross-coupling reactions in water, such as microwave heating [12], ultrasonic irradiation [13–14] and ligand-free methodology [15–16], the more efficient and preferable one is the use of water-soluble ligated palladium catalysts. This approach not only enhances the water solubility of the catalyst, but also facilitates the recovery of the catalyst by separating the aqueous phase and subsequently for the potential reuse of catalyst [17]. Initially, such catalysts have been obtained through modifying traditional palladium–phosphine catalysts by grafting various hydrophilic substituents on phosphine ligands [18–27]. However, most of these phosphine ligands are air sensitive and required tedious work to preparation. In addition, the easy dissociation of common P–Pd bonds under aqueous reaction conditions often restricted the reuse of the catalyst and led to undesired residues. Therefore, in recent years, efforts have been turned to the development of water-soluble non-phosphine ligands [28–34]. In this context, *N*-heterocyclic carbenes (NHCs) have been recognized as the preferable candidates [35–36]. In contrast to common phosphine- and nitrogen-based ligands, NHCs exhibit stronger σ-donating and weaker π-accepting properties, which make the corresponding Pd–NHC complexes more air and water stable. Furthermore, the convenient functionalization of the N atom of the NHC ring allows for the possible incorporation of water soluble moieties, thus providing more opportunities for water soluble catalyst design [37–39].

Since the pioneering report of a sulfonate-functionalized NHC ligand by Shaughnessy [40], a number of water-soluble NHC ligands, functionalized with sulfonate- [41–46], carboxylate- [47–52], polyether- [53–59] and other hydrophilic groups [60–63], have been developed and used in the aqueous Pd-catalyzed cross-coupling reactions. Among them, most of them were contributed to Suzuki–Miyaura reactions and only a very few examples were reported for Mizoroki–Heck reactions [45,51,53,57]. Previous research by Rösch and other groups disclosed that introducing a hemilable donor group (such as N, O, S etc.) on the NHC rings was favorable for the palladium-catalyzed Mizoroki–Heck cross-coupling reactions [64–65]. These electron-donating groups could provide a flexible environment for the Pd center and thus favoring the complexation and the migratory insertion of an alkene. Cavell reported that a pyridine functionalized Pd–NHC complex showed outstanding catalytic activity in Mizoroki–Heck reactions with DMF as solvent [66].

**Figure 1:**
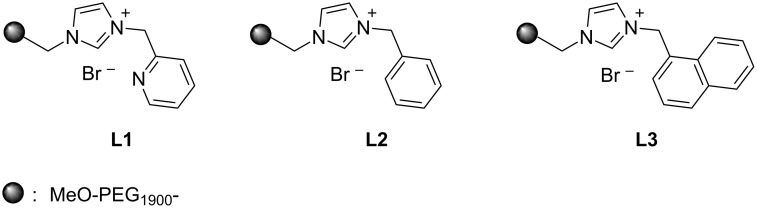
Structures of imidazolium salts **L1**–**L3**.

With this regards, we herein report the development of a new poly(ethylene glycol, PEG) and pyridine bi-functionalized imidazolium salt **L1** (Figure 1), which was employed as a water soluble NHC ligand precursor for an in situ generated Pd–NHC catalyst for Mizoroki–Heck reactions in water. Meanwhile, two analogues, phenyl (**L2**) and naphthyl (**L3**) functionalized imidazolium salts were synthesized and their catalytic activities in aqueous Mizoroki–Heck reactions were also studied.

## Results and Discussion

PEGs are a kind of highly water soluble polymers from the polymerization of ethylene oxide [67]. Owing to their significant advantages, including widely commercial availability, biocompatibility, chemical and thermal stability and ease to be derived, PEGs have been widely used as phase-transfer catalysts (PTC) or in the preparation of water soluble ligands for aqueous organic reactions during the past decades [68–69]. More recently, several PEG-functionalized azolium salts have been synthesized as water soluble NHC precursors for aqueous Pd-catalyzed cross-coupling reactions [56–59,70]. Fujihara also pointed out that the flexible linear long-chain structure of PEGs could wrap and stabilize the metal center and thus significantly enhanced the catalytic efficiency [70]. Therefore, we chose PEG as functionalization group to prepare water soluble catalysts.

**Scheme 1:**
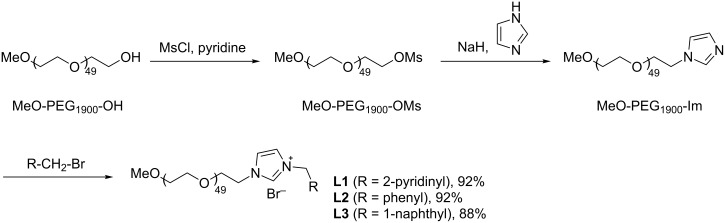
The synthetic route for the preparation of imidazolium salts **L1**–**L3**.

The PEG-functionalized imidazolium salts **L1**–**L3** were prepared via a three-step reaction sequence as depicted in Scheme 1. Firstly, the commercially available MeO-PEG_1900_-OH was reacted with MsCl using pyridine as base in CH_2_Cl_2_ to form MeO-PEG_1900_-OMs, which was then treated with sodium imidazole in THF to form the imidazole-functionalized PEG (MeO-PEG_1900_-Im). The resulted MeO-PEG_1900_-Im was heated with various organic bromides (2-(bromomethyl)pyridine, benzyl bromide and 1-(bromomethyl)naphthalene) to generate the corresponding imidazolium salts **L1**–**L3** under solvent-free conditions. All imidazolium salts were water-soluble and air-stable. The resulted salts **L1**–**L3** were characterized by ^1^H NMR, ^13^C NMR and MALDI–TOF–MS analyses (see Supporting Information File 1).

The catalytic performance of the synthesized imidazolium salts as NHC precursors for Pd-catalyzed Mizoroki–Heck reactions in water was investigated. A model reaction was carried out by using 4-bromoacetophenone (**1a**) and styrene (**2a**) as the substrates, water as solvent and Na_2_PdCl_4_/**L1** as the catalyst. The mixture of Na_2_PdCl_4_, **L1** and base in water were preheated at 60 °C for 30 min before the addition of substrates [41]. The effect of base was first explored. As the selected experimental results illustrated in Table 1, almost no reaction was observed without base at 100 °C for 12 h (entry 1, Table 1). The reaction could be obviously promoted by a wide range of common bases, such as Et_3_N, NaHCO_3_, Na_2_CO_3_, K_2_CO_3_, NaOH, NaOEt and NaO*t*-Bu. The best result was obtained with NaOEt as the base. With 2.0 equivalents of NaOEt, the desired coupling product **3aa** was achieved in 97% GC yield (entry 7, Table 1). Employing NaO*t*-Bu could also provide an excellent yield (91%, entry 8, Table 1). Weaker bases, such as Et_3_N and NaHCO_3_, led to lower yields (entries 2 and 3, Table 1). The performance of NaOEt and NaO*t*-Bu was obviously better than that of NaOH. To clarify that this improvement might be due to the generation of EtOH and *t*-BuOH from the hydrolysis of NaOEt and NaO*t*-Bu in water, we then studied the effect of EtOH and *t*-BuOH on the reaction. In contrast to the reaction in neat water with NaOH as base, the yields of **3aa** were increased from 68% to 88% and 78%, respectively, after the addition of 2.0 equivalents of EtOH and *t*-BuOH, inferring that EtOH and *t*-BuOH could facilitate the reaction. However, both of them were inferior to the reactions using NaOEt and NaO*t*-Bu as the base directly (entries 9 and 10, Table 1). Furthermore, it was found that N_2_ atmospheric conditions were crucial for the reaction and a nearly quantitative GC yield was resulted with 0.05–0.1 mol % catalyst loadings (entries 11 and 12, Table 1). Further decreasing the catalyst loading to 0.01 mol % resulted in a 89% GC yield of the coupling product **3aa** (entry 13, Table 1). Additionally, increasing the molar ratio of **L1** and Na_2_PdCl_4_ to 1.5 did not obviously affect the yield (entry 14, Table 1). However, without **L1**, the GC yield of **3aa** was dramatically decreased to 25%, which hinted that **L1** played a crucial role in this transformation (entry 15, Table 1). We also attempted to carry out the reaction at lower reaction temperature; however, much lower conversion was found (entry 16, Table 1). Moreover, a blank experiment showed that no reaction occurred without Na_2_PdCl_4_ (entry 17, Table 1). To confirm that Na_2_PdCl_4_ and **L1** in situ generated the Pd–NHC species, we treated Na_2_PdCl_4_, **L1** and NaOEt in D_2_O at 60 °C for 30 min, and then performed NMR analyses. The ^1^H NMR spectrum clearly showed that the proton signal of the 2-position (9.41 ppm) of the imidazolium salt **L1** disappeared. Two downfield signals at 180.9 and 170.9 ppm appeared in the ^13^C NMR spectrum, which is similar to the reported ^13^C NMR analysis for Pd–NHC species [66]. It is strongly suggested that a Pd–NHC complex was formed from deprotonation of **L1** under the reaction conditions. However, the exact structure of this complex is not clear yet.

**Table 1:** Optimizing the reaction conditions of the Mizoroki–Heck reaction.^a^

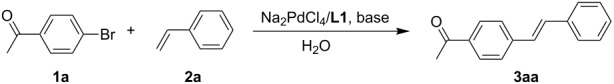			

Entry	Base	Pd:**L1** (Pd mol %)	Yield^b^ (%)

1	–	1:1 (0.1%)	trace
2	Et_3_N	1:1 (0.1%)	23
3	NaHCO_3_	1:1 (0.1%)	20
4	Na_2_CO_3_	1:1 (0.1%)	66
5	K_2_CO_3_	1:1 (0.1%)	57
6	NaOH	1:1 (0.1%)	68
7	NaOEt	1:1 (0.1%)	97
8	NaO*t*-Bu	1:1 (0.1%)	91
9	NaOH + EtOH (2.0 equiv)	1:1 (0.1%)	88
10	NaOH + *t*-BuOH (2.0 equiv)	1:1 (0.1%)	78
11^c^	NaOEt	1:1 (0.1%)	>99
12^c^	NaOEt	1:1 (0.05%)	>99
13^c^	NaOEt	1:1 (0.01%)	89
14^c^	NaOEt	1:1.5 (0.01%)	88
15^c^	NaOEt	1:0 (0.05%)	25
16^c,d^	NaOEt	1:1 (0.05%)	46
17^c,e^	NaOEt	–	n.r.

^a^Reaction conditions: 4-bromoacetophenone (**1a**, 1.0 mmol), styrene (**2a**, 1.2 mmol), base (2.0 mmol), Na_2_PdCl_4_ (0.001 mmol, 0.1% aqueous solution), **L1** (0.001 mmol, 1% aqueous solution), 1.5 mL H_2_O, 100 °C, 12 h. The mixture of **L1**, Na_2_PdCl_4_ and base in water was preheated in water at 60 °C for 30 min before adding substrates **1a** and **2a**. ^b^GC yields were determined by using the area normalization method and calculated based on **1a**. ^c^Purged with N_2_. ^d^Carried out at 90 °C. ^e^Without Na_2_PdCl_4_, **L1** (0.1 mol %).

**Figure 2:**
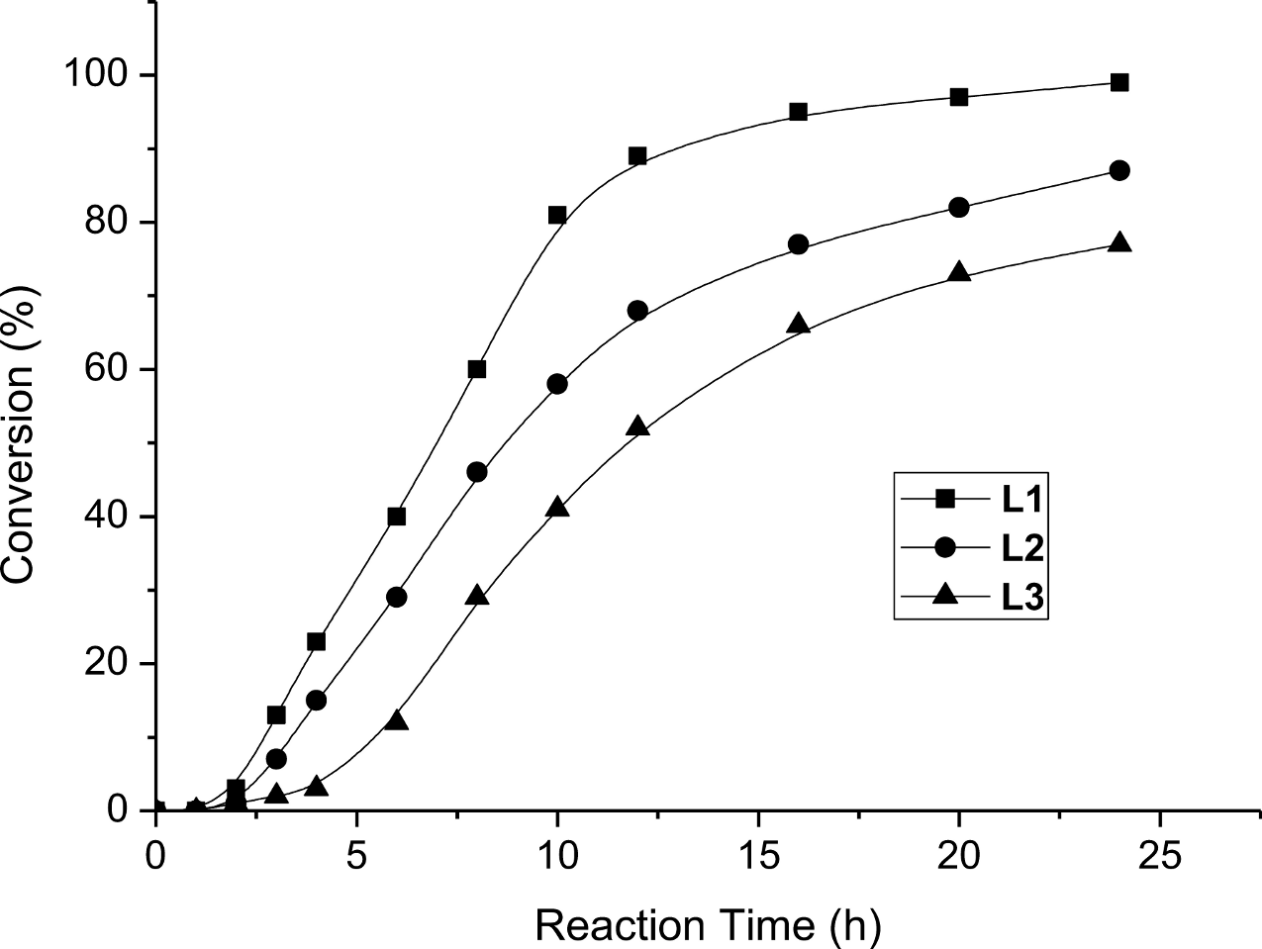
Kinetic profiles of Mizoroki–Heck reactions in water, Na_2_PdCl_4_/**L1** (square), **L2** (circle), and **L3** (triangle). Reaction conditions: 4-bromoacetophenone (**1a**, 1.0 mmol), styrene (**2a**, 1.2 mmol), NaOEt (2.0 mmol), 0.01 mol % Na_2_PdCl_4_, Pd/**L** = 1:1 (molar ratio), 1.5 mL H_2_O, 100 °C.

With the preliminary reaction conditions in hand, we then further compared the catalytic performance of those Pd-complexes derived from phenyl and naphthyl analogues **L2** and **L3** with that of pyridine functionalized NHC precursor **L1**. A kinetic study of the coupling between 4-bromoacetophenone (**1a**) and styrene (**2a**) was performed in the presence of 0.01 mol % of Na_2_PdCl_4_/**L** and 2.0 equivalents of NaOEt at 100 °C in water and all the three reactions preceded for 24 h. As shown in Figure 2, the reaction using Na_2_PdCl_4_/**L1** as the catalyst had a relatively shorter induction period and a higher catalytic activity than those of Na_2_PdCl_4_/**L2** and Na_2_PdCl_4_/**L3**. After 24 h, a 100% conversion of **1a** was observed in the Na_2_PdCl_4_/**L1** catalytic system, a conversion of 87% in Na_2_PdCl_4_/**L2** and 77% in Na_2_PdCl_4_/**L3**. This result might be attributed to the side-arm pyridine group acting as a hemilable coordination site and thus enhanced the catalytic activity of the palladium complex in Mizoroki–Heck reactions. Furthermore, the TON of the coupling of 4-bromoacetophenone (**1a**) and styrene (**2a**) with Na_2_PdCl_4_/**L1** as the catalyst was calculated to be 10,000, which is much higher than for previously reported catalytic systems under aqueous conditions.

After obtaining the optimal conditions, we then started to explore the substrate scope of the newly developed catalytic system for Mizoroki–Heck reactions in water. First, a variety of *para*-substituted phenyl bromides **1a–l** were tested to couple with styrene (**2a**) and the results were summarized in Table 2 (entries 1–12). Under the optimized reaction conditions (0.05 mol % Na_2_PdCl_4_ and **L1**, 100 °C, 2.0 equivalents of NaOEt for 12 h), the coupling reactions of aryl bromides **1a**–**c** with strongly electron-withdrawing substituents (COCH_3_, CHO and NO_2_) proceeded smoothly and the desired coupling products **3aa**–**ca** were obtained in almost quantitative yields (entries 1–3, Table 2). However, higher reaction temperature (120 °C) was necessary for the coupling of aryl bromides **1d**–**g** with moderate electron-withdrawing substituents (CF_3_, F, Cl and Br) and their coupling products **3da**–**ga** could be still obtained in good to excellent yields (87–94%, entries 4–7, Table 2). It was not surprising that substrates of aryl bromides **1h**–**j** with electron-donating substituents (H, CH_3_ and OCH_3_) showed rather difficulties for the completion of the reaction. With slightly adjusting the reaction conditions (higher reaction temperature (120 °C) and higher catalyst loading (0.1 mol %), reasonable yields of coupling products **3ha–ja** could be obtained (entries 8–10, Table 2). It should be pointed out that in the reaction of 1,4-dibromobenzene (**1g**), only mono-olefinated product **3ga** was formed and not a trace of any di-olefinated product was detected. We also found that amino and hydroxy substituted aryl bromides **1k** and **1l** exhibited high reactivity in the present aqueous catalytic systems (entries 11 and 12 vs entries 1–3, Table 2). It might be attributed to the hydrogen bonding action between amino or hydroxy groups and water and thus activated these two substrates. Then, the reactivity of *meta*- or *ortho*-substituted phenyl bromides **1m**–**r** were examined (entries 13–18, Table 2). Compared with *para*-substituted analogues **1a**, **1b** and **1i**, the *meta*-substituted phenyl bromides **1m**, **1n** and **1o** showed slightly lower reactivities under the same reaction conditions (entries 13–15 vs entries 1, 2, 9, Table 2). Nevertheless, the steric hindrance of phenyl bromides with a substituent at the *ortho*-position obviously stagnated the coupling reaction and the yields of the corresponding coupling products **1pa**, **1qa** and **1ra** were much lower than their *para*- and *meta*-substituted analogues (entries 16–18, Table 2). Besides the substituted phenyl bromides, 2-bromonaphthalene (**1s**) and some *N*-heteroaromatic bromides (3-bromopyridine (**1t**) and 3-bromoquinoline (**1u**)) could smoothly couple with **2a** to afford the corresponding coupling products **3sa**, **3ta** and **3ua** in good to excellent yields (84, 97 and 86%, respectively, entries 19–21, Table 2).

**Table 2:** Mizoroki–Heck reactions between substituted aryl bromides and styrene.^a^

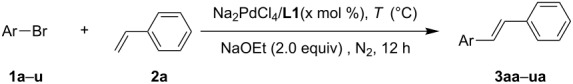					

Entry	Ar–Br **1** (R)	Product **3**	Pd/**L1** (mol %)	*T* (°C)	Yield^b^ (%)

	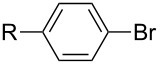	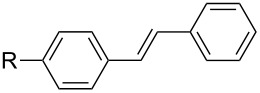			
1	**1a** (R = COCH_3_)	**3aa**	0.05	100	96
2	**1b** (R = CHO)	**3ba**	0.05	100	98
3	**1c**(R = NO_2_)	**3ca**	0.05	100	95
4	**1d** (R = CF_3_)	**3da**	0.05	120	94
5	**1e** (R = F)	**3ea**	0.05	120	87
6	**1f** (R = Cl)	**3fa**	0.05	120	90
7	**1g** (R = Br)	**3ga**	0.05	120	87
8	**1h** (R = H)	**3ha**	0.1	120	76
9	**1i** (R = CH_3_)	**3ia**	0.1	120	88
10	**1j** (R = OCH_3_)	**3ja**	0.1	120	53
11	**1k** (R = NH_2_)	**3ka**	0.05	100	87
12^c^	**1l** (R = OH)	**3la**	0.05	100	65
	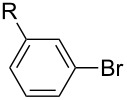	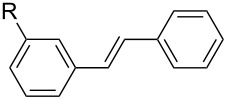			
13	**1m** (3-COCH_3_)	**3ma**	0.05	100	91
14	**1n** (3-CHO)	**3na**	0.05	100	89
15	**1o** (3-CH_3_)	**3oa**	0.1	120	77
	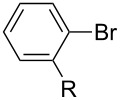	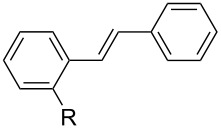			
16	**1p** (2-COCH_3_)	**3pa**	0.05	100	<10
17	**1q** (2-CHO)	**3qa**	0.05	100	51
18	**1r** (2-CH_3_)	**3ra**	0.1	120	73
19	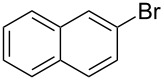 **1s**	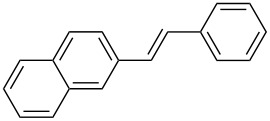 **3sa**	0.1	120	84
20	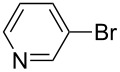 **1t**	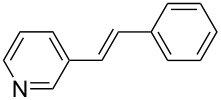 **3ta**	0.05	120	97
21	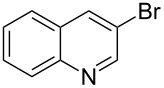 **1u**	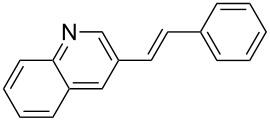 **3ua**	0.05	120	86

^a^Reaction conditions: Ar–Br **1** (1.0 mmol), styrene (**2a**, 1.2 mmol), NaOEt (2.0 mmol), Na_2_PdCl_4_ (0.05–0.1 mol %, 0.1% aqueous solution), **L1** (0.05–0.1 mol %, 1% aqueous solution), 1.5 mL H_2_O, 100 °C, 12 h, purged with N_2_. The mixture of **L1**, Na_2_PdCl_4_ and base in water was preheated in water at 60 °C for 30 min before adding substrates **1** and **2a**. ^b^Isolated yields. ^c^3.0 Equivalents of NaOEt was used.

The scope of alkenes was also investigated to couple with 4-bromoacetophenone in water (Table 3). These alkenes included *para*-substituted styrenes **2b–d** (OCH_3_, CH_3_ and Cl), 2-vinylnaphthalene (**2e**), acrylic acid (**2f**), 4-vinylpyridine (**2g**), as well as an internal alkene ((*E*)-stilbene, (**2h**)). To our delight, all these tested alkenes smoothly transformed into the corresponding products **3ab**–**ah** in excellent yields (85–97%) with 0.05–0.1 mol % of Na_2_PdCl_4_/**L1** at 100 or 120 °C (Table 3). It is noteworthy that a trace amount of 1,1-disubstituted ethylene isomers and/or *Z*-isomers in coupling products were also observed in some cases. However, the selectivity of *E*-isomers were always over 99% according to GC analyses.

**Table 3:** Mizoroki–Heck reactions between 4-bromoacetophenone and various alkenes.^a^

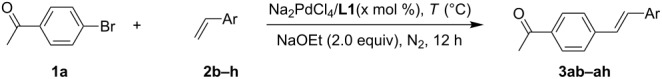					

Entry	Alkene **2**	Product **3**	Pd/**L1** (mol %)	*T* (°C)	Yield^b^ (%)

1	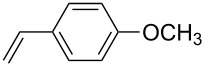 **2b**	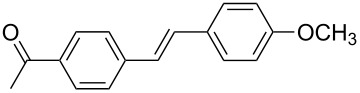 **3ab**	0.05	100	97
2	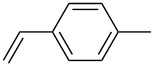 **2c**	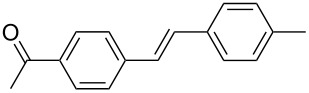 **3ac**	0.05	100	95
3	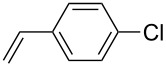 **2d**	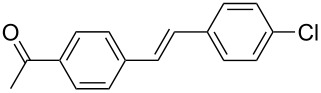 **3ad**	0.05	100	93
4	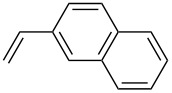 **2e**	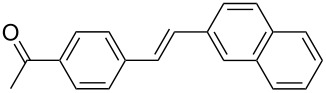 **3ae**	0.05	100	96
5^c^	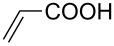 **2f**	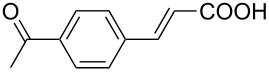 **3af**	0.1	120	89
6	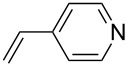 **2g**	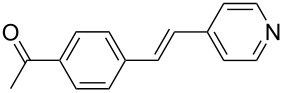 **3ag**	0.1	120	85
7	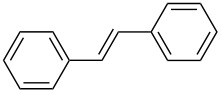 **2h**	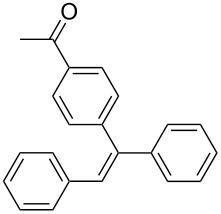 **3ah**	0.05	120	93

^a^Reaction conditions: 4-bromoacetophenone (**1a**, 1.0 mmol), alkenes **2** (1.2 mmol), NaOEt (2.0 mmol), Na_2_PdCl_4_ (0.05–0.1 mol %, 0.1% aqueous solution), **L1** (0.05–0.1 mol %, 0.1% aqueous solution), 1.5 mL H_2_O, 100 °C, 12 h, purged with N_2_. The mixture of **L1**, Na_2_PdCl_4_ and base in water was preheated in water at 60 °C for 30 min before adding substrates **1a** and **2**. ^b^Isolated yields. ^c^3.0 Equivalents of NaOEt was used.

**Figure 3:**
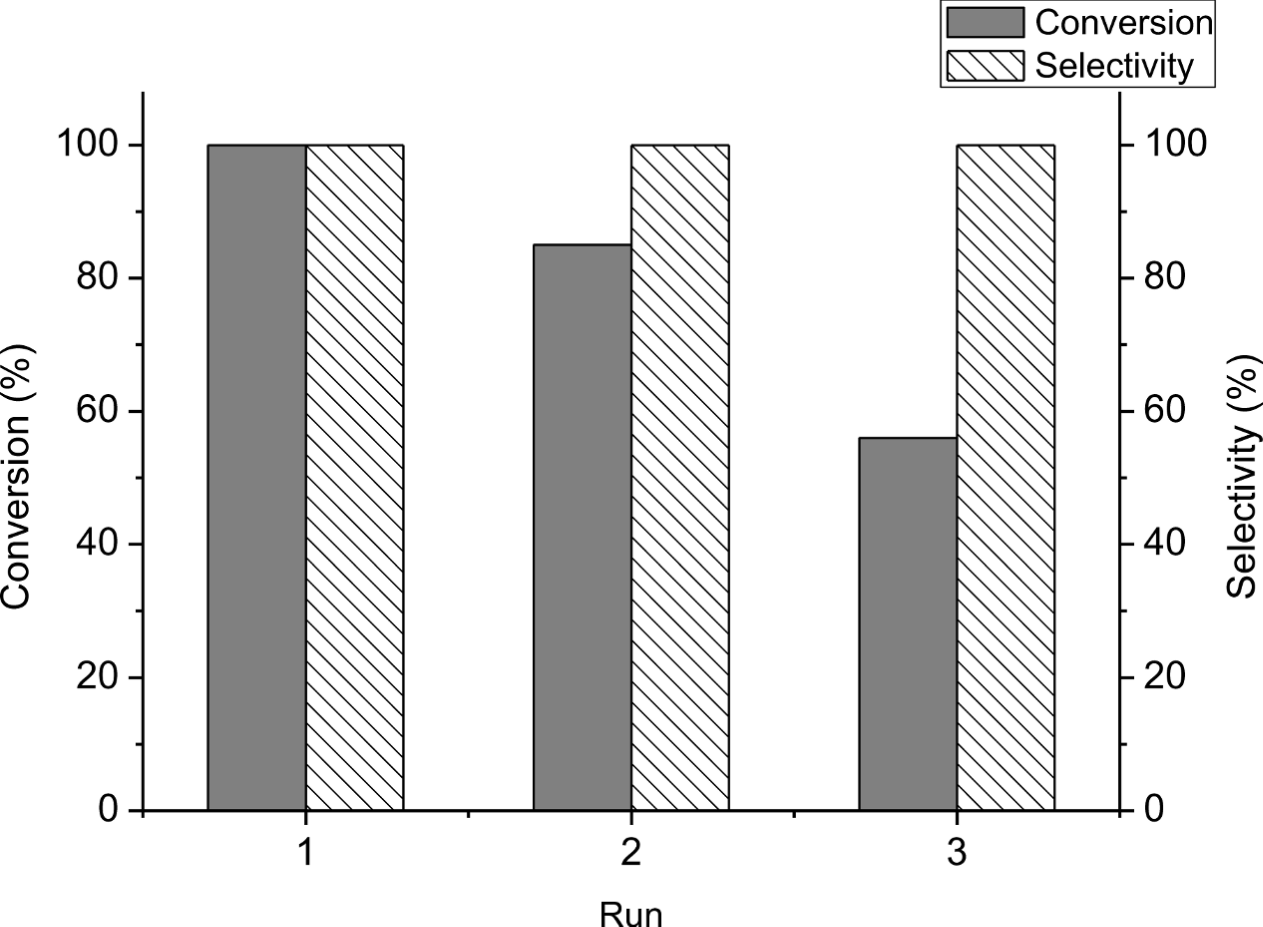
Reusability of the Na_2_PdCl_4_/**L1** catalytic system for the catalytic Mizoroki–Heck coupling reaction of 4-bromoacetophene (**1a**) and styrene (**2a**).

One of the important advantages of using water-soluble catalysts for reactions in water is the easy isolation of products by extraction with a water immiscible solvent, while retaining the catalyst in the aqueous phase for recovery and potential reuse. Therefore, the recyclability of the Na_2_PdCl_4_/**L1** catalytic system for Mizoroki–Heck reactions in water was examined by using the coupling of 4-bromoacetophenone (**1a**) and styrene (**2a**) under the optimal conditions as a model reaction. After each cycle, the yielded coupling product was extracted with MTBE. Then, fresh 4-bromoacetophenone, styrene and base were added into the catalyst-containing aqueous phase for further reaction. The results in Figure 3 show that the conversion of 4-bromoacetophenone was 85% for first recycle and 56% for second recycle, while the selectivity of (*E*)-4-acetylstilbene (**3aa**) was unchanged (>99%), which revealed that the catalytic system still remained certain catalytic activity.

## Conclusion

In summary, we have developed three PEG-functionalized imidazolium salts **L1**–**L3** from commercially available MeO-PEG_1900_-OH, imidazole, and various arylmethyl bromides (2-bromomethylpyridine for **L1**, benzyl bromide for **L2** and 1-bromomethylnaphthalene for **L3**). It was shown that these imidazolium salts **L1–L3** could be utilized as water soluble NHC ligand precursors in combination with Na_2_PdCl_4_ to form in situ the corresponding Pd–NHC catalysts for Mizoroki–Heck reactions in water without any organic co-solvent or phase transfer reagent. The results indicate that **L1** bearing a side-armed pyridine at N3-position of the imidazole ring exhibited the best catalytic activity in Mizoroki–Heck reactions, in which the pyridine group might serve as a hemilable donating functional group in the catalytic process. For the coupling of 4-bromoacetophenone and styrene, the TON of Na_2_PdCl_4_/**L1** catalytic system reached up to 10,000. Under the optimal conditions, large amounts of substituted alkenes were obtained in good to excellent yields using the Na_2_PdCl_4_/**L1** catalyst system with only a 0.05–0.1 mol % palladium loading. To the best of our knowledge, the catalyst loading in the current report for aqueous Mizoroki–Heck couplings of aryl bromides is much lower than other previously reported counterparts. Moreover, imidazolium salt **L1** was conveniently synthesized from commercially available materials. This newly developed protocol provides an efficient, practical and environmental benign method for the construction of various alkene derivatives.

## Experimental

### General

All chemicals were reagent grade and used as purchased. Monomethylated PEG_1900_ (MeO-PEG_1900_-OH) was obtained from Meryer Chem. Tech. Co. Ltd, China. All proton and ^13^C nuclear magnetic resonance (NMR) spectra were recorded on Bruker AVANCE III 500 MHz spectrometer in deuterated solvents with tetramethylsilane (TMS) as internal standard. Mass spectrometry data (MALDI–TOF) of the three imidazolium salts **L1**–**L3** were collected on a Bruker ultrafleXtreme mass spectrometer. Low-resolution mass analyses were performed on a Thermo Trace ISQ GC–MS instrument in EI mode (70 eV) or a Thermo Scientific ITQ 1100TM mass spectrometer in ESI mode. High-resolution mass spectra were recorded in the EI mode on a Waters GCT Premier TOF mass spectrometer with an Agilent 6890 gas chromatography using a DB-XLB column (30 m × 0.25 mm (i.d.), 0.25 μm). Melting points (uncorrected) were determined on a Büchi M-565 apparatus. Gas chromatography (GC) analyses were performed on Shimadzu GC-20A instrument with FID detector using a RTX-5 capillary column (30 m × 0.32 mm (i.d.), 0.25 μm). Flash column chromatography was performed on silica gel (200–300 mesh) with petroleum ether/ethyl acetate as eluent. De-ionized water was used in all reactions.

### Preparation of PEG-functionalized imidazolium salts **L1**, **L2** and **L3**

#### Synthesis of MeO-PEG_1900_-OMs

MeO-PEG_1900_-OH (38.0 g , 0.02 mol) and pyridine (3.16 g, 0.04 mol) were dissolved in 50 mL of dry DCM at an ice-water bath and under N_2_ atmosphere, followed by adding dropwise a solution of methanesulfonyl chloride (MsCl, 4.58 g, 0.04 mol) in 200 mL of dry DCM. After completion of addition, the mixture was stirred at room temperature for 24 h. The reaction was quenched with 100 mL of ice-water and the pH was adjusted to 7 with a 20% aqueous NaOH solution. The organic layer was separated, washed with water, dried with Na_2_SO_4_ and filtered. After removal of the solvent under vacuum, the residual was precipitated with methyl *tert*-butyl ether (MTBE) to afford 38.3 g (97%) of MeO-PEG_1900_-OMs as a white solid. ^1^H NMR (CDCl_3_) δ 4.34–4.32 (m, 2H, CH_2_OMs), 3.74–3.44 (m, 198H, CH_2_ of PEG chain), 3.33 (s, 3H, PEG-OCH_3_), 3.04 (s, 3H, SO_2_CH_3_); ^13^C NMR (CDCl_3_) δ 71.9–68.2 (C_PEG_), 60.7, 58.2, 36.8.

#### Synthesis of MeO-PEG_1900_-Im

To a solution of imidazole (0.89 g, 13 mmol) in 120 mL of dry THF at room temperature under N_2_ atmosphere was added NaH (60% dispersion in mineral oil, 0.8 g, 20 mmol). The mixture was then heated to 40 °C for 1 h to ensure the completion of H_2_ releasing. After that, MeO-PEG_1900_-OMs (19.7 g, 10 mmol) was added and the mixture was refluxed for 24 h. Then, the resulting suspension was filtered off and the filtrate was concentrated under vacuum. Precipitation with MTBE afforded 18.2 g (93%) of MeO-PEG_1900_-Im as a light yellow solid. ^1^H NMR (CDCl_3_) δ 7.50 (s, 1H, CH_imid_), 6.96 (s, 1H, CH_imid_), 6.95 (s, 1H, CH_imid_), 4.05 (t, *J* = 5.2 Hz, 2H, OCH_2_), 3.68 (t, *J* = 5.2 Hz, 2H, NCH_2_), 3.58–3.42 (m, 196H, CH_2_ of PEG chain), 3.30 (s, 3H, PEG-OCH_3_); ^13^C NMR (CDCl_3_) δ 136.8, 128.2, 118.8, 71.2–69.8 (C_PEG_), 58.2, 46.3.

#### Synthesis of imidazolium salts **L1**, **L2** and **L3**

A mixture of MeO-PEG_1900_-Im (3.9 g, 2 mmol) and the corresponding organic bromide (2.4 mmol) was heated in a sealed tube at 100 ^o^C for 24 h. The resulting imidazolium salts was isolated by precipitation with MTBE.

Imidazolium salt **L1**. Yield: 3.9 g (92%), pale brown solid; ^1^H NMR (DMSO-*d*_6_) δ 9.41 (s, 1H, CH_imid_), 8.56 (d, *J* = 4.2 Hz, 1H, CH_pyri_), 7.92–7.88 (m, 1H, CH_pyri_), 7.84 (s, 2H, CH_pyri_), 7.53 (d, *J* = 7.8 Hz, 1H, CH_imid_), 7.41 (d, *J* = 7.1 Hz, 1H, CH_imid_), 5.64 (s, 2H, CH_benzyl_), 4.43 (t, *J* = 4.7 Hz, 2H, OCH_2_), 3.81 (t, *J* = 4.7 Hz, 2H, NCH_2_), 3.66–3.42 (m, 196H, CH_2_ of PEG chain), 3.24 (s, 3H, PEG-OCH_3_); ^13^C NMR (CDCl_3_) δ 153.7, 149.6, 137.6, 137.3, 123.7, 123.0, 122.9, 122.7, 71.2–68.3 (C_PEG_), 58.1, 53.0, 49.0; MALDI–TOF–MS *m*/*z*: [*M*_n=49_ − Br]^+^ calcd for C_110_H_212_N_3_O_50_, 2375.4; found, 2375.8.

Imidazolium salt **L2**. Yield: 3.9 g (92%), pale white solid; ^1^H NMR (DMSO-*d*_6_) δ 9.28 (s, 1H, CH_imid_), 7.85–7.80 (m, 2H, CH_Ar_), 7.44–7.40 (m, 5H, CH_Ar_), 5.46 (s, 2H, CH_benzyl_), 4.38 (t, *J* = 4.6 Hz, 2H, OCH_2_), 3.79 (t, *J* = 4.6 Hz, 2H, NCH_2_), 3.51–3.42 (m, 196H, CH_2_ of PEG chain), 3.24 (s, 3H, PEG-OCH_3_); ^13^C NMR (DMSO-*d*_6_) δ 136.6, 135.0, 128.9, 128.7, 128.4, 123.1, 122.2, 71.34–68.2 (C_PEG_), 58.0, 51.7, 49.0; MALDI–TOF–MS *m*/*z*: [*M*_n=49_ − Br]^+^ calcd for C_111_H_213_N_2_O_50_, 2374.4; found, 2374.8.

Imidazolium salt **L3**. Yield: 3.8 g (88%), pale white solid; ^1^H NMR (DMSO-*d*_6_) δ 9.28 (s, 1H, CH_imid_), 8.15 (d, *J* = 8.0 Hz, 1H, CH_Ar_), 8.04–8.03 (m, 2H, CH_Ar_), 7.84 (s, 1H, CH_Ar_), 7.80 (s, 1H, CH_Ar_), 7.64–7.57 (m, 3H, CH_Ar_), 7.52 (d, *J* = 6.9 Hz, 1H, CH_imid_), 5.98 (s, 2H, CH_benzyl_), 4.36 (t, *J* = 2.4 Hz, 2H, OCH_2_), 3.76 (t, *J* = 4.7 Hz, 2H, NCH_2_), 3.51–3.41 (m, 196H, CH_2_ of PEG chain), 3.24 (s, 3H, PEG-OCH_3_); ^13^C NMR (DMSO-*d*_6_) δ 136.7, 133.5, 130.5, 130.2, 129.7, 128.9, 127.8, 127.2, 126.4, 125.6, 123.02, 122.97, 122.5, 71.3–68.1 (C_PEG_), 58.0, 49.8, 49.0; MALDI–TOF–MS *m*/*z*: [*M*_n=49_ − Br]^+^ calcd for C_115_H_215_N_2_O_50_, 2424.4; found, 2424.9.

#### General procedure for Mizoroki–Heck reactions in water

To a 10 mL tube, Na_2_PdCl_4_ (0.1% aqueous solution, 0.05–0.1 mol %), imidazolium salts **L1**–**L3** (1% aqueous solution, 0.05–0.1 mol %), NaOEt (2.0 mmol) and 1.5 mL water were successively added, followed by preheating at 60 °C for 30 min. Then, aryl bromide (1.0 mmol) and styrene (1.2 mmol) were added, purged with N_2_, sealed and heated at 100 °C. After 12 h, the solution was extracted with MTBE (5 mL × 2) and the organic layers combined, dried over anhydrous Na_2_SO_4_, and concentrated under vacuum. Finally, the resulted residual were purified by flash chromatography on silica to afford the desired cross-coupling alkene products. The purity of the obtained products was confirmed by NMR and the yields were based on aryl bromides.

## Supporting Information

File 1 Characterization data of Mizoroki–Heck products and copies of NMR spectra.
