# Supplementary material for: An efficient Pd–NHC catalyst system in situ generated from Na2PdCl4 and PEG-functionalized imidazolium salts for Mizoroki–Heck reactions in water
Source: Beilstein J Org Chem. 2017 Aug 21;13:1735–44. doi: 10.3762/bjoc.13.168 (PMC5588629; doi:10.3762/bjoc.13.168)
Supplement: File 1 — Characterization data of Mizoroki–Heck products and copies of NMR spectra. [file Beilstein_J_Org_Chem-13-1735-s001.pdf]

Supporting Information

for

An efficient Pd–NHC catalyst system in situ generated from Na_2PdCl_4 and PEG-functionalized imidazolium salts for Mizoroki–Heck reactions in water

Nan Sun*, Meng Chen, Liqun Jin, Wei Zhao, Baoxiang Hu, Zhenlu Shen and Xinquan Hu*

Address: College of Chemical Engineering, Zhejiang University of Technology, Hangzhou 310032, China

Email: Nan Sun - sunnan@zjut.edu.cn; Xinquan Hu - xinquan@zjut.edu.cn

* Corresponding author

Characterization data of Mizoroki–Heck products and copies of NMR spectra

Table of Contents

Characterization data for coupling products……………………………………………… S2

MALDI–TOF–MS spectra of **L1**, **L2** and **L3**……………………………………………… S9

Proton NMR and ^{13}C NMR spectra...................................………………….......................... S10

(E)-4-Acetylstilbene (3aa) (CAS No: 3112-03-6): Yield 96%, yellowish solid, mp: 141 ^{o}C (lit. [1] mp: 141.3-142 ^{o}C); ^{1}H NMR ($CDCl_3$) δ 7.97 (d, *J* = 8.3 Hz, 2H), 7.61 (d, *J* = 8.3 Hz, 2H), 7.56 (d, *J* = 7.5 Hz, 2H), 7.42-7.39 (m, 2H), 7.34-7.31 (m, 1H), 7.25 (d, *J* = 16.4 Hz, 1H), 7.15 (d, *J* = 16.4 Hz, 1H), 2.63 (s, 3H); ^{13}C NMR ($CDCl_3$) δ 197.5, 142.0, 136.7, 136.0, 131.5, 128.9, 128.8, 128.3, 127.5, 126.8, 126.5, 26.6; GC-EIMS *m/z*: 222 [M^+, 55%], 207 (100%).

(E)-4-Formylstilbene (3ba) (CAS No: 40200-69-9): Yield 98%, yellow solid, mp 112 ^{o}C (lit. [1] mp: 116.0-116.8 ^{o}C); ^{1}H NMR ($CDCl_3$) δ 10.01 (s, 1H), 7.89 (d, *J* = 8.2 Hz, 2H), 7.68 (d, *J* = 8.2 Hz, 2H), 7.57 (d, *J* = 7.4 Hz, 2H), 7.41 (t, *J* = 7.6 Hz, 2H), 7.33 (t, *J* = 7.4 Hz, 1H), 7.28 (d, *J* = 16.4 Hz, 1H), 7.16 (d, *J* = 16.4 Hz, 1H); ^{13}C NMR ($CDCl_3$) δ 191.6, 143.5, 136.6, 135.4, 132.2, 130.3, 128.9, 128.5, 127.4, 126.9; GC-EIMS *m/z*: 208 [M^+, 90%], 179 (100%).

(E)-4-Nitrostilbene (3ca) (CAS No: 1694-20-8): Yield 95%, yellow solid, mp 157 ^{o}C (lit. [1] mp: 154.2-155 ^{o}C); ^{1}H NMR ($CDCl_3$) δ 8.13 (d, *J* = 8.8 Hz, 2H), 7.55 (d, *J* = 8.8 Hz, 2H), 7.47 (d, *J* = 7.5 Hz, 2H), 7.32 (t, *J* = 7.5 Hz, 2H), 7.25 (t, *J* = 7.3 Hz, 1H), 7.19 (d, *J* = 16.3 Hz, 1H), 7.06 (d, *J* = 16.3 Hz, 1H); ^{13}C NMR ($CDCl_3$) δ 146.8, 143.9, 136.2, 133.4, 128.9, 128.8, 127.0, 126.9, 126.3, 124.2; GC-EIMS *m/z* 225: [M^+, 55%], 179 (100%).

(E)-4-Trifluoromethylstilbene (3da) (CAS No: 1149-56-0): Yield 94%, white solid, mp 132 ^{o}C (lit. [1] mp: 132.1-133.4 ^{o}C); ^{1}H NMR ($CDCl_3$) δ 7.69-7.60 (m, 4H), 7.57 (d, *J* = 7.4 Hz, 2H), 7.42 (t, *J* = 7.6 Hz, 2H), 7.35 (t, *J* = 7.3 Hz, 1H), 7.23 (d, *J* = 16.4 Hz, 1H), 7.15 (d, *J* = 16.4 Hz, 1H); ^{13}C NMR ($CDCl_3$) δ 140.8, 136.6, 131.2, 129.2 (q, *J* = 32.2 Hz), 128.8, 128.3, 127.1, 126.8, 126.6, 125.6 (q, *J* = 3.8 Hz), 124.3 (q, *J* = 270.0 Hz); GC-EIMS *m/z*: 248 [M^+, 91%], 179 (100%).

(E)-4-Fluorostilbene (3ea) (CAS No: 718-25-2): Yield 87%, white solid, mp 120 ^{o}C (lit. [2] mp: 123-124 ^{o}C); ^{1}H NMR ($CDCl_3$) δ 7.37 (m, 4H), 7.25 (m, 2H), 7.16 (m, 1H), 6.93 (m, 4H); ^{13}C NMR

($CDCl_3$) δ 162.3 (d, *J* = 246.3 Hz), 137.2, 133.5 (d, *J* = 3.8 Hz), 128.7, 128.5, 128.0 (d, *J* = 7.5 Hz), 127.7, 127.5, 126.4, 115.6 (d, *J* = 21.3 Hz); GC-EIMS *m/z*: 198 [M^+, 100%].

(E)-4-Chlorostilbene (3fa) (CAS No: 1657-50-7): Yield 90%, white solid, mp 127 ^oC (lit. [3] mp: 126.2-128.3 ^oC); ^1H NMR ($CDCl_3$) δ 7.56-7.50 (m, 2H), 7.49-7.43 (m, 2H), 7.39 (t, *J* = 7.7 Hz, 2H), 7.36-7.32 (m, 2H), 7.32-7.28 (m, 1H), 7.14-7.04 (m, 2H); ^{13}C NMR ($CDCl_3$) δ 137.0, 135.9, 133.2, 129.3, 128.9, 128.8, 127.9, 127.7, 127.4, 126.6; GC-EIMS *m/z*: 214 [M^+, 63%], 216 [M+2, 21%], 179 (100%).

(E)-4-Bromostilbene (3ga) (CAS No: 13041-70-8): Yield 87%, white solid, mp 137 ^oC (lit. [3] mp: 136.5-139 ^oC); ^1H NMR ($CDCl_3$) δ 7.54-7.49 (m, 4H), 7.44-7.36 (m, 4H), 7.31 (t, *J* = 7.4 Hz, 1H), 7.13 (d, *J* = 16.4 Hz, 1H), 7.06 (d, *J* = 16.4 Hz, 1H); ^{13}C NMR ($CDCl_3$) δ 137.0, 136.3, 131.8, 129.4, 128.7, 128.0, 127.9, 127.4, 126.6, 121.3; GC-EIMS *m/z*: 258 [M^+, 46%], 260 [M+2, 47%], 179 (100%).

(E)-Stilbene (3ha) (CAS No: 103-30-0): Yield 76%, white solid, mp 122 ^oC (lit. [1] mp: 123.9-124.6 ^oC); ^1H NMR ($CDCl_3$) δ 7.57 (d, *J* = 7.5 Hz, 4H), 7.41 (t, *J* = 7.5 Hz, 4H), 7.32 (d, *J* = 7.4 Hz, 2H), 7.17 (s, 2H); ^{13}C NMR ($CDCl_3$) δ 137.3, 128.7, 127.6, 126.5; GC-EIMS *m/z*: 180 [M+, 100%].

(E)-4-Methylstilbene (3ia) (CAS No: 1860-17-9): Yield 88%, white solid, mp 118 ^oC (lit. [1] mp: 121.7-122.3 ^oC); ^1H NMR ($CDCl_3$) δ 7.55 (d, *J* = 8.3 Hz, 2H), 7.46 (d, *J* = 8.3 Hz, 2H), 7.40 (t, *J* = 7.7 Hz, 2H), 7.29 (t, *J* = 7.2 Hz 1H), 7.21 (d, *J* = 7.9 Hz, 2H), 7.16-7.08 (m, 2H), 2.40 (s, 3H); ^{13}C NMR ($CDCl_3$) δ 137.5, 134.6, 129.4, 128.65, 128.62, 127.7, 127.4, 126.43, 126.40, 21.3; GC-EIMS *m/z*: 194 [M^+, 79%], 179 (100%).

(E)-4-Methoxostilbene (3ja) (CAS No: 1694-19-5): Yield 53%, white solid, mp 128 ^oC (lit. [1] mp: 135.3-135.9 ^oC); ^1H NMR ($CDCl_3$) δ 7.52-7.46 (m, 4H), 7.37 (t, *J* = 7.7 Hz, 2H), 7.25 (t, *J* = 7.4 Hz, 1H), 7.09 (d, *J* = 16.4 Hz, 1H), 7.00 (d, *J* = 16.4 Hz, 1H), 6.94-6.88 (m, 2H), 3.85 (s, 3H); ^{13}C NMR

($CDCl_3$) δ 159.3, 137.7, 130.2, 128.7, 128.2, 127.7, 127.2, 126.6, 126.3, 114.2, 55.4; GC-EIMS *m/z*: 210 [M+, 100%].

(E)-4-Aminostilbene (3ka) (CAS No: 4309-66-4): Yield 87%, yellowish solid, mp 149 ^{o}C (lit. [4] mp: 147-148 ^{o}C); ^{1}H NMR ($CDCl_3$) δ 7.49 (d, *J* = 7.5 Hz, 2H), 7.32 (t, *J* = 7.7 Hz, 2H), 7.28 (d, *J* = 8.4 Hz, 2H), 7.18 (t, *J* = 7.3 Hz, 1H), 7.06 (d, *J* = 16.4 Hz, 1H), 6.89 (d, *J* = 16.4 Hz, 1H), 6.56 (d, *J* = 8.4 Hz, 2H), 5.33 (s, 2H); ^{13}C NMR ($CDCl_3$) δ 148.8, 138.0, 129.1, 128.6, 127.7, 126.5, 125.8, 124.7, 122.8, 113.9; GC-EIMS *m/z*: 195 [M+, 100%].

(E)-4-Hydroxystilbene (3la) (CAS No: 6554-98-9): Yield 65%, yellowish solid, mp 188 ^{o}C (lit. [5] mp: 185-187 ^{o}C); ^{1}H NMR (DMSO) δ 9.58 (s, 1H), 7.54 (d, *J* = 8.5 Hz, 2H), 7.43 (d, *J* = 8.5 Hz, 2H), 7.35 (t, *J* = 7.7 Hz, 2H), 7.22 (t, *J* = 7.3 Hz, 1H), 7.15 (d, *J* = 16.4 Hz, 1H), 7.02 (d, *J* = 16.4 Hz, 1H), 6.79-6.76 (m, 2H); ^{13}C NMR (DMSO) δ 157.3, 137.5, 128.6, 128.4, 128.0, 127.8, 126.9, 126.0, 125.1, 115.5; ESI-MS: *m/z* 195 [$(M-H)^-$, 100%].

(E)-3-Acetylstilbene (3ma) (CAS No: 116702-87-5): Yield 91%, white solid, mp 78 ^{o}C (lit. [6] mp: 70-74 ^{o}C); ^{1}H NMR ($CDCl_3$) δ 8.12 (s, 1H), 7.86 (d, *J* = 7.7 Hz, 1H), 7.73 (d, *J* = 7.7 Hz, 1H), 7.55 (d, *J* = 7.5 Hz, 2H), 7.48 (t, *J* = 7.7 Hz, 1H), 7.40 (t, *J* = 7.6 Hz, 2H), 7.28 (t, *J* = 7.4 Hz, 1H), 7.22 (d, *J* = 16.4 Hz, 1H), 7.16 (d, *J* = 16.4 Hz, 1H), 2.67 (s, 3H); ^{13}C NMR ($CDCl_3$) δ 198.1, 137.9, 137.6, 136.9, 130.9, 130.1, 129.0, 128.8, 128.0, 127.6, 127.5, 126.7, 126.2, 26.6; GC-EIMS *m/z*: 222 [M^+, 64%], 179 (100%).

(E)-3-Formylstilbene (3na) (CAS No: 71093-83-9): Yield 89%, yellowish solid, mp 103 ^{o}C (lit. [7] mp: 101-103 ^{o}C); ^{1}H NMR ($CDCl_3$) δ 10.07 (s, 1H), 8.04 (s, 1H), 7.77 (t, *J* = 6.9 Hz, 2H), 7.58-7.52 (m, 3H), 7.41 (t, *J* = 7.6 Hz, 2H), 7.32 (t, *J* = 7.3 Hz, 1H), 7.23 (d, *J* = 16.4 Hz, 1H), 7.16 (d, *J* = 16.4 Hz, 1H); ^{13}C NMR (125 MHz) δ 192.3, 138.4, 136.8, 136.7, 132.3, 130.5, 129.4, 128.9, 128.8, 128.2, 127.2, 127.1, 126.7; GC-EIMS *m/z*: 208 [M^+, 88%], 179 (100%).

(E)-3-Methylstilbene (3oa) (CAS No: 14064-48-3): Yield 77%, white solid, mp 49 ^{o}C (lit. [2] mp: 48.6-49.2 ^{o}C); ^{1}H NMR ($CDCl_3$) δ 7.56-7.54 (m, 2H), 7.40-7.35 (m, 4H), 7.31-7.27 (m, 2H), 7.13-7.09 (m, 3H), 2.41 (s, 3H); ^{13}C NMR ($CDCl_3$) δ 138.2, 137.4, 137.3, 128.8, 128.7, 128.6, 128.49, 128.47, 127.6, 127.2, 126.5, 123.7, 21.5; GC-EIMS *m/z*: 194 [M^+, 90%], 179 (100%).

(E)-2-Formylstilbene (3qa) (CAS No: 52095-44-0): Yield 51%, yellow oil; ^{1}H NMR ($CDCl_3$) δ 10.35 (s, 1H), 8.07 (d, *J* = 16.2 Hz, 1H), 7.87 (d, *J* = 7.8 Hz, 1H), 7.75 (d, *J* = 7.8 Hz, 1H), 7.63-7.57 (m, 3H), 7.48-7.45 (m, 1H), 7.41 (d, *J* = 7.6 Hz, 2H), 7.35-7.32 (m, 1H), 7.08 (d, *J* = 16.2 Hz, 1H); ^{13}C NMR ($CDCl_3$) δ 192.7, 140.0, 136.9, 134.0, 133.7, 133.0, 132.3, 128.8, 128.3, 127.6, 127.2, 127.0, 124.8; GC-EIMS *m/z*: 208 [M^+, 100%].

(E)-2-Methylstilbene (3ra) (CAS No: 22257-16-5): Yield 73%, white solid, mp 34 ^{o}C (lit. [2] mp: 31-32 ^{o}C); ^{1}H NMR ($CDCl_3$) δ 7.64 (d, *J* = 7.3 Hz, 1H), 7.57 (d, *J* = 7.4 Hz, 2H), 7.42-7.36 (m, 3H), 7.31 (t, *J* = 7.4 Hz, 1H), 7.28-7.25 (m, 1H), 7.24-7.22 (m, 2H), 7.04 (d, *J* = 16.1 Hz, 1H), 2.47 (s, 3H); ^{13}C NMR ($CDCl_3$) δ 137.7, 136.4, 135.8, 130.4, 130.0, 128.7, 127.61, 127.56, 126.6, 126.2, 125.4, 19.9; GC-EIMS *m/z*: 194 [M^+, 76%], 179 (100%).

(E)-2-Styrylnaphthalene (3sa) (CAS No: 2840-89-3): Yield 84%, white solid, mp 145 ^{o}C (lit. [8] mp: 144-146 ^{o}C); ^{1}H NMR ($CDCl_3$) δ 7.91-7.81 (m, 4H), 7.80 (d, *J* = 8.6 Hz, 1H), 7.63 (d, *J* = 7.5 Hz, 2H), 7.55-7.50 (m, 2H), 7.45 (t, *J* = 7.6 Hz, 2H), 7.36-7.31 (m, 3H); ^{13}C NMR ($CDCl_3$) δ 137.4, 134.8, 133.7, 133.1, 129.0, 128.8, 128.75, 128.3, 128.0, 127.7, 127.6 126.6, 126.5, 126.3, 125.9, 123.5; GC-EIMS *m/z*: 230 [M^+, 100%].

(E)-3-Styrylpyridine (3ta) (CAS No: 5097-91-6): Yield 97%, white solid, mp 79 ^{o}C (lit. [9] mp: 78.6-81.6 ^{o}C); ^{1}H NMR ($CDCl_3$) δ 8.74 (s, 1H), 8,54-8.50 (m, 1H), 7.86-7.84 (m, 1H), 7.55 (d, *J* = 7.4 Hz, 2H), 7.40 (t, *J* = 7.6 Hz, 2H), 7.33-7.29 (m, 2H), 7.18 (d, *J* = 16.4 Hz, 1H), 7.09 (d, *J* = 16.4 Hz,

1H); ^{13}C NMR ($CDCl_3$) δ 148.6, 136.7, 133.0, 132.7, 130.9, 128.8, 128.2, 126.7, 124.9, 123.5; GC-EIMS *m/z*: 181 [M^+, 42%], 180 (100%).

(E)-3-Styrylquinoline (3ua) (CAS No: 61064-99-1): Yield 86%, white solid, mp 99 ^{o}C (lit. [10] mp: 92-93 ^{o}C); ^{1}H NMR ($CDCl_3$) δ 9.15 (s, 1H), 8.19 (s, 1H), 8.11 (d, *J* = 8.5 Hz, 1H), 7.84 (d, *J* = 8.2 Hz, 1H), 7.72-7.68 (m, 1H), 7.60 (d, *J* = 7.5 Hz, 2H), 7.57 (t, *J* = 3.8 Hz, 1H), 7.42 (t, *J* = 7.6 Hz, 2H), 7.34 (t, *J* = 3.7 Hz, 1H), 7.35 (d, *J* = 16.5 Hz, 1H), 7.26 (d, *J* = 16.4 Hz, 1H); ^{13}C NMR ($CDCl_3$) δ 149.5, 147.5, 136.8, 132.3, 130.9, 130.3, 129.3, 129.2, 128.8, 128.3, 128.1, 127.8, 127.0, 126.7, 125.2; GC-EIMS *m/z*: 231 [M^+, 76%], 230 (100%).

(E)-1-Acetyl-4-(4-methoxystyryl)benzene (3ab) (CAS No: 117760-54-0): Yield 97%, white solid, mp 171 ^{o}C (lit. [11] mp: 174.5-175 ^{o}C); ^{1}H NMR ($CDCl_3$) δ 7.95 (d, *J* = 8.3 Hz, 2H), 7.57 (d, *J* = 8.3 Hz, 2H), 7.50 (d, *J* = 8.7 Hz, 2H), 7.20 (d, *J* = 16.3 Hz, 1H), 7.01 (d, *J* = 16.3 Hz, 1H), 6.93 (d, *J* = 8.7 Hz, 2H), 3.86 (s, 3H), 2.62 (s, 3H); ^{13}C NMR ($CDCl_3$) δ 197.5, 159.9, 142.4, 135.6, 131.0, 129.5, 128.9, 128.2, 126.2, 125.3, 114.3, 55.4, 26.6; GC-EIMS *m/z*: 252 [M^+, 100%].

(E)-1-Acetyl-4-(4-methylstyryl)benzene (3ac) (CAS No: 1022914-04-0): Yield 95%, yellowish solid, mp 172 ^{o}C; ^{1}H NMR ($CDCl_3$) δ 7.96 (d, *J* = 8.4 Hz, 2H), 7.59 (d, *J* = 8.4 Hz, 2H), 7.45 (d, *J* = 8.1 Hz, 2H), 7.22 (d, *J* = 16.4 Hz, 1H), 7.21 (d, *J* = 7.9 Hz, 2H), 7.10 (d, *J* = 16.4 Hz, 1H), 2.62 (s, 3H), 2.39 (s, 3H); ^{13}C NMR ($CDCl_3$) δ 197.5, 142.3, 138.4, 135.8, 133.9, 131.4, 129.5, 128.9, 126.8, 126.4, 126.3, 26.6, 21.3; GC-EIMS *m/z*: 236 [M^+, 44%], 221 (100%).

(E)-1-Acetyl-4-(4-chlorostyryl)benzene (3ad) (CAS No: 63483-67-0): Yield 93%, yellow solid, mp 142 ^{o}C; ^{1}H NMR ($CDCl_3$) δ 7.97 (d, *J* = 8.4 Hz, 2H), 7.58 (d, *J* = 8.4 Hz, 2H), 7.47 (d, *J* = 8.5 Hz, 2H), 7.36 (d, *J* = 8.5 Hz, 2H), 7.18 (d, *J* = 16.4 Hz, 1H), 7.10 (d, *J* = 16.4 Hz, 1H), 2.62 (s, 3H); ^{13}C NMR ($CDCl_3$): δ 197.4, 141.6, 136.1, 135.2, 133.9, 130.1, 129.0, 128.9, 128.0, 127.9, 126.5, 26.6; GC-EIMS *m/z*: 256 [M^+, 53%], 258 [M+2, 18%], 241 (100%).

(E)-2-(4-Acetylstyryl)naphthalene (3ae): Yield 96%, yellowish solid, mp 166-167 °C; ^{1}H NMR ($CDCl_3$) δ 8.00 (d, *J* = 8.4 Hz, 2H), 7.92 (s, 1H), 7.87-7.84 (m, 3H), 7.89-7.77 (m, 1H), 7.66 (d, *J* = 8.4 Hz, 2H), 7.53-7.48 (m, 2H), 7.42 (d, *J* = 16.3 Hz, 1H), 7.28 (d, *J* = 16.3 Hz, 1H), 2.64 (s, 3H); ^{13}C NMR ($CDCl_3$) δ 197.3, 142.1, 138.8, 136.0, 134.2, 133.7, 133.4, 131.6, 129.0, 128.5, 128.2, 127.8, 127.4, 126.6, 126.3, 123.4, 26.6; EI-HRMS *m/z*: calcd $C_{20}H_{16}NO$ $[M]^+$: 272.1201 found: 272.1209.

(E)-3-(4-Acetylphenyl)acrylic acid 3(af) (CAS No: 116868-96-3): Yield 89%, yellowish solid, mp 211 °C (lit. [12] mp 224-226 °C); ^{1}H NMR (DMSO) δ 2.51 (s, 1H), 7.98 (d, *J* = 8.4 Hz, 2H), 7.84 (d, *J* = 8.4 Hz, 2H), 7.65 (d, *J* = 16.1 Hz, 1H), 6.68 (d, *J* = 16.1 Hz, 1H), 2.60 (s, 3H); ^{13}C NMR (DMSO) δ 197.9, 167.7, 143.0, 139.0, 138.04, 129.1, 128.9, 122.3, 27.3; ESI-MS *m/z*: 189 [$(M-H)^-$, 100%].

(E)-4-(4-Acetylstyryl)pyridine (3ag): Yield 85%, yellowish solid, mp 104 °C; ^{1}H NMR ($CDCl_3$) δ 8.63 (d, *J* = 6.0 Hz, 2H), 8.00 (d, *J* = 8.3 Hz, 2H), 7.64 (d, *J* = 8.3 Hz, 2H), 7.41 (d, *J* = 6.0 Hz, 2H), 7.34 (d, *J* = 16.4 Hz, 1H), 7.15 (d, *J* = 16.4 Hz, 1H), 2.64 (s, 3H); ^{13}C NMR ($CDCl_3$) δ 197.4, 150.3, 144.0, 140.6, 136.8, 131.9, 128.9, 128.7, 127.1, 121.0, 26.6; EI-HRMS *m/z* calcd $C_{15}H_{13}NO$ $[M]^+$: 223.0997 found: 223.1008.

(E)-α-(4-Acetylphenyl)stilbene (3ah): Yield 93%, yellowish solid, mp 135 °C; ^{1}H NMR ($CDCl_3$) δ 7.75 (d, *J* = 8.4 Hz, 2H), 7.39-7.33 (m, 8H), 7.23-7.21 (m, 2H), 7.12 (d, *J* = 8.4 Hz, 2H), 7.01 (s, 1H), 2.55 (s, 3H); ^{13}C NMR ($CDCl_3$) δ 197.5, 145.2, 142.9, 142.3, 139.8, 135.0, 130.2, 129.6, 128.8, 128.3, 128.1, 128.0, 127.8, 127.7, 127.0, 26.5; EI-HRMS *m/z* calcd $C_{22}H_{18}O$ $[M]^+$: 298.1358 found: 298.1371.

References:

1. Xu, H. J.; Zhao, Y. Q.; Zhou, X. F. *J. Org. Chem.* **2011**, *76*, 8036-8041.

2. Leng, Y. T.; Tang, F.; Wei, K.; Wu, Y. J. *Tetrahedron* **2010**, *66*, 1244-1248.

3. Kikukawa, K.; Naritomi, M.; He, G. X.; Wada, F.; Matsuda, T. *J. Org. Chem.* **1985**, *50*, 299-301.

4. Barder, T. E.; Walker, S. D.; Martinelli, J. R.; Buchwald, S. L. *J. Am. Chem. Soc.* **2005**, *127*,

4685-4696.

5. Sinha, A. K.; Kumar, V.; Sharma, A.; Sharma A.; Rakesh Kumar, R. *Tetrahedron* **2007**, *63* 11070-11077.

6. Hajipour, A. R.; Karami, K.; Pirisedigh, A.; Ruoho, A. *J. Organomet. Chem.* **2009**, *69*4, 2548-2554.

7. Castells, J.; Font, J.; Virgili, A. *J. Chem. Soc., Perkin Tran. 1: Org. Bio-org. Chem.* **1979**, 1-6.

8. Mochida, S.; Hirano, K.; Satoh, T.; Miura, M. *Org. Lett.* **2010**, *12*, 5776-5779.

9. Aksın, Ö.; Türkmen, H.; Artok, L.; Çetinkaya, B.; Ni, C. Büyükgiingörd, O.; Ozkal, E. *J. Organomet. Chem.* **2006**, *691* (2006), 3027-3036.

10. Loska, R.; Chandra, M.; Volla, R.; Vogel, P. *Adv. Synth. Catal.* **2008**, *350*, 2859-2864.

11. Dale, W. J.; Hennis, H. E. *J. Am. Chem. Soc.* **1959**, *81*, 2143-2146.

12. Fukuyama, T.; Arai, M.; Matsubara, H.; Ryu, I. *J. Org. Chem.* **2004**, *69*, 8105-8107.

MALDI–TOF–MS spectra of L1, L2 and L3

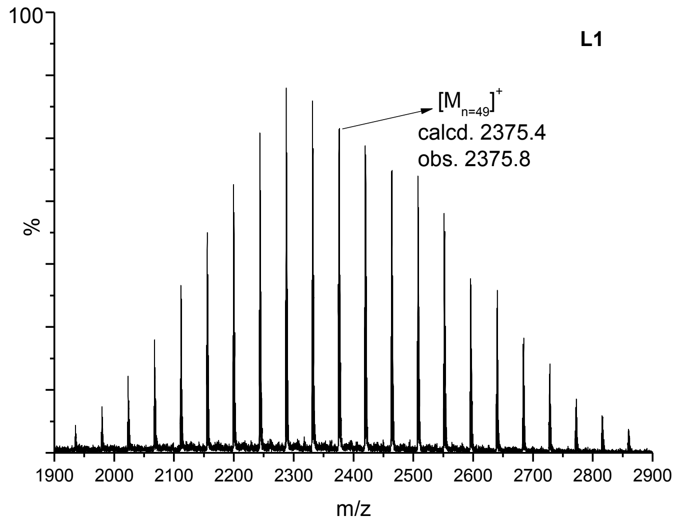

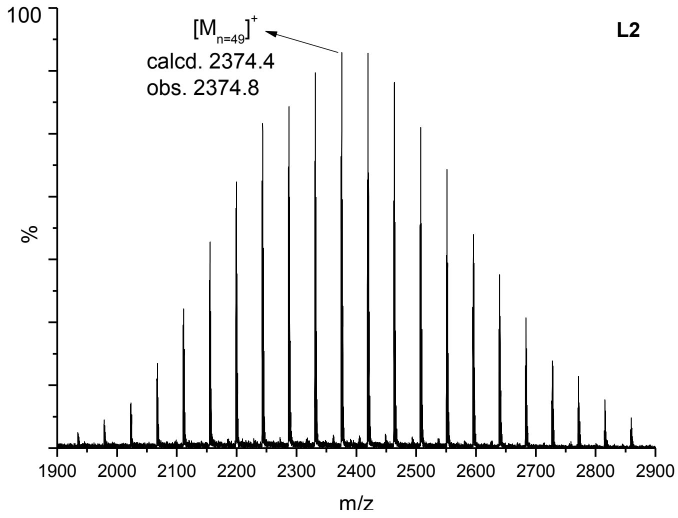

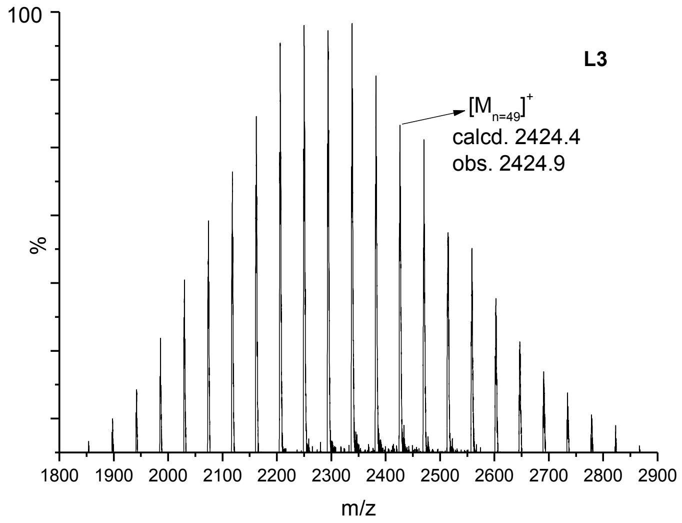

Proton NMR and ^{13}C NMR spectra

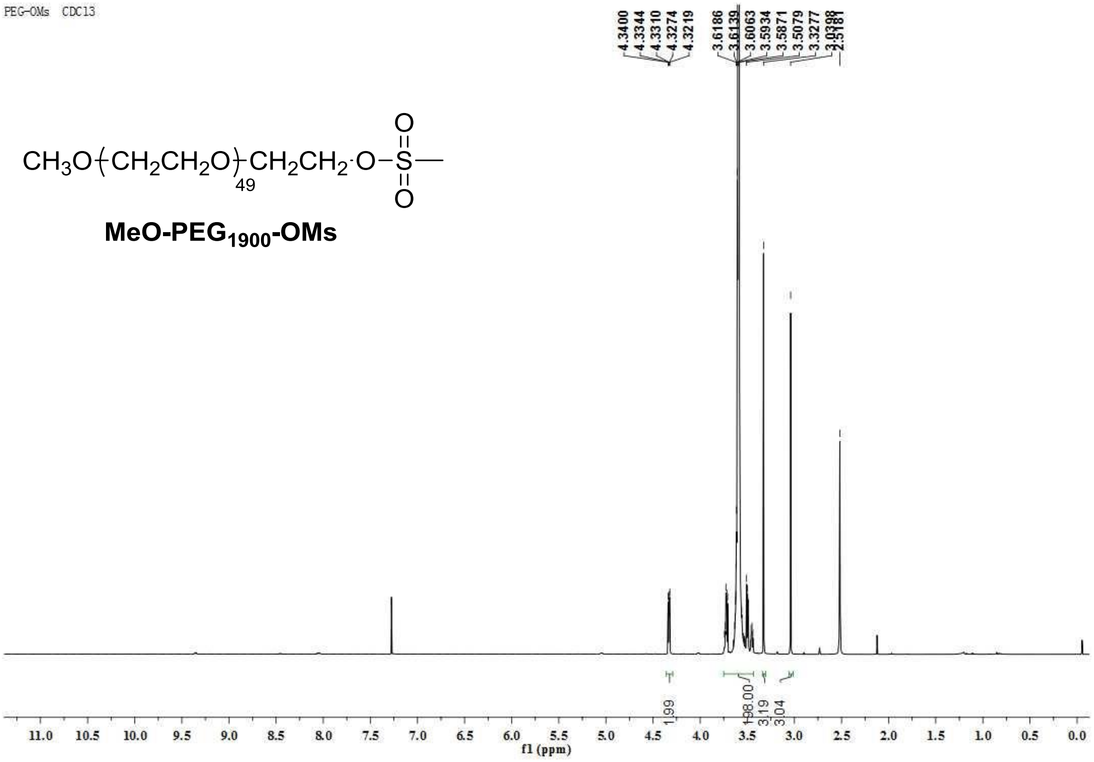

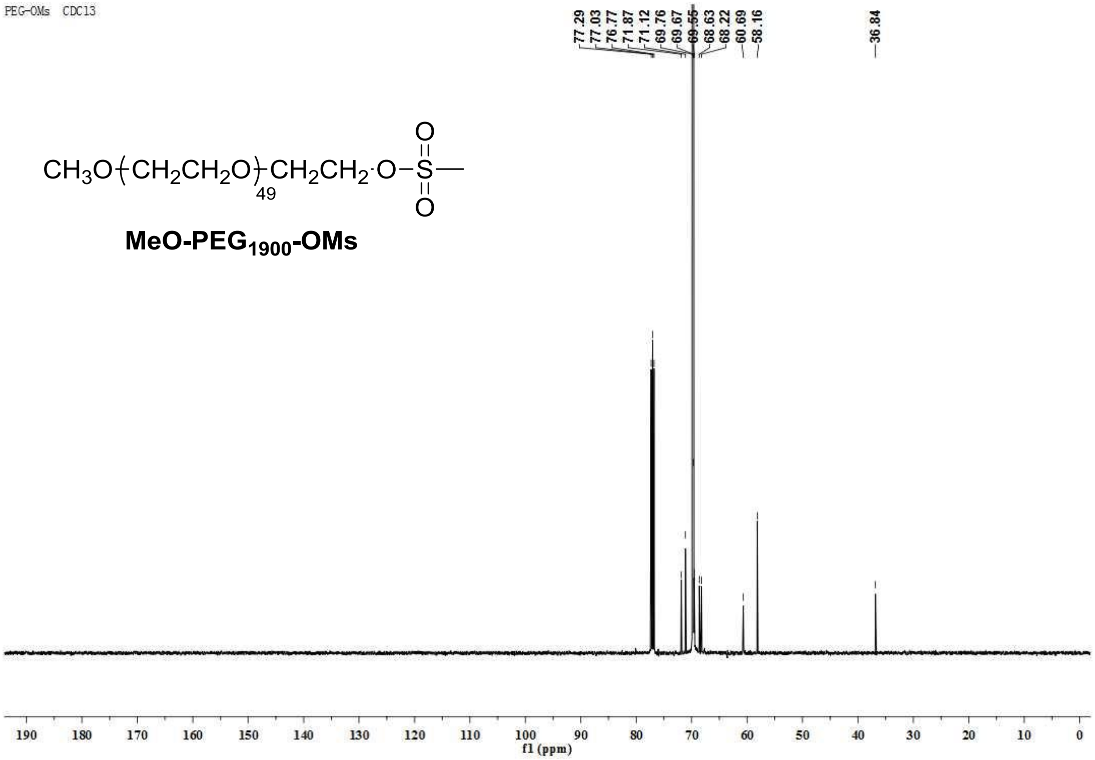

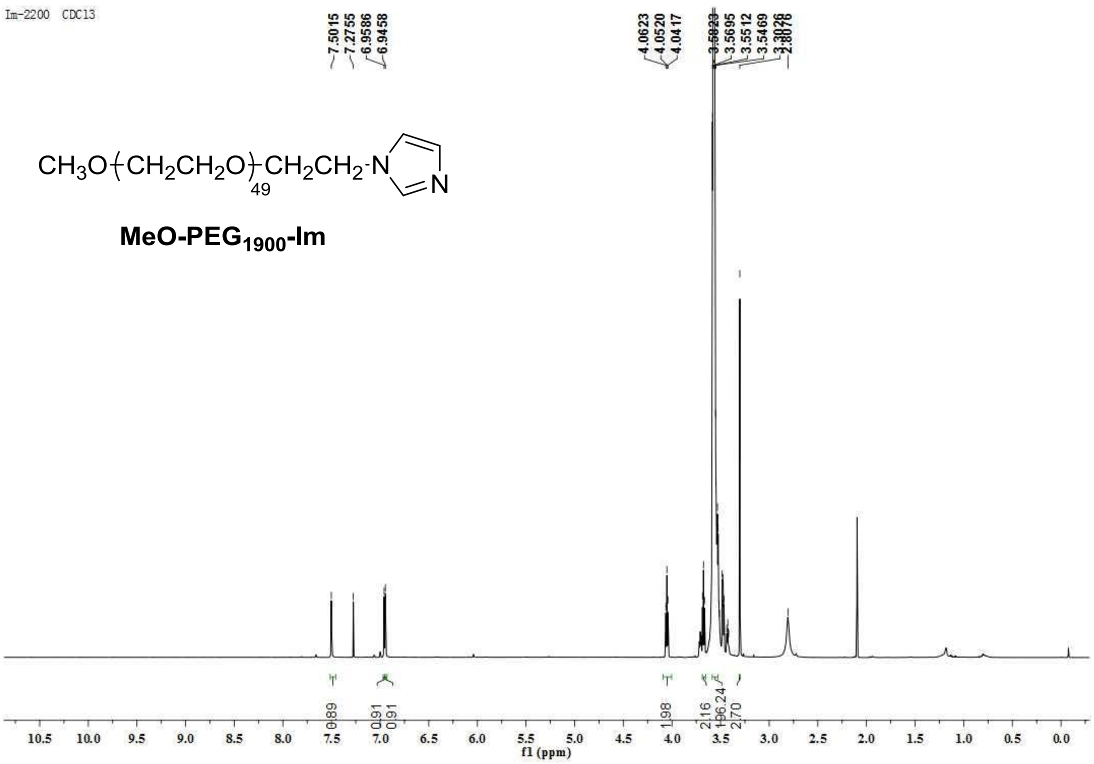
Im-2200 CDCl3
7.5015
7.2755
6.9586
6.9458
4.0623
4.0520
4.0417
3.5823
3.5695
3.5512
3.5469
2.8076
$CH_3O(CH_2CH_2O)_{49}CH_2CH_2$-N
N
MeO-PEG1900-Im
0.89
0.91
0.91
1.98
2.16
196.24
2.70
f1 (ppm)

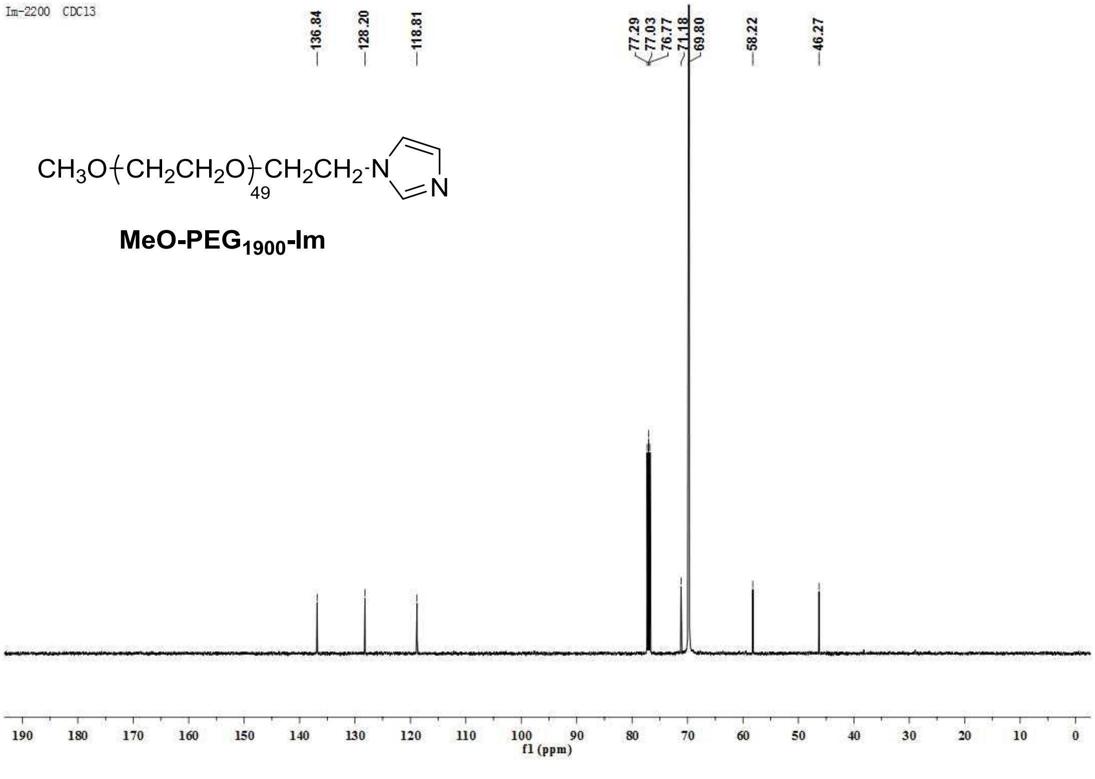
Im-2200 CDCl3
136.84
128.20
118.81
77.29
77.03
76.77
71.18
69.80
58.22
46.27
$CH_3O(CH_2CH_2O)_{49}CH_2CH_2$-N
N
MeO-PEG1900-Im
f1 (ppm)

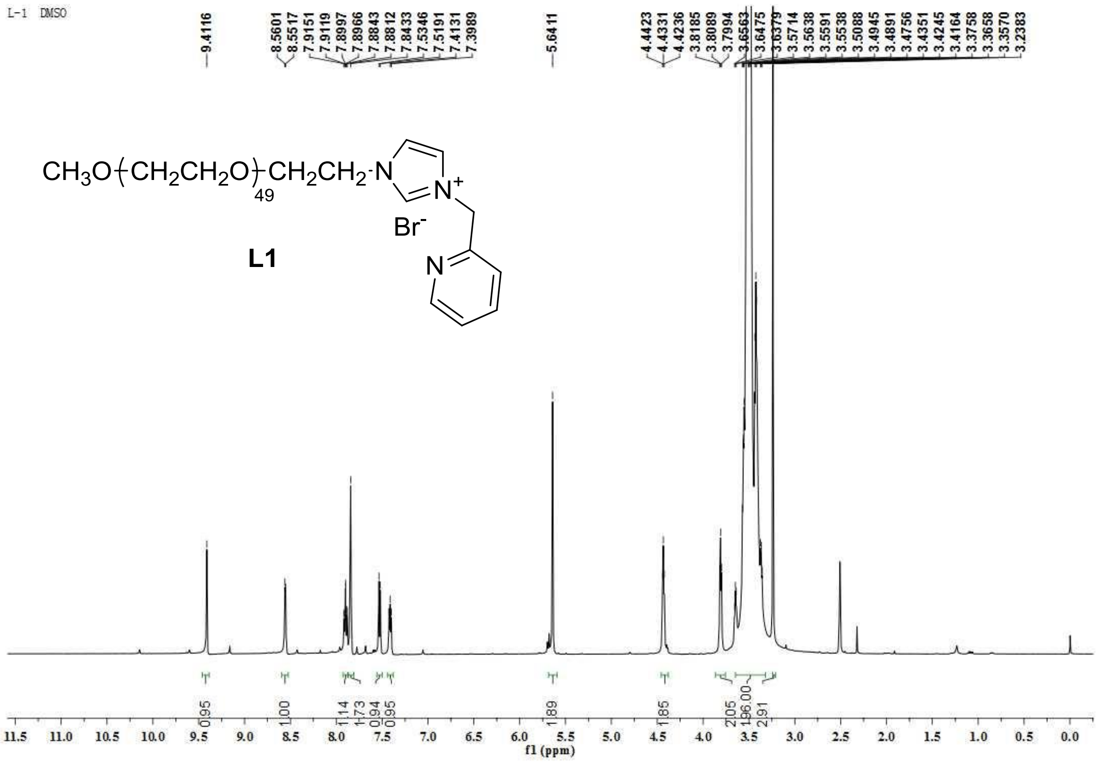
L-1 DMSO
L1
f1 (ppm)

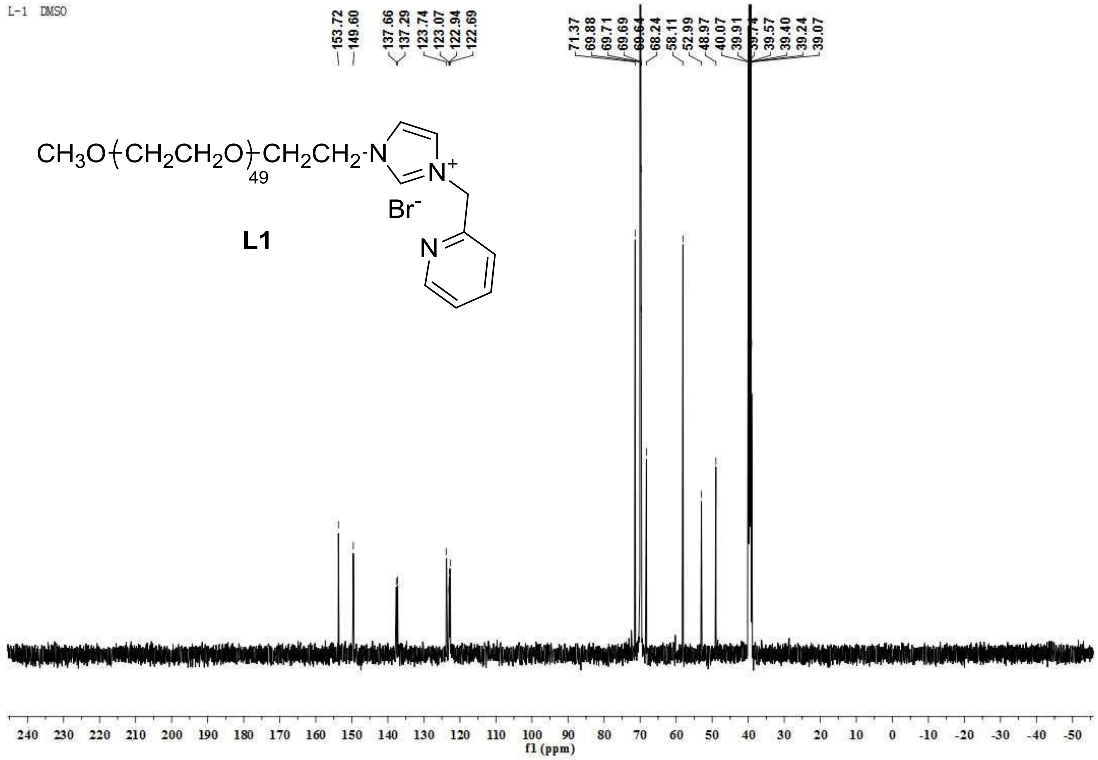
L-1 DMSO
L1
f1 (ppm)

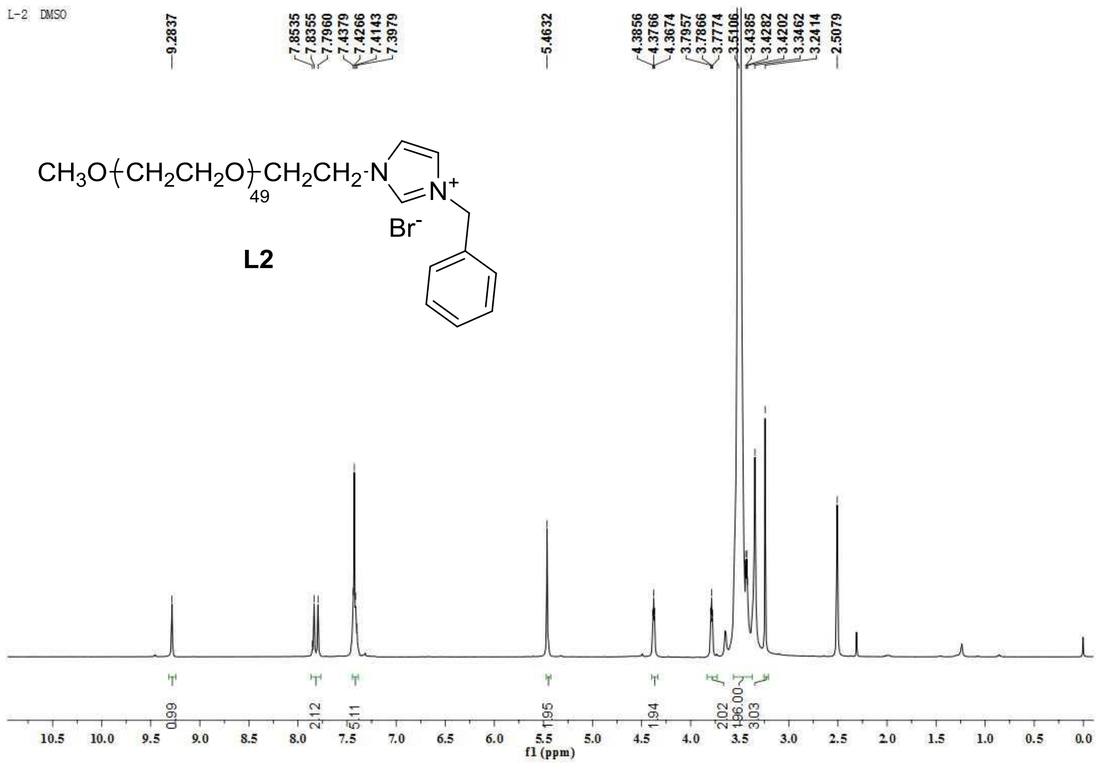
L-2 DMSO
9.2837
7.8535
7.8355
7.7960
7.4379
7.4266
7.4143
7.3979
5.4632
4.3856
4.3766
4.3674
3.7957
3.7866
3.7774
3.5106
3.4385
3.4282
3.4202
3.3462
3.2414
2.5079
L2
Br-
0.99
2.12
5.11
1.95
1.94
2.02
196.00
3.03
f1 (ppm)

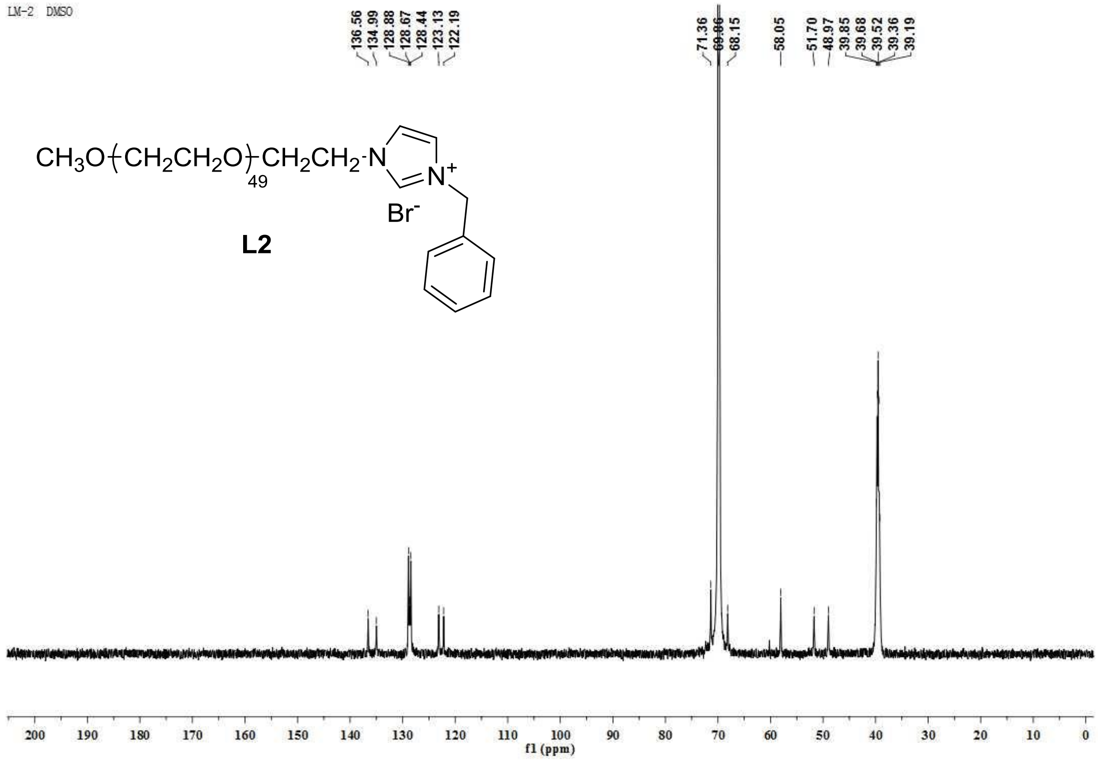
LM-2 DMSO
136.56
134.99
128.88
128.67
128.44
123.13
122.19
71.36
68.15
58.05
51.70
48.97
39.85
39.68
39.52
39.36
39.19
L2
Br-
f1 (ppm)

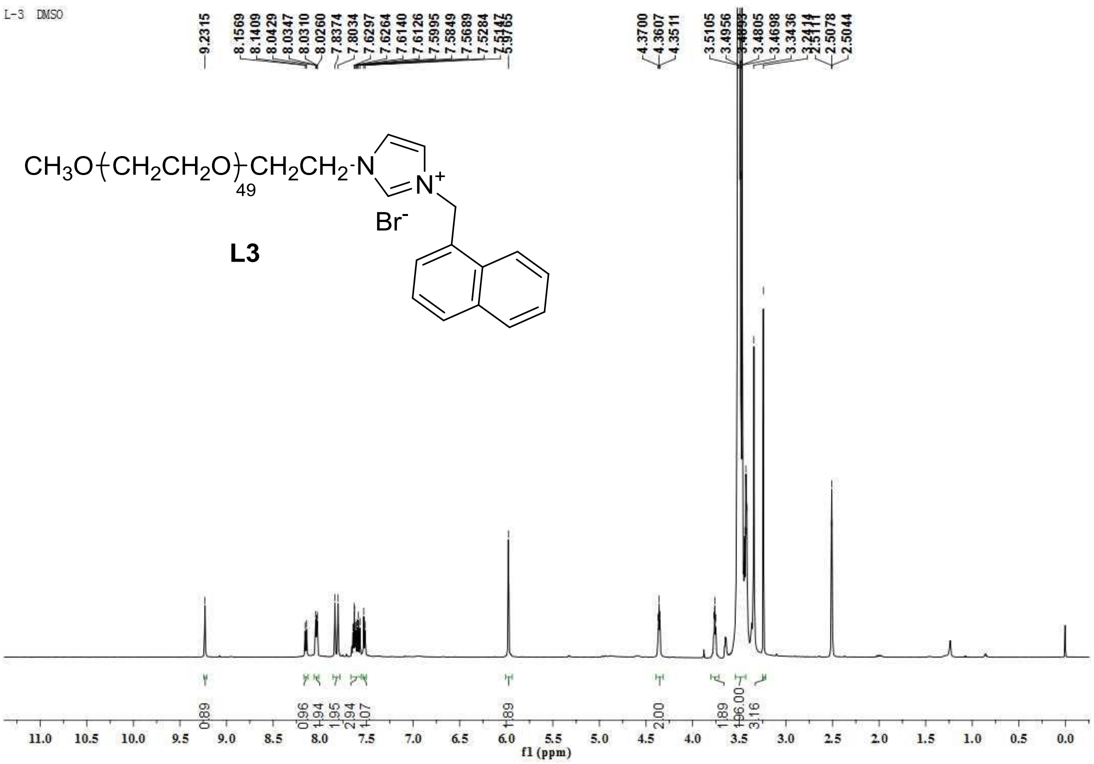
L-3 DMSO
CH3O(CH2CH2O)49CH2CH2-N
N+
Br-
L3
f1 (ppm)

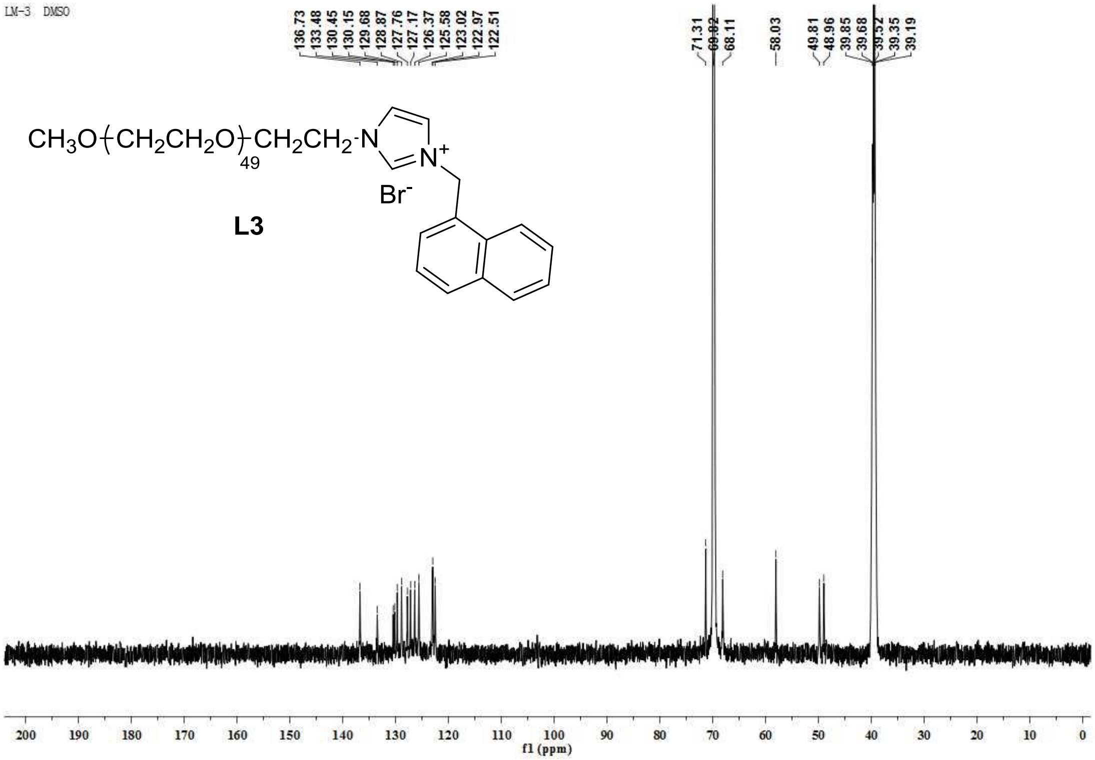
LM-3 DMSO
CH3O(CH2CH2O)49CH2CH2-N
N+
Br-
L3
f1 (ppm)

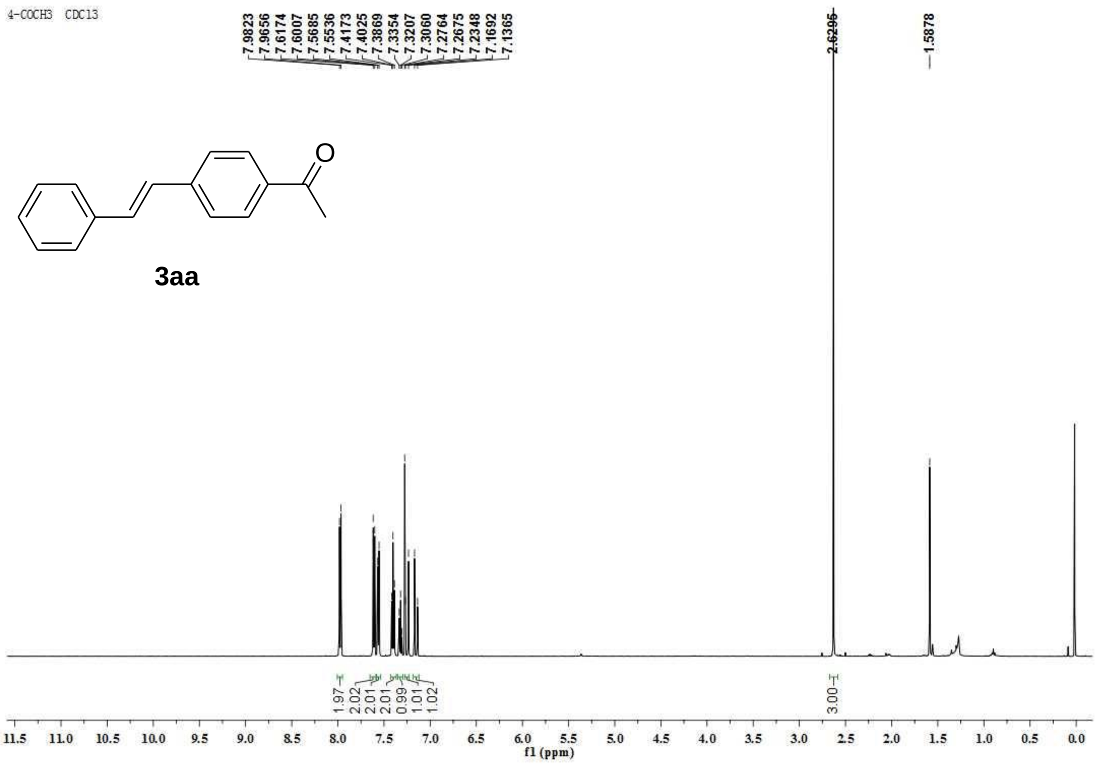
4-COCH3 CDCl3
7.9823
7.9656
7.6174
7.6007
7.5685
7.5536
7.4173
7.4025
7.3869
7.3354
7.3207
7.3060
7.2764
7.2675
7.2348
7.1692
7.1365
2.6296
1.5878
O
3aa
1.97
2.02
2.01
2.01
0.99
1.01
1.02
3.00
f1 (ppm)

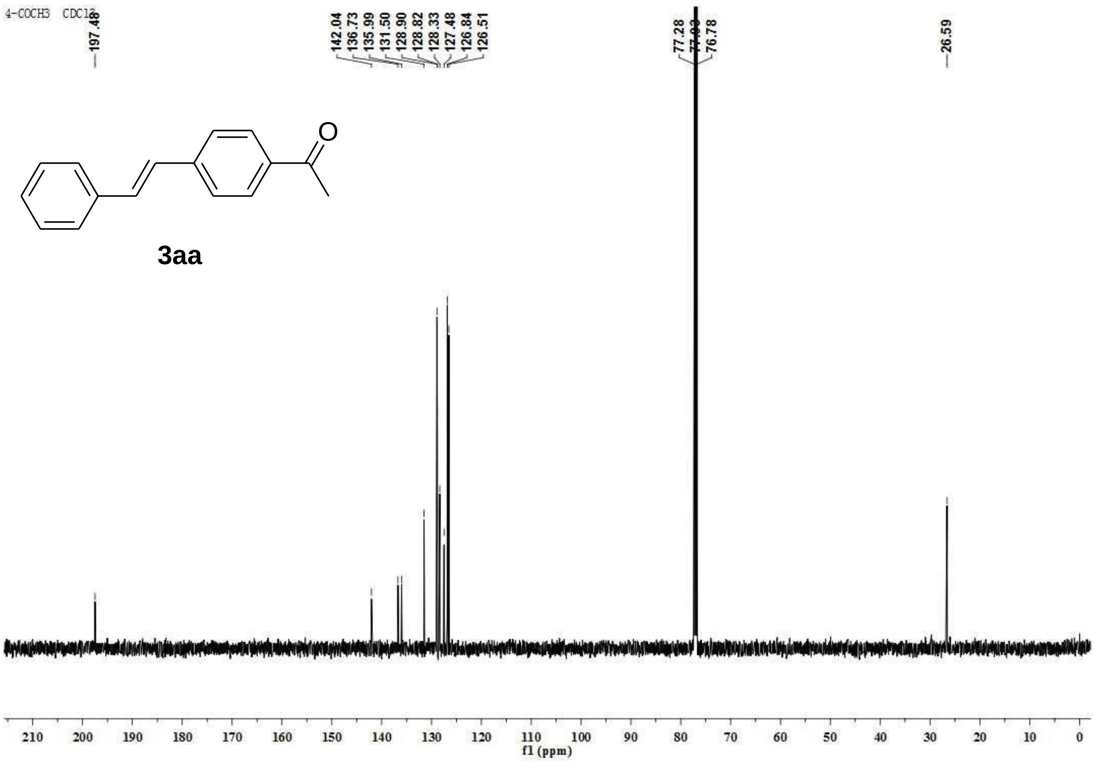
4-COCH3 CDCl3
197.48
142.04
136.73
135.99
131.50
128.90
128.82
128.33
127.48
126.84
126.51
77.28
76.78
26.59
O
3aa
f1 (ppm)

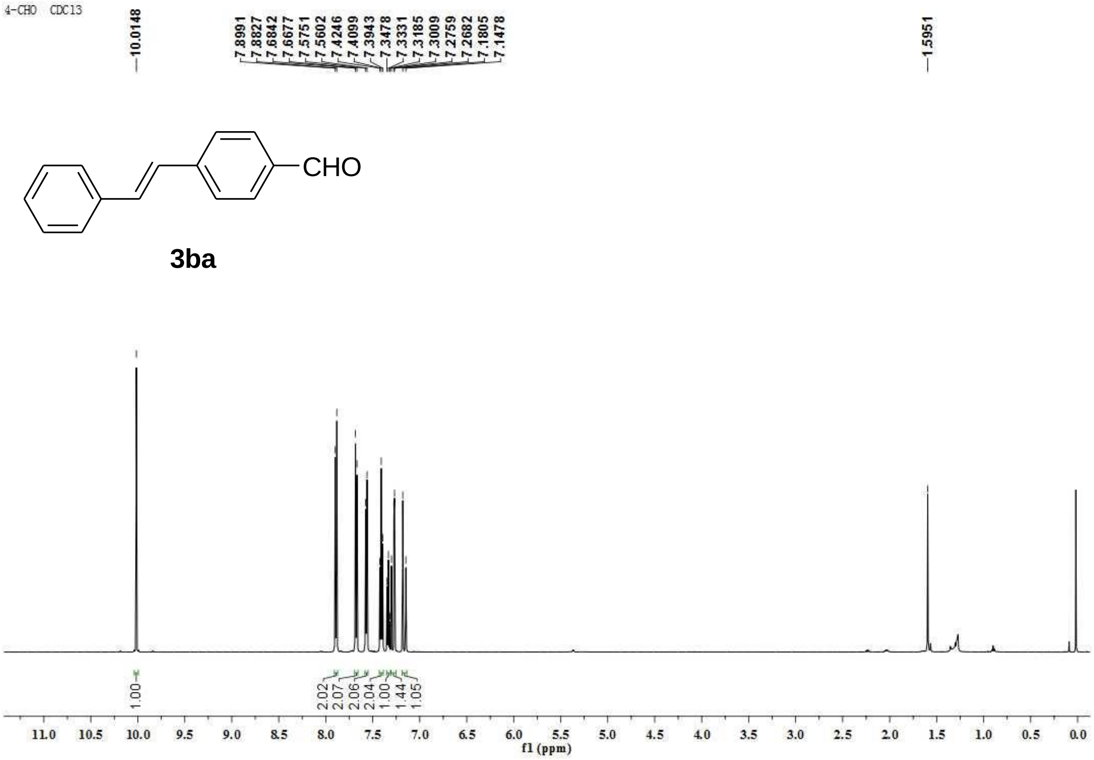
4-CHO CDCl3
10.0148
7.8991
7.8827
7.6842
7.6677
7.5751
7.5602
7.4246
7.4099
7.3943
7.3478
7.3331
7.3185
7.3009
7.2759
7.2682
7.1805
7.1478
1.5951
CHO
3ba
1.00
2.02
2.07
2.06
2.04
1.00
1.44
1.05
11.0 10.5 10.0 9.5 9.0 8.5 8.0 7.5 7.0 6.5 6.0 5.5 5.0 4.5 4.0 3.5 3.0 2.5 2.0 1.5 1.0 0.5 0.0
f1 (ppm)

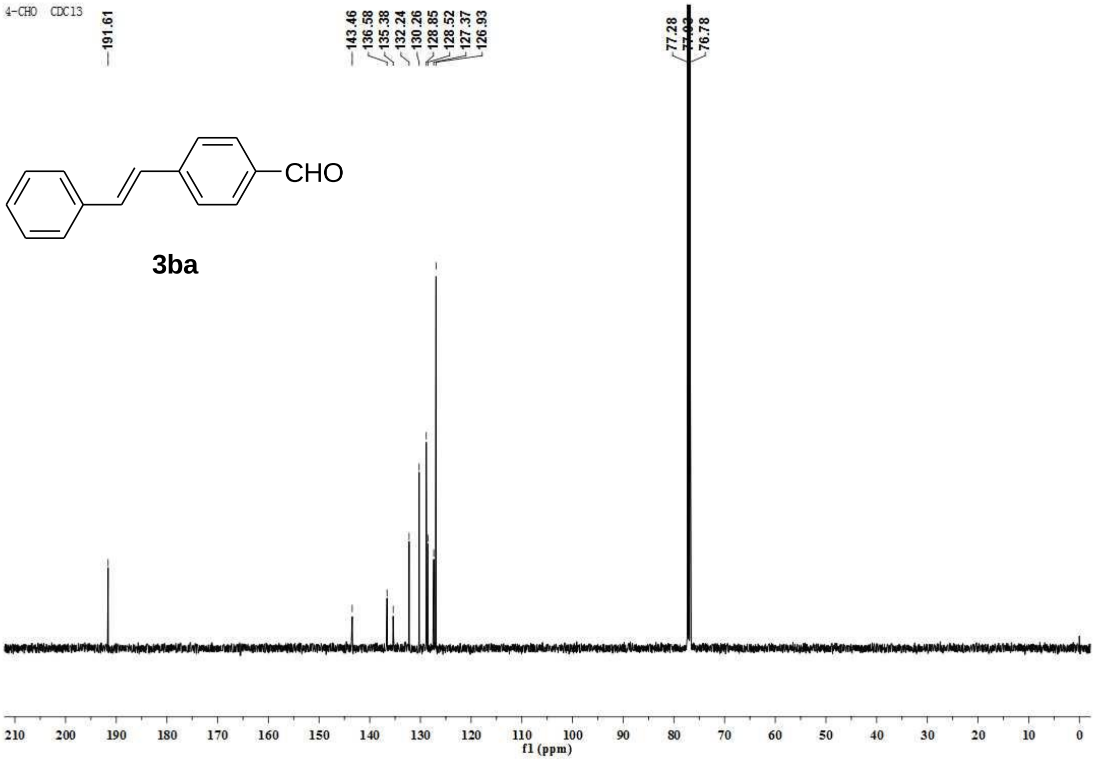
4-CHO CDCl3
191.61
143.46
136.58
135.38
132.24
130.26
128.85
128.52
127.37
126.93
77.28
76.78
CHO
3ba
210 200 190 180 170 160 150 140 130 120 110 100 90 80 70 60 50 40 30 20 10 0
f1 (ppm)

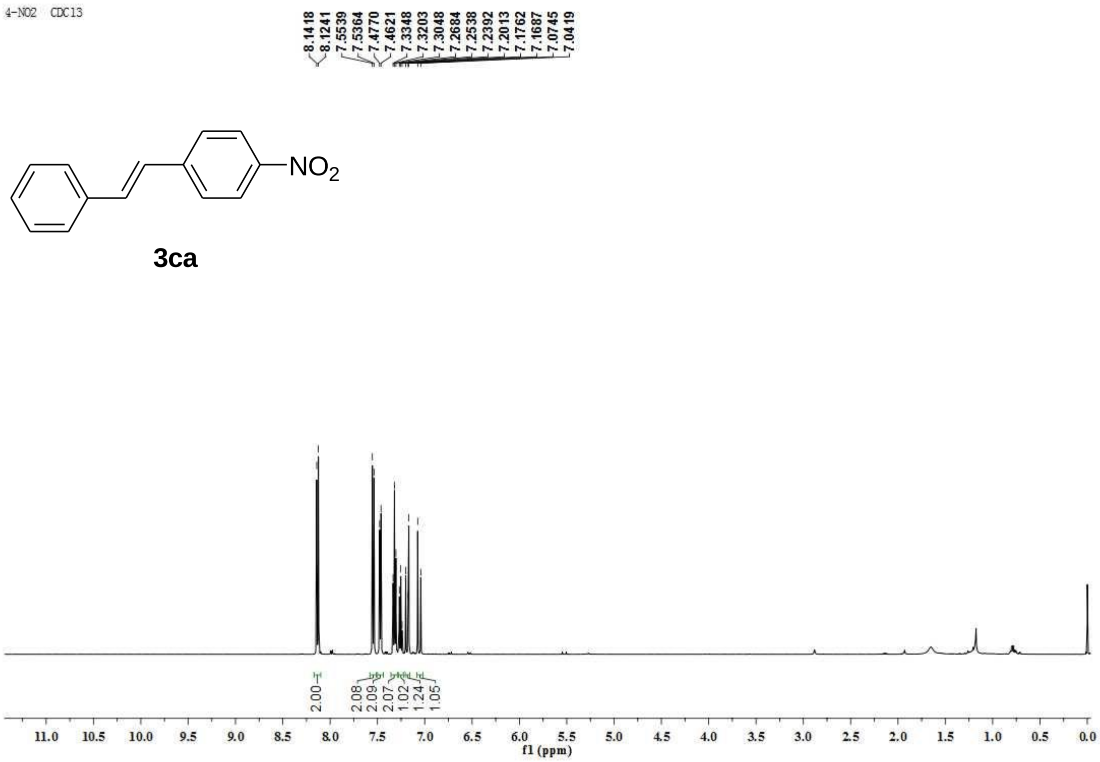
4-NO2 CDCl3
8.1418
8.1241
7.5539
7.5364
7.4770
7.4621
7.3348
7.3203
7.3048
7.2684
7.2538
7.2392
7.2013
7.1762
7.1687
7.0745
7.0419
NO2
3ca
2.00
2.08
2.09
2.07
1.02
1.24
1.05
11.0 10.5 10.0 9.5 9.0 8.5 8.0 7.5 7.0 6.5 6.0 5.5 5.0 4.5 4.0 3.5 3.0 2.5 2.0 1.5 1.0 0.5 0.0
f1 (ppm)

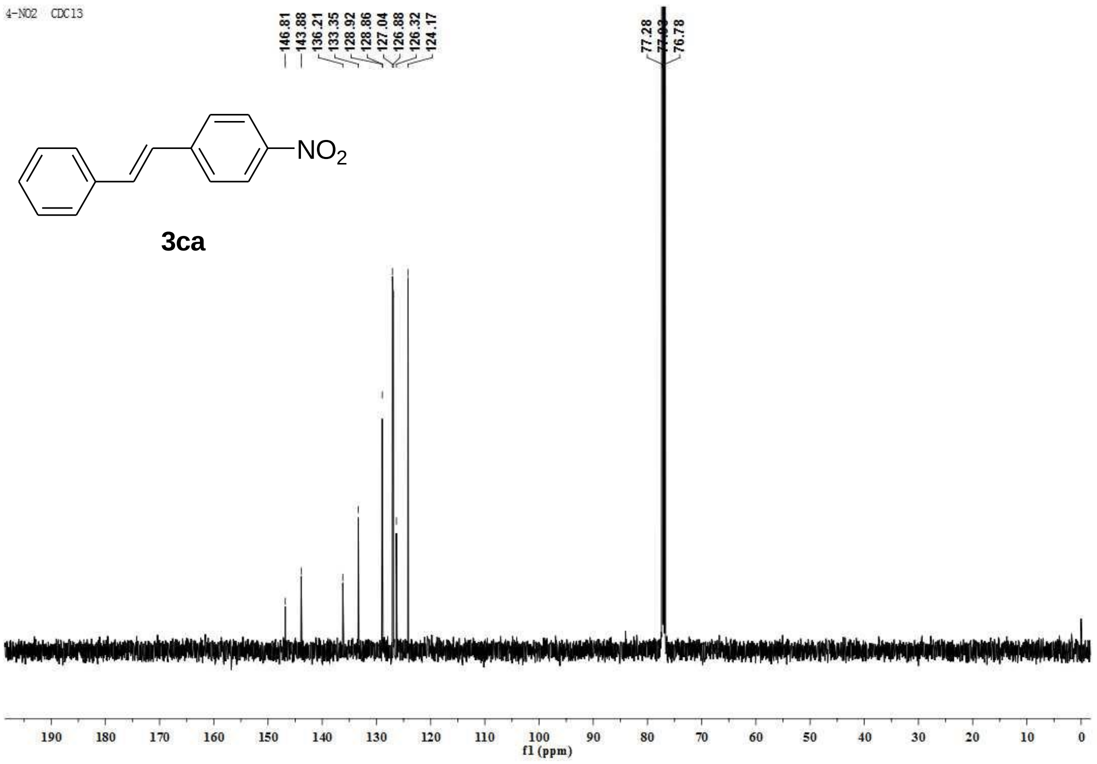
4-NO2 CDCl3
146.81
143.88
136.21
133.35
128.92
128.86
127.04
126.88
126.32
124.17
77.28
76.78
NO2
3ca
190 180 170 160 150 140 130 120 110 100 90 80 70 60 50 40 30 20 10 0
f1 (ppm)

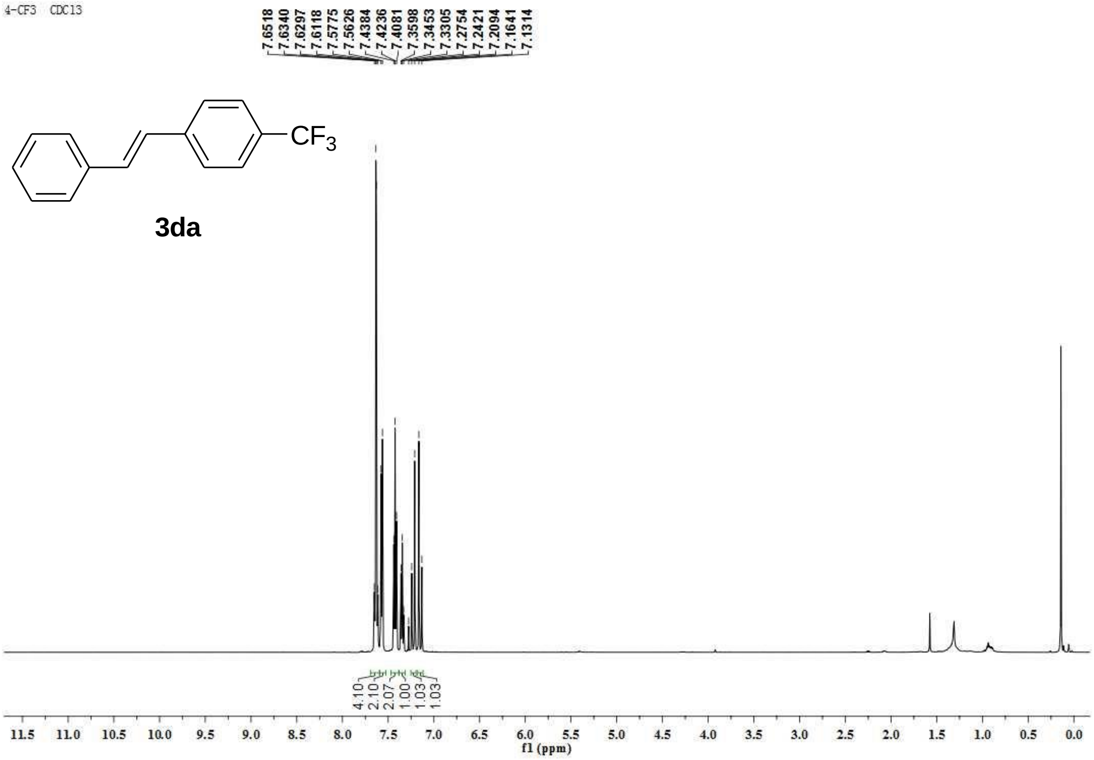
4-CF3 CDCl3
7.6518
7.6340
7.6297
7.6118
7.5775
7.5626
7.4384
7.4236
7.4081
7.3598
7.3453
7.3305
7.2754
7.2421
7.2094
7.1641
7.1314
CF3
3da
4.10
2.10
2.07
1.00
1.03
1.03
f1 (ppm)

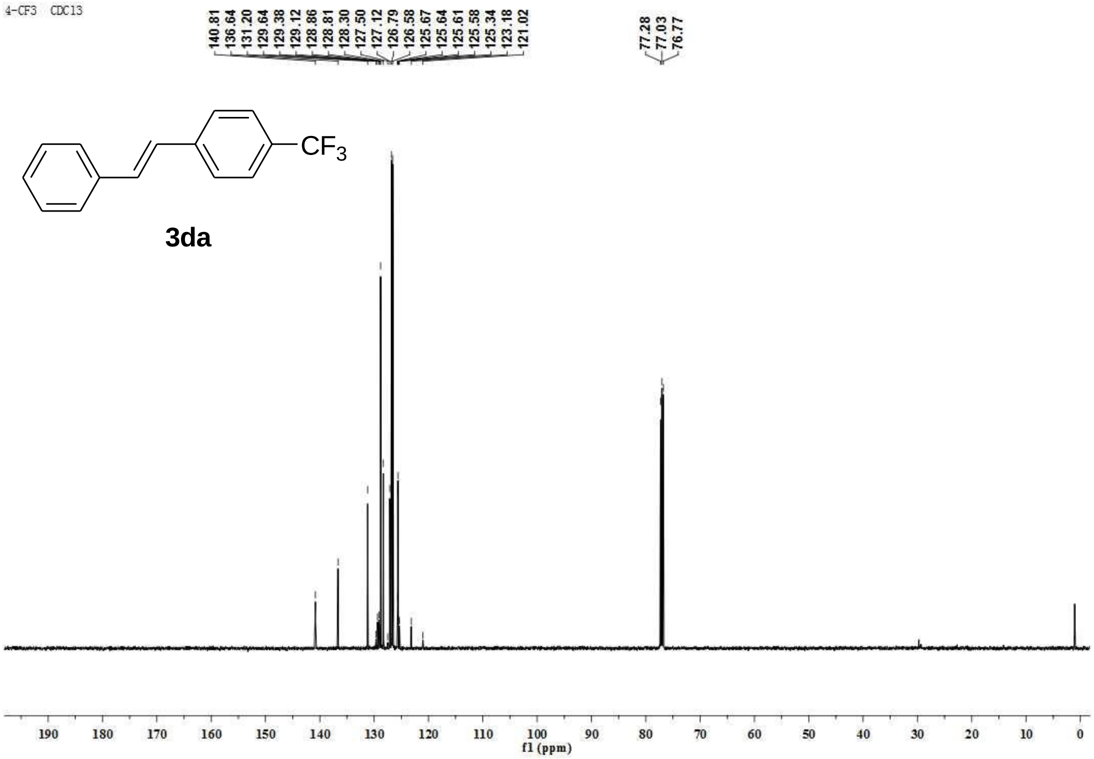
4-CF3 CDCl3
140.81
136.64
131.20
129.64
129.38
129.12
128.86
128.81
128.30
127.50
127.12
126.79
126.58
125.67
125.64
125.61
125.58
125.34
123.18
121.02
77.28
77.03
76.77
CF3
3da
f1 (ppm)

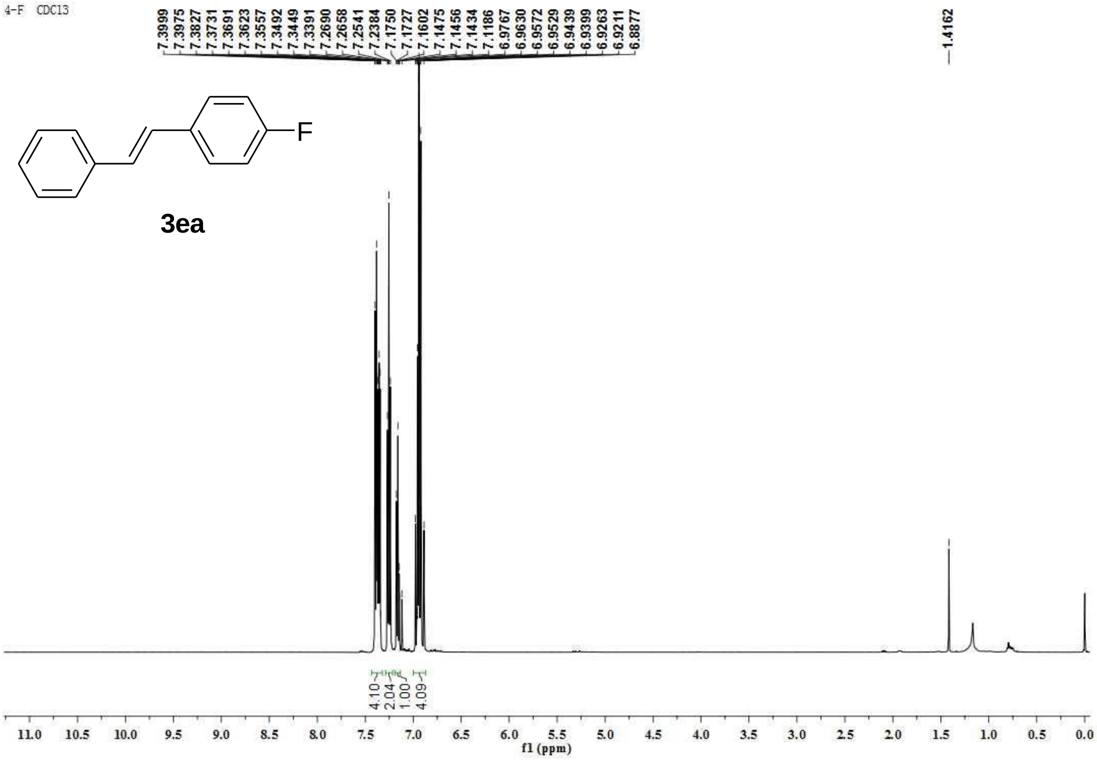
4-F CDCl3
7.3999
7.3975
7.3827
7.3731
7.3691
7.3623
7.3557
7.3492
7.3449
7.3391
7.2690
7.2658
7.2541
7.2384
7.1750
7.1727
7.1602
7.1475
7.1456
7.1434
7.1186
6.9767
6.9630
6.9572
6.9529
6.9439
6.9399
6.9263
6.9211
6.8877
1.4162
F
3ea
4.10
2.04
1.00
4.09
f1 (ppm)

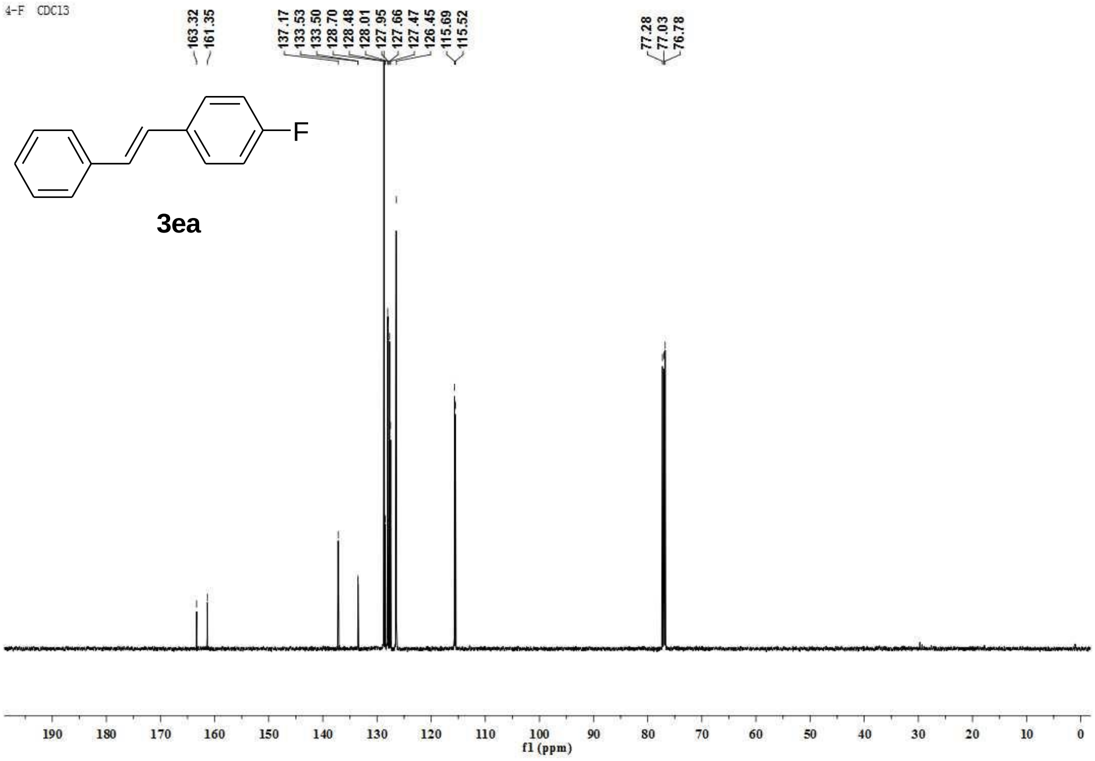
4-F CDCl3
163.32
161.35
137.17
133.53
133.50
128.70
128.48
128.01
127.95
127.66
127.47
126.45
115.69
115.52
77.28
77.03
76.78
F
3ea
f1 (ppm)

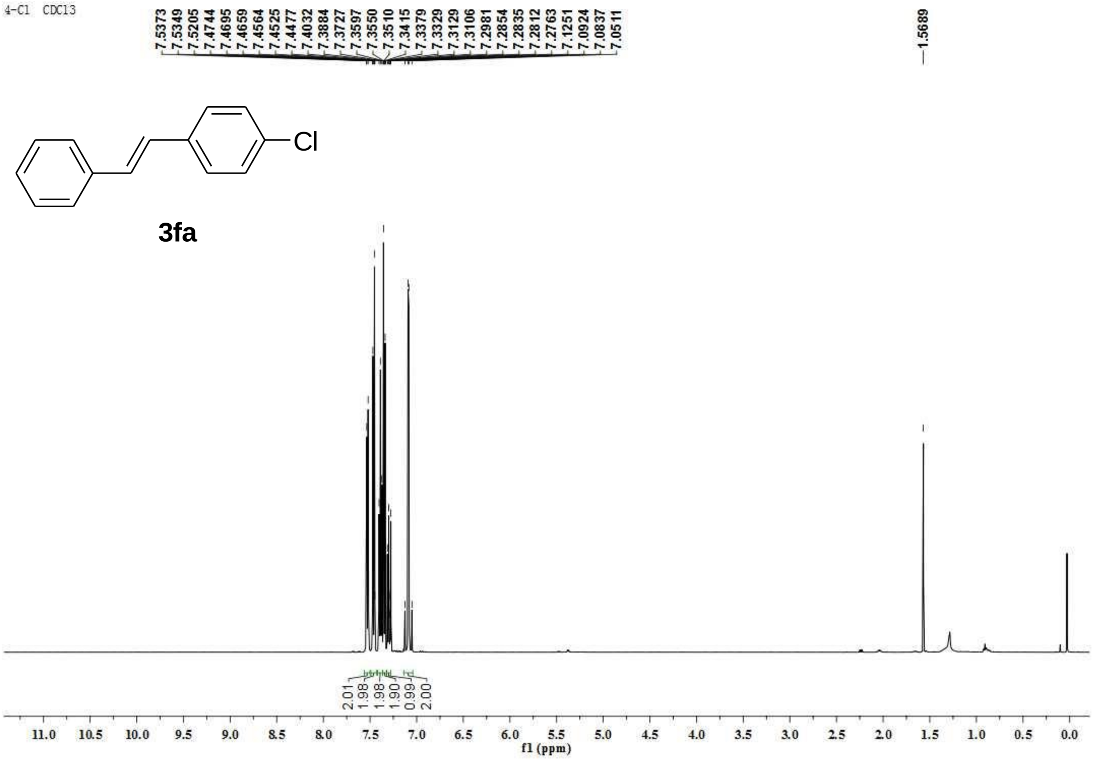
4-Cl CDCl3
7.5373
7.5349
7.5205
7.4744
7.4695
7.4659
7.4564
7.4525
7.4477
7.4032
7.3884
7.3727
7.3597
7.3550
7.3510
7.3415
7.3379
7.3329
7.3129
7.3106
7.2981
7.2854
7.2835
7.2812
7.2763
7.1251
7.0924
7.0837
7.0511
1.5689
Cl
3fa
2.01
1.98
1.98
1.90
0.99
2.00
f1 (ppm)

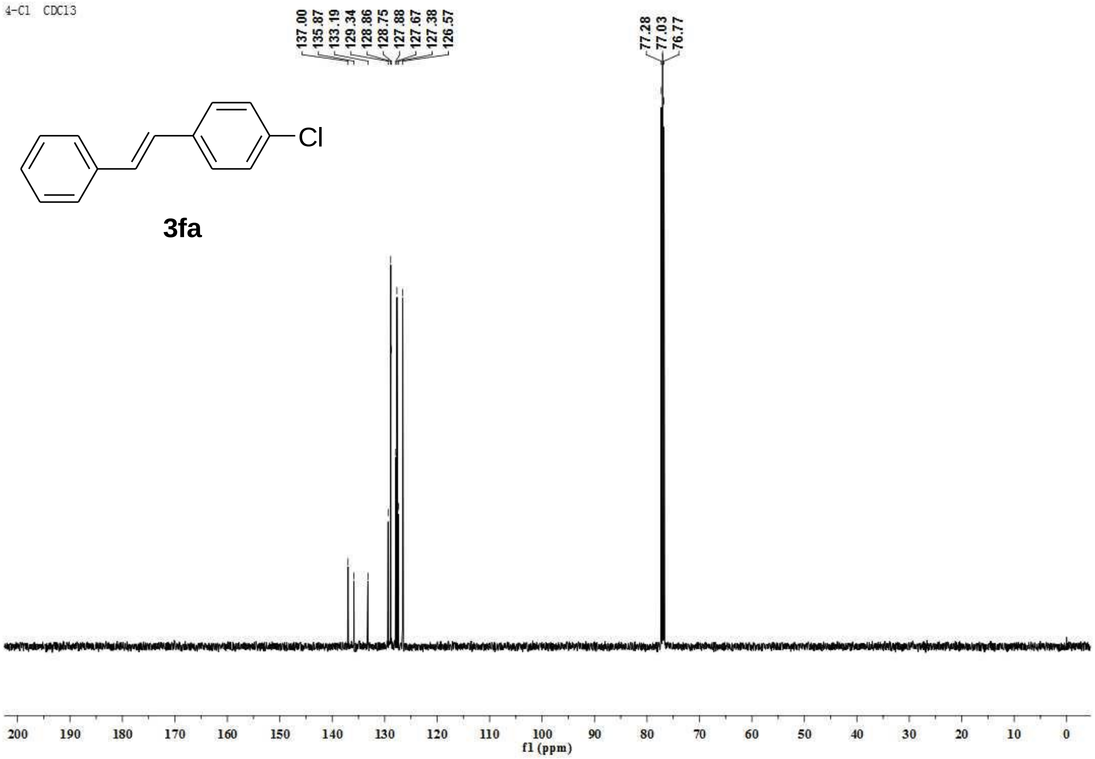
4-Cl CDCl3
137.00
135.87
133.19
129.34
128.86
128.75
127.88
127.67
127.38
126.57
77.28
77.03
76.77
Cl
3fa
f1 (ppm)

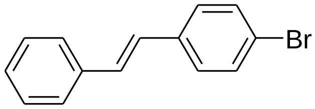

3ga

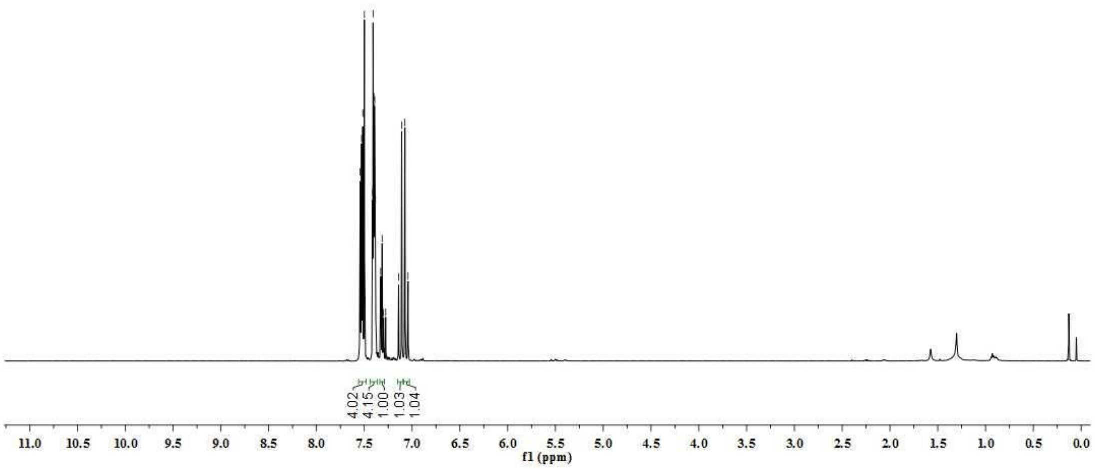
4-Br CDCl3
7.5432
7.5283
7.5213
7.5159
7.5127
7.5024
7.4989
7.4946
7.4131
7.4081
7.4041
7.3985
7.3958
7.3910
7.3829
7.3277
7.3131
7.2984
7.2756
7.1415
7.1088
7.0751
7.0424
4.02
4.15
1.00
1.03
1.04
f1 (ppm)

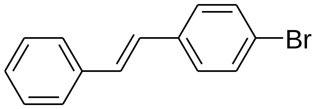

3ga

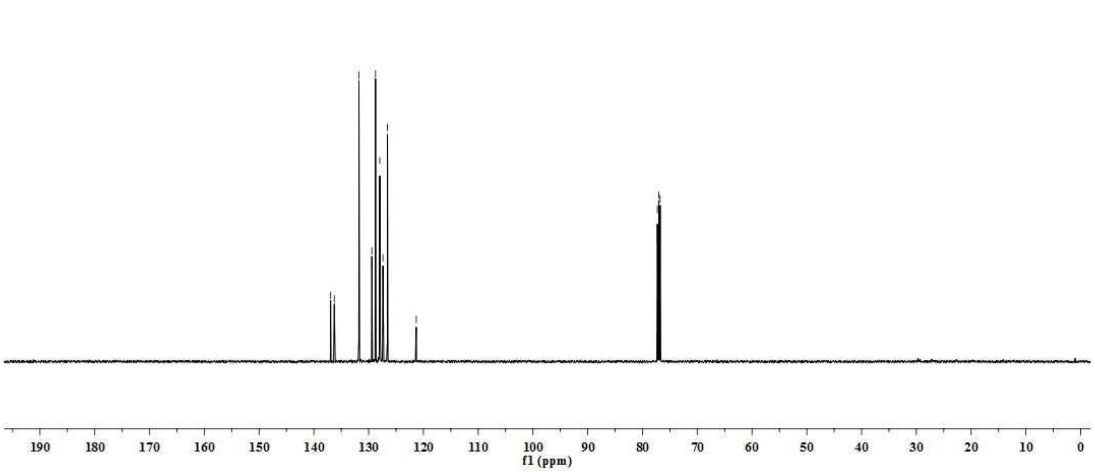
4-Br CDCl3
136.95
136.27
131.77
129.42
128.74
127.97
127.89
127.39
126.57
121.30
77.28
77.03
76.77
f1 (ppm)

CDCL3

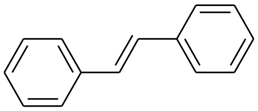

3ha

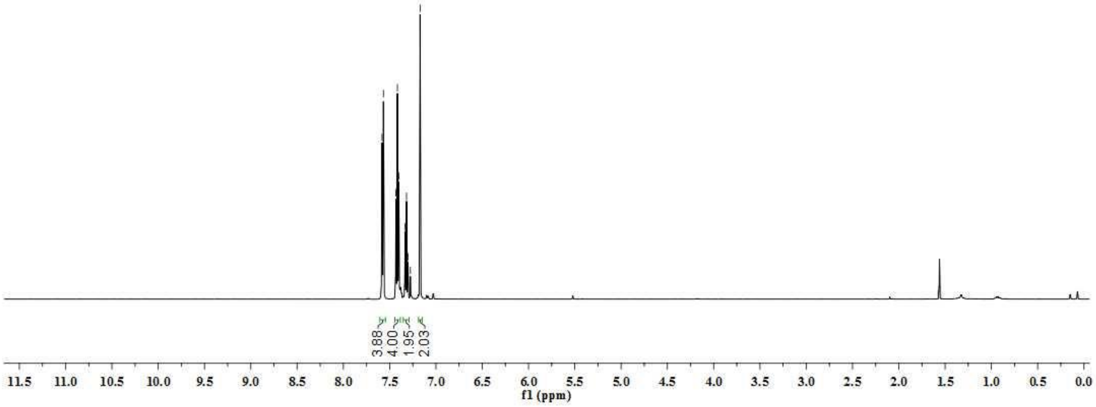

CDCL3

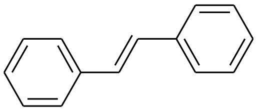

3ha

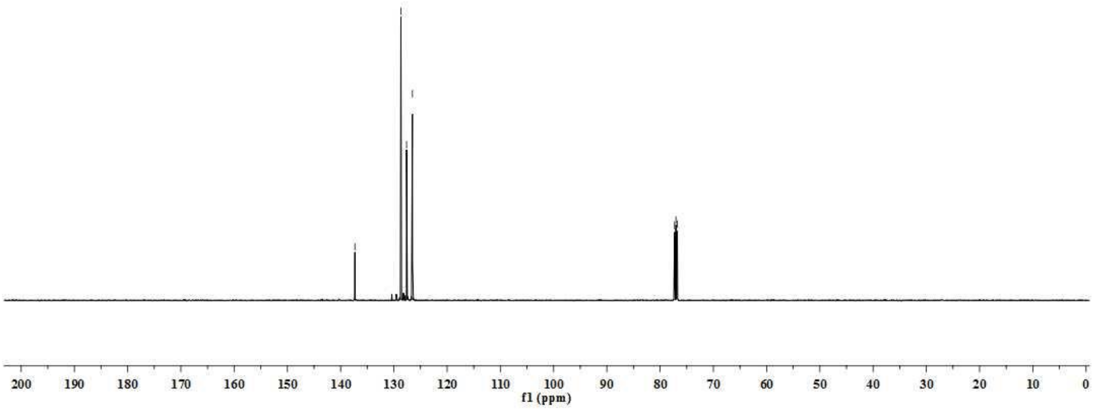

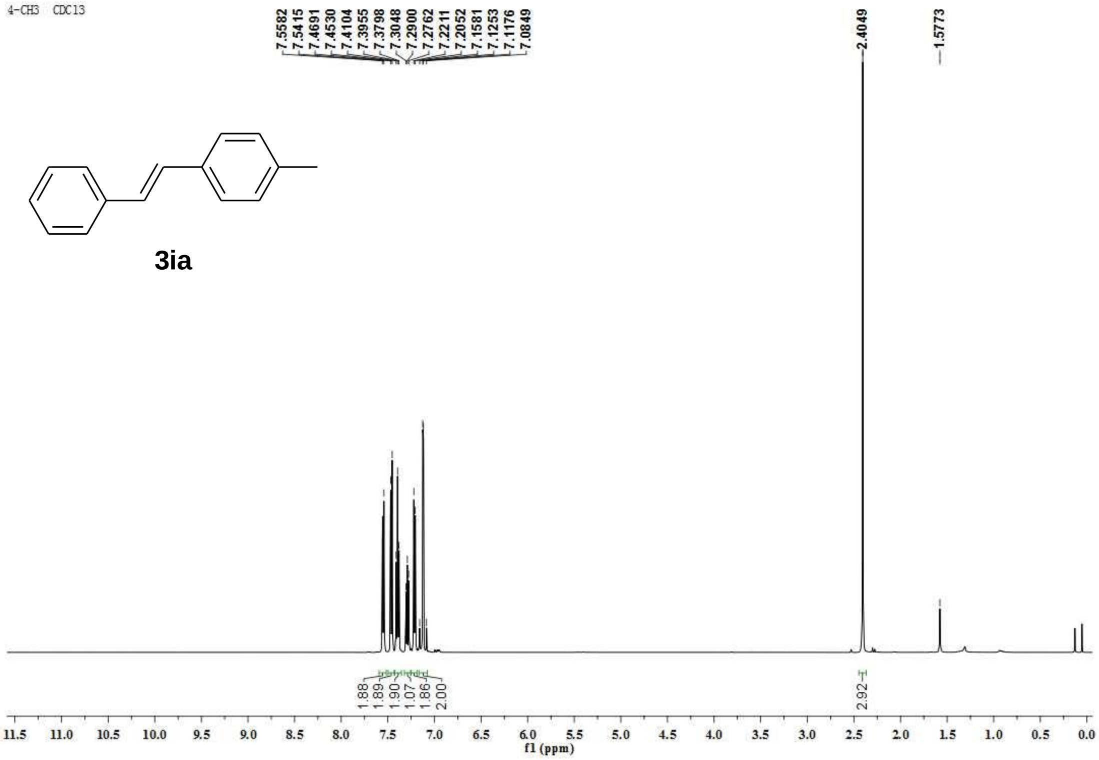
4-CH3 CDCl3
7.5582
7.5415
7.4691
7.4530
7.4104
7.3955
7.3798
7.3048
7.2900
7.2762
7.2211
7.2052
7.1581
7.1253
7.1176
7.0849
2.4049
1.5773
3ia
1.88
1.89
1.90
1.07
1.86
2.00
2.92
11.5 11.0 10.5 10.0 9.5 9.0 8.5 8.0 7.5 7.0 6.5 6.0 5.5 5.0 4.5 4.0 3.5 3.0 2.5 2.0 1.5 1.0 0.5 0.0
f1 (ppm)

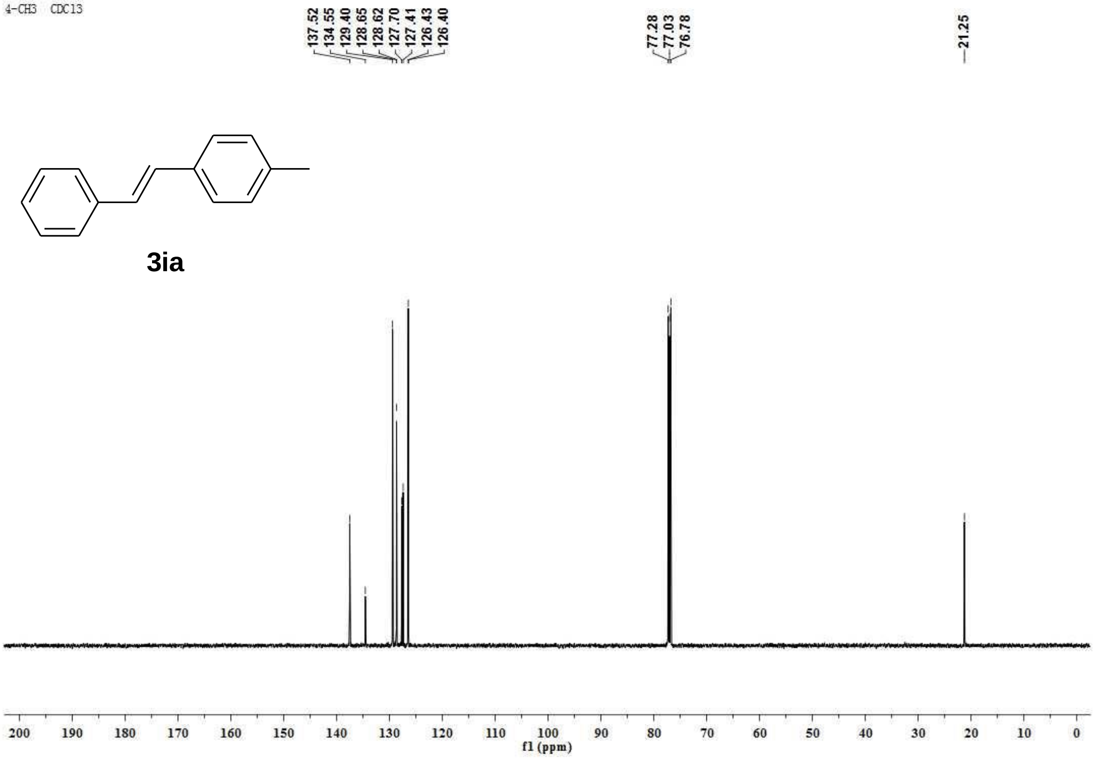
4-CH3 CDCl3
137.52
134.55
129.40
128.65
128.62
127.70
127.41
126.43
126.40
77.28
77.03
76.78
21.25
3ia
200 190 180 170 160 150 140 130 120 110 100 90 80 70 60 50 40 30 20 10 0
f1 (ppm)

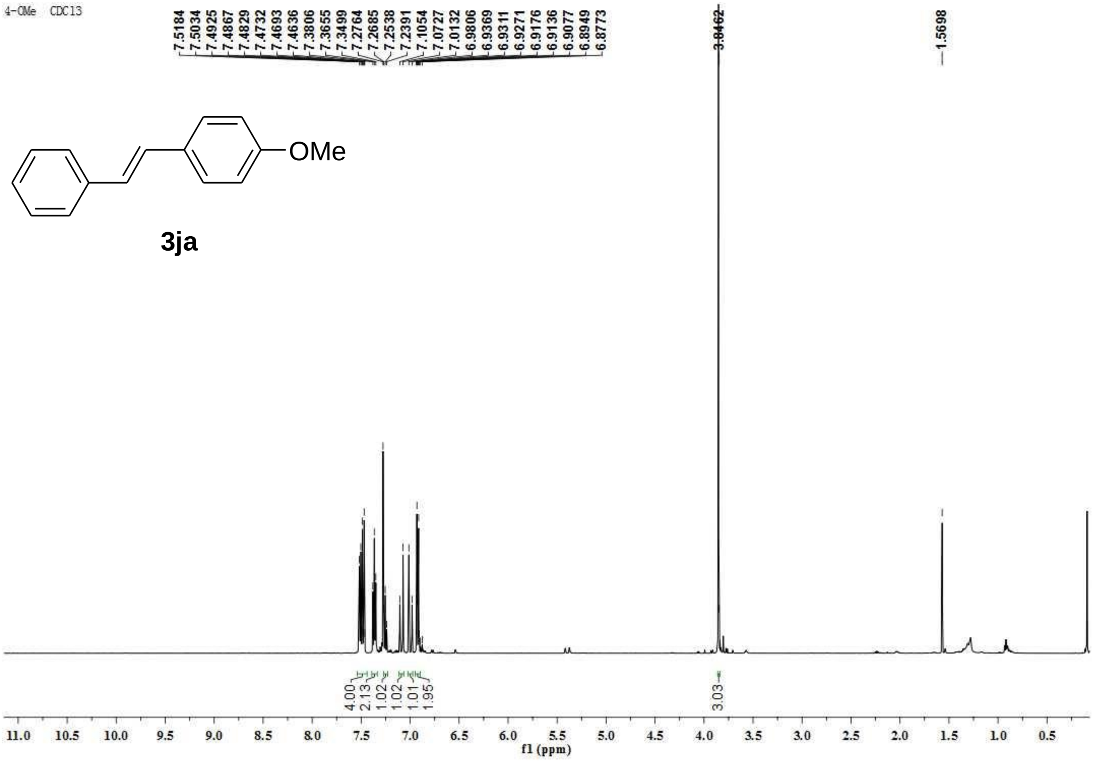
4-OMe CDCl3
7.5184
7.5034
7.4925
7.4867
7.4829
7.4732
7.4693
7.4636
7.3806
7.3655
7.3499
7.2764
7.2685
7.2538
7.2391
7.1054
7.0727
7.0132
6.9806
6.9369
6.9311
6.9271
6.9176
6.9136
6.9077
6.8949
6.8773
3.8462
1.5698
OMe
3ja
4.00
2.13
1.02
1.02
1.01
1.95
3.03
f1 (ppm)

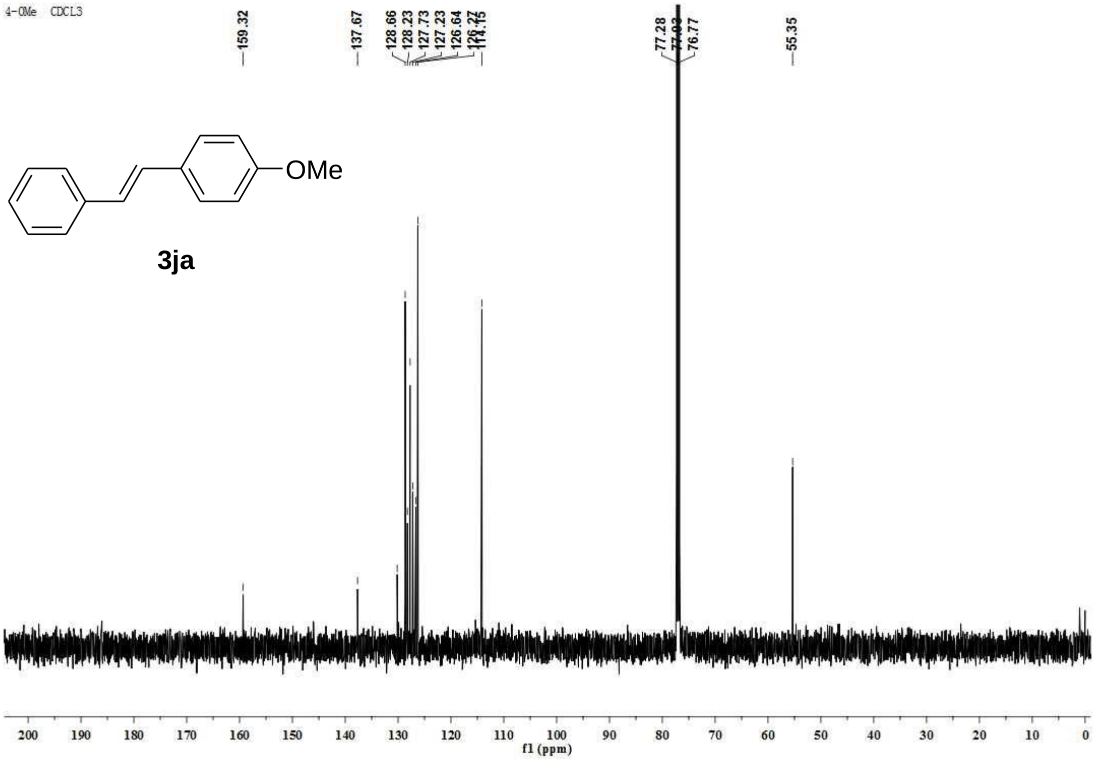
4-OMe CDCL3
159.32
137.67
128.66
128.23
127.73
127.23
126.64
126.27
77.28
77.02
76.77
55.35
OMe
3ja
f1 (ppm)

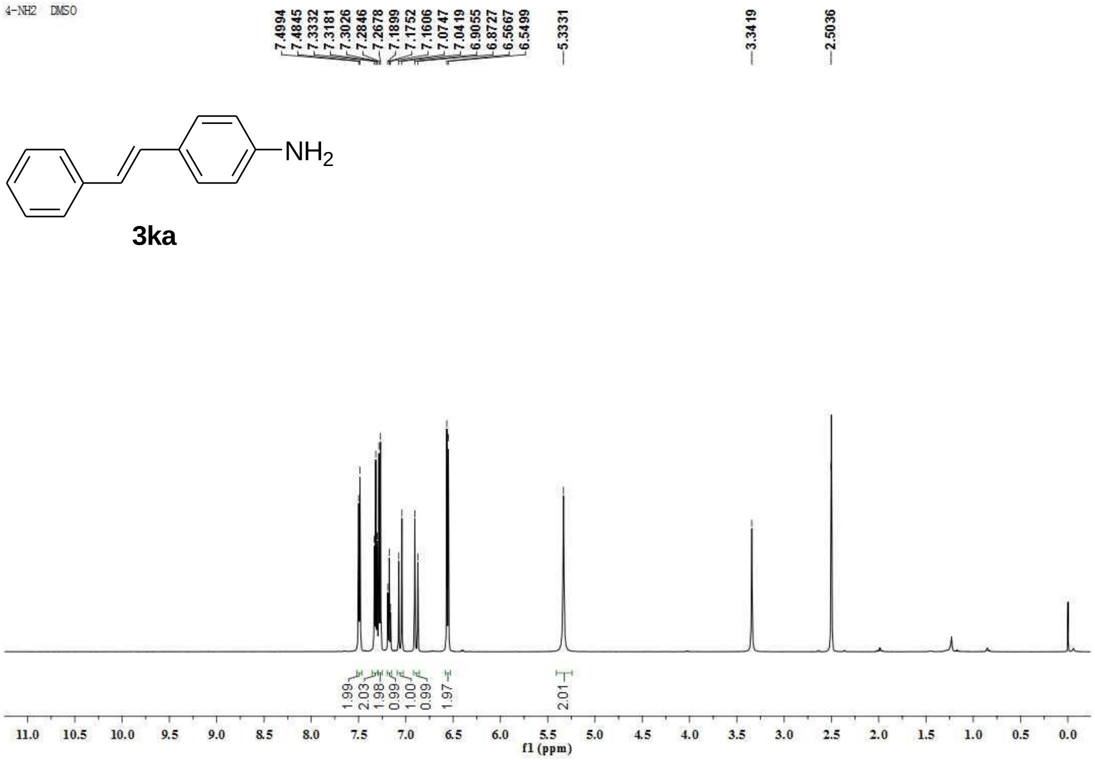
4-NH2 DMSO
7.4994
7.4845
7.3332
7.3181
7.3026
7.2846
7.2678
7.1899
7.1752
7.1606
7.0747
7.0419
6.9055
6.8727
6.5667
6.5499
5.3331
3.3419
2.5036
NH2
3ka
1.99
2.03
1.98
0.99
1.00
0.99
1.97
2.01
f1 (ppm)

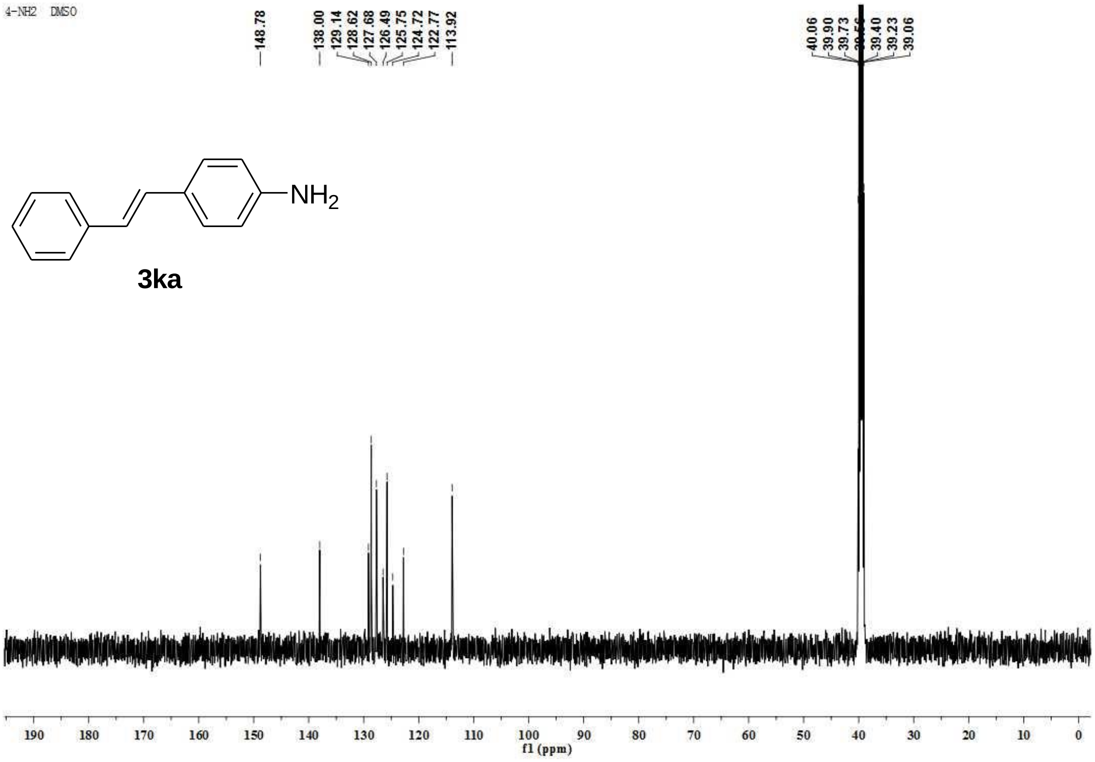
4-NH2 DMSO
148.78
138.00
129.14
128.62
127.68
126.49
125.75
124.72
122.77
113.92
40.06
39.90
39.73
39.40
39.23
39.06
NH2
3ka
f1 (ppm)

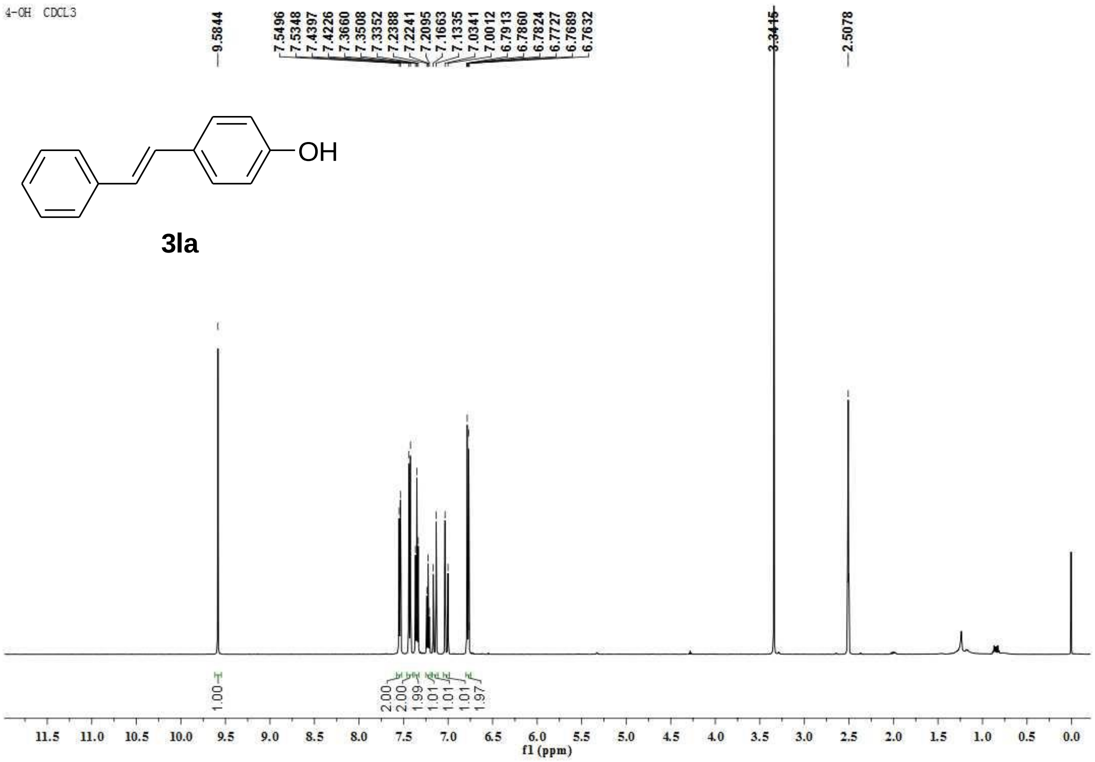
4-OH CDCL3
9.5844
7.5496
7.5348
7.4397
7.4226
7.3660
7.3508
7.3352
7.2388
7.2241
7.2095
7.1663
7.1335
7.0341
7.0012
6.7913
6.7860
6.7824
6.7727
6.7689
6.7632
2.5078
OH
3la
1.00
2.00
2.00
1.99
1.01
1.01
1.01
1.97
f1 (ppm)

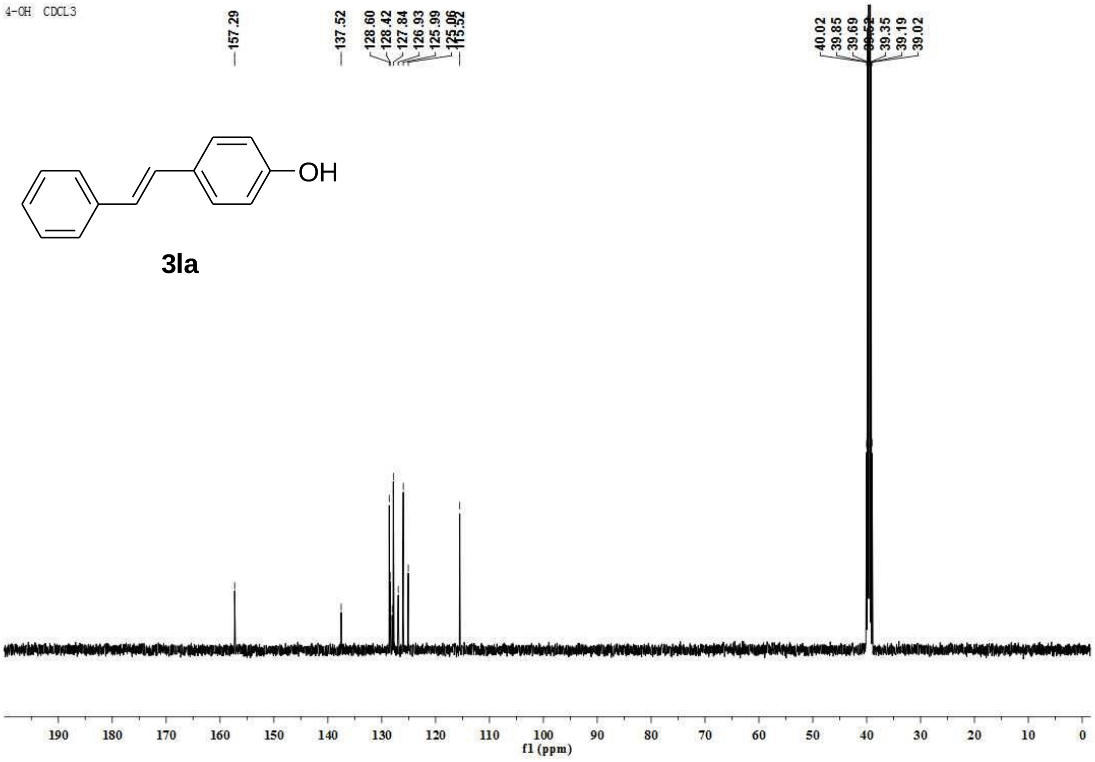
4-OH CDCL3
157.29
137.52
128.60
128.42
127.84
126.93
125.99
40.02
39.85
39.69
39.35
39.19
39.02
OH
3la
f1 (ppm)

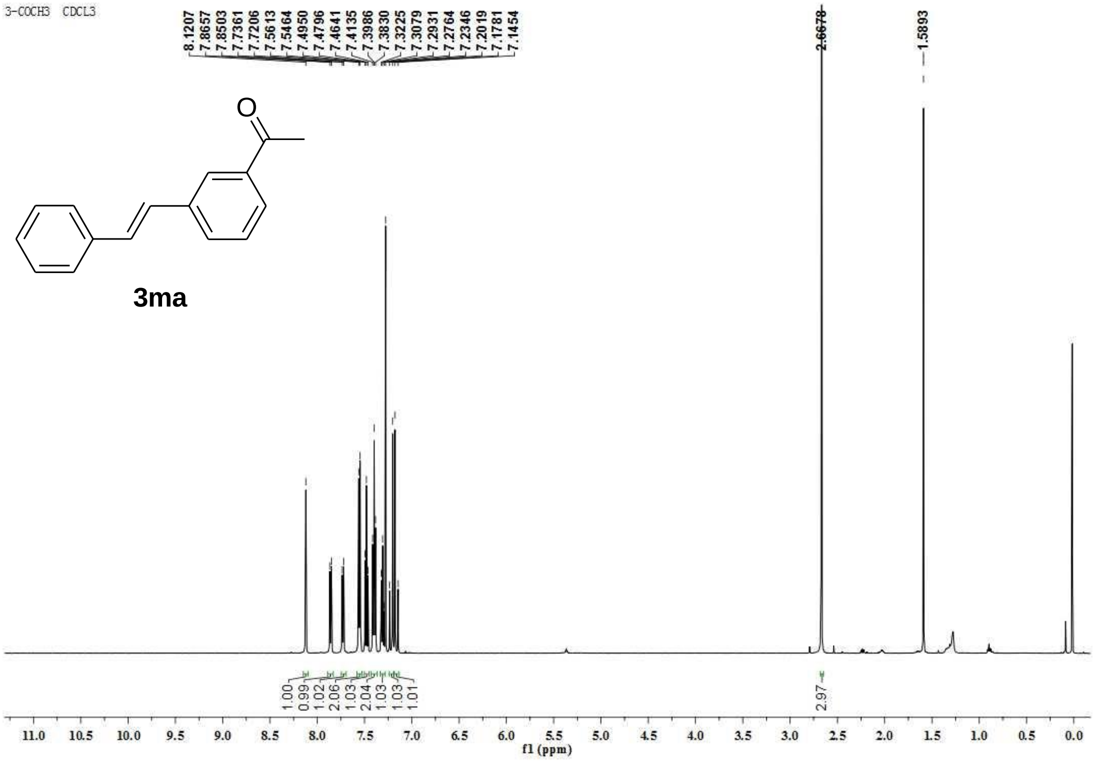
3-COCH3 CDCL3
3ma
f1 (ppm)

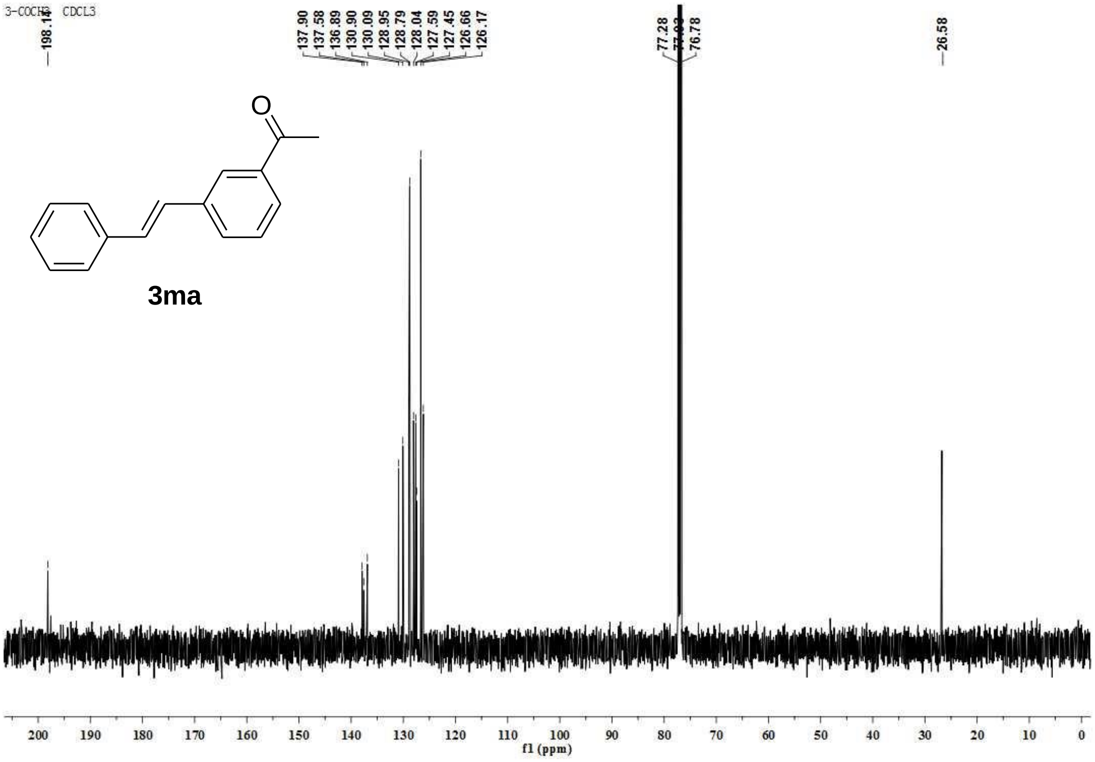
3-COCH3 CDCL3
3ma
f1 (ppm)

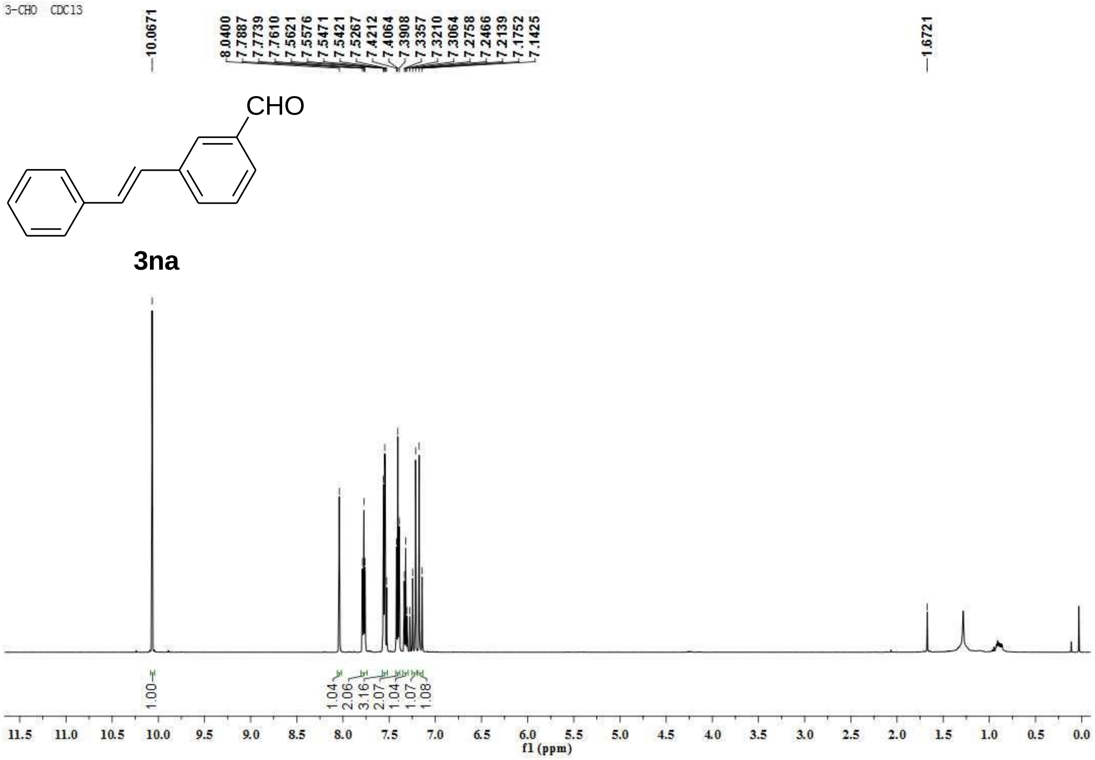
3-CHO CDCl3
10.0671
8.0400
7.7887
7.7739
7.7610
7.5621
7.5576
7.5471
7.5421
7.5267
7.4212
7.4064
7.3908
7.3357
7.3210
7.3064
7.2758
7.2466
7.2139
7.1752
7.1425
1.6721
CHO
1.00
1.04
2.06
3.16
2.07
1.04
1.07
1.08
f1 (ppm)

3na

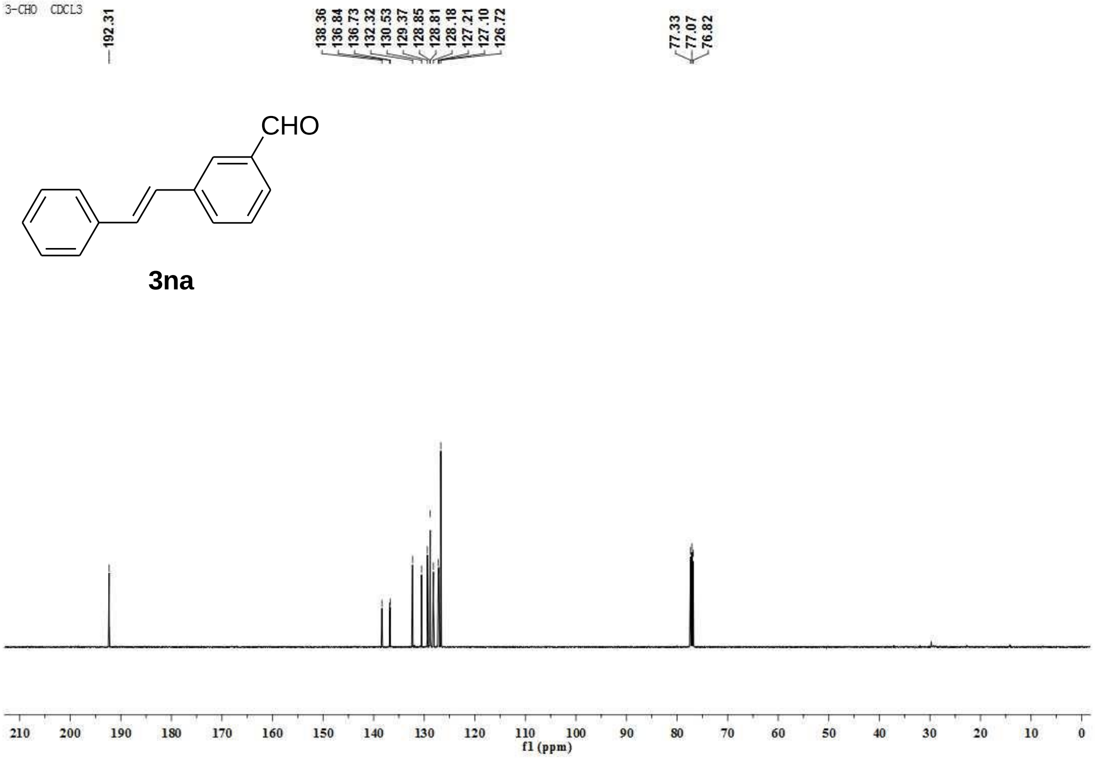
3-CHO CDCL3
192.31
138.36
136.84
136.73
132.32
130.53
129.37
128.85
128.81
128.18
127.21
127.10
126.72
77.33
77.07
76.82
CHO
f1 (ppm)

3na

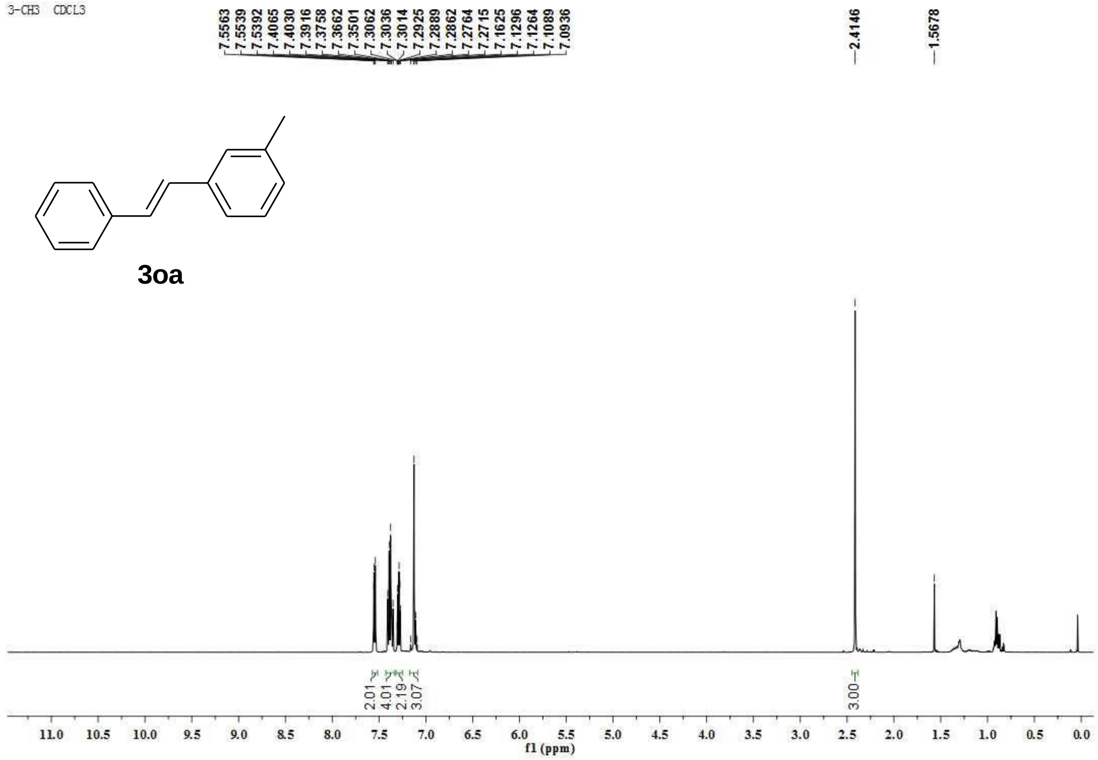
3-CH3 CDCL3
7.5563
7.5539
7.5392
7.4065
7.4030
7.3916
7.3758
7.3662
7.3501
7.3062
7.3036
7.3014
7.2925
7.2889
7.2862
7.2764
7.2715
7.1625
7.1296
7.1264
7.1089
7.0936
2.4146
1.5678
3oa
2.01
4.01
2.19
3.07
3.00
f1 (ppm)

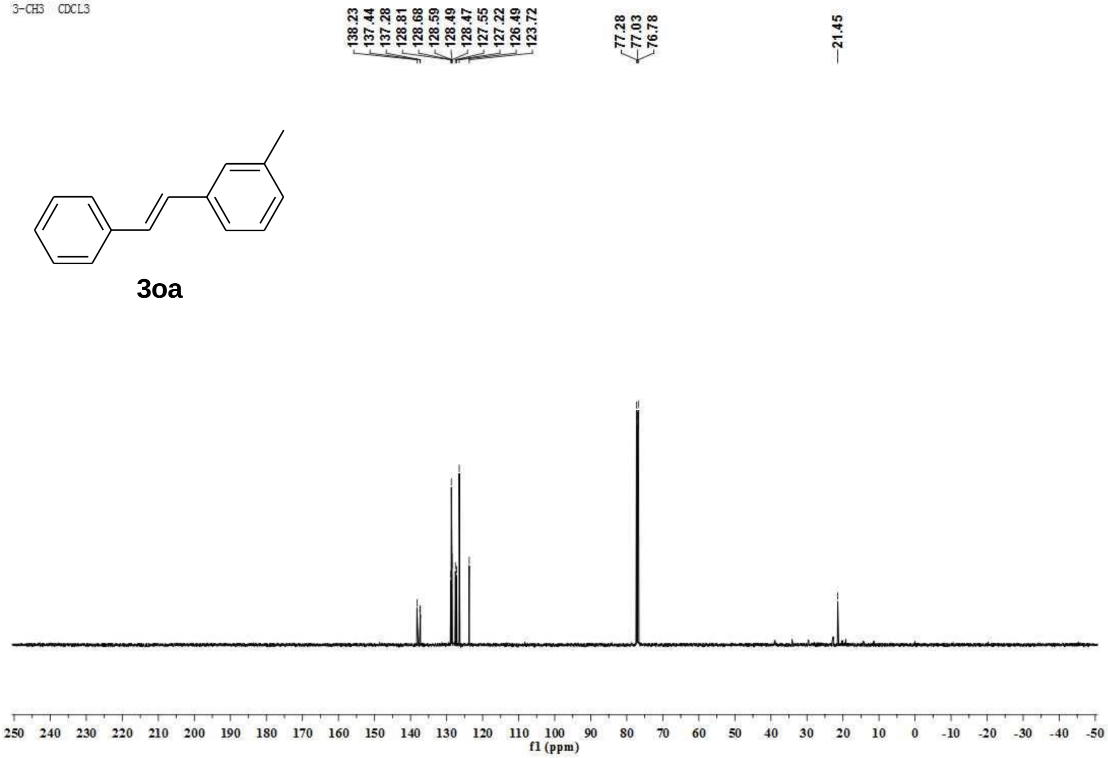
3-CH3 CDCL3
138.23
137.44
137.28
128.81
128.68
128.59
128.49
128.47
127.55
127.22
126.49
123.72
77.28
77.03
76.78
21.45
3oa
f1 (ppm)

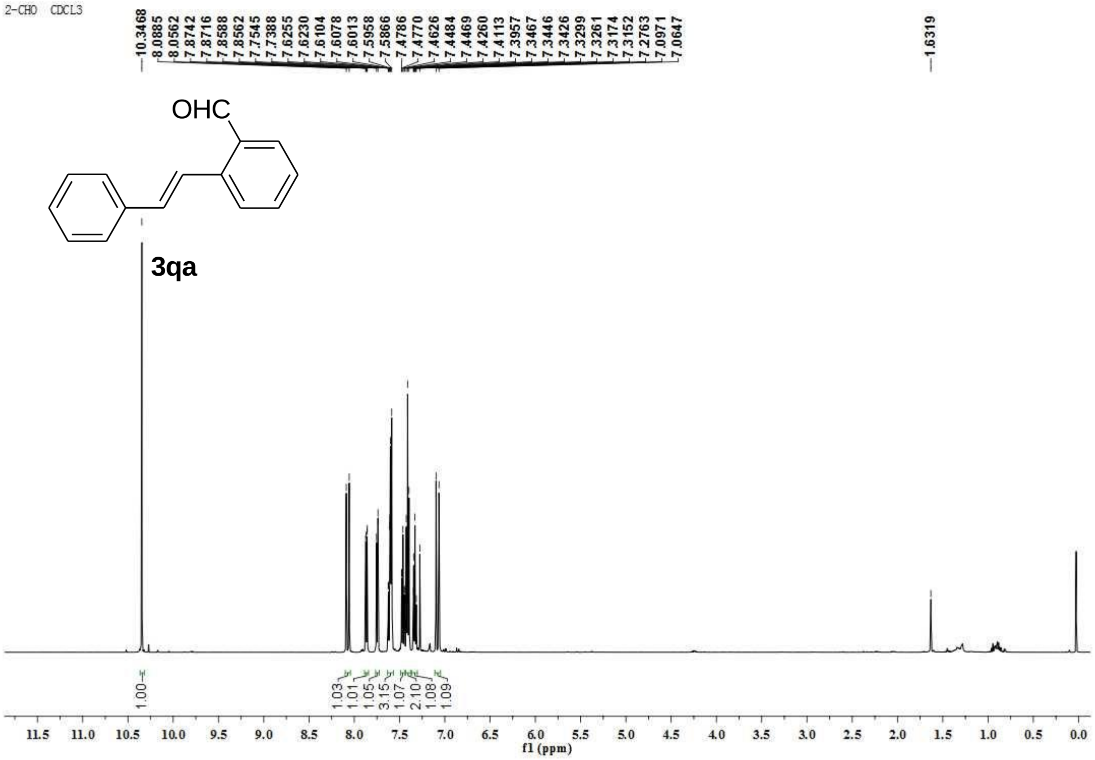
2-CHO CDCL3
10.3468
8.0885
8.0562
7.8742
7.8716
7.8588
7.8562
7.7545
7.7388
7.6255
7.6230
7.6104
7.6078
7.6013
7.5958
7.5866
7.4786
7.4770
7.4626
7.4484
7.4469
7.4260
7.4113
7.3957
7.3467
7.3446
7.3426
7.3299
7.3261
7.3174
7.3152
7.2763
7.0971
7.0647
1.6319
OHC
3qa
1.00
1.03
1.01
1.05
3.15
1.07
2.10
1.08
1.09
f1 (ppm)

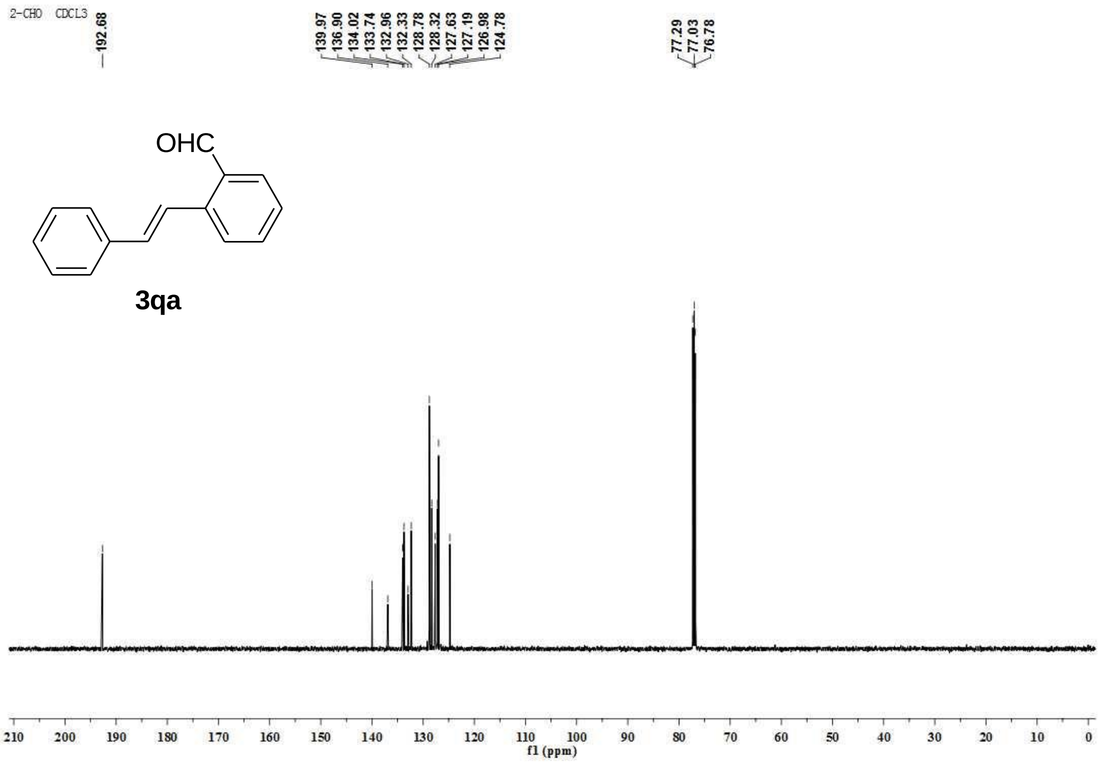
2-CHO CDCL3
192.68
139.97
136.90
134.02
133.74
132.96
132.33
128.78
128.32
127.63
127.19
126.98
124.78
77.29
77.03
76.78
OHC
3qa
f1 (ppm)

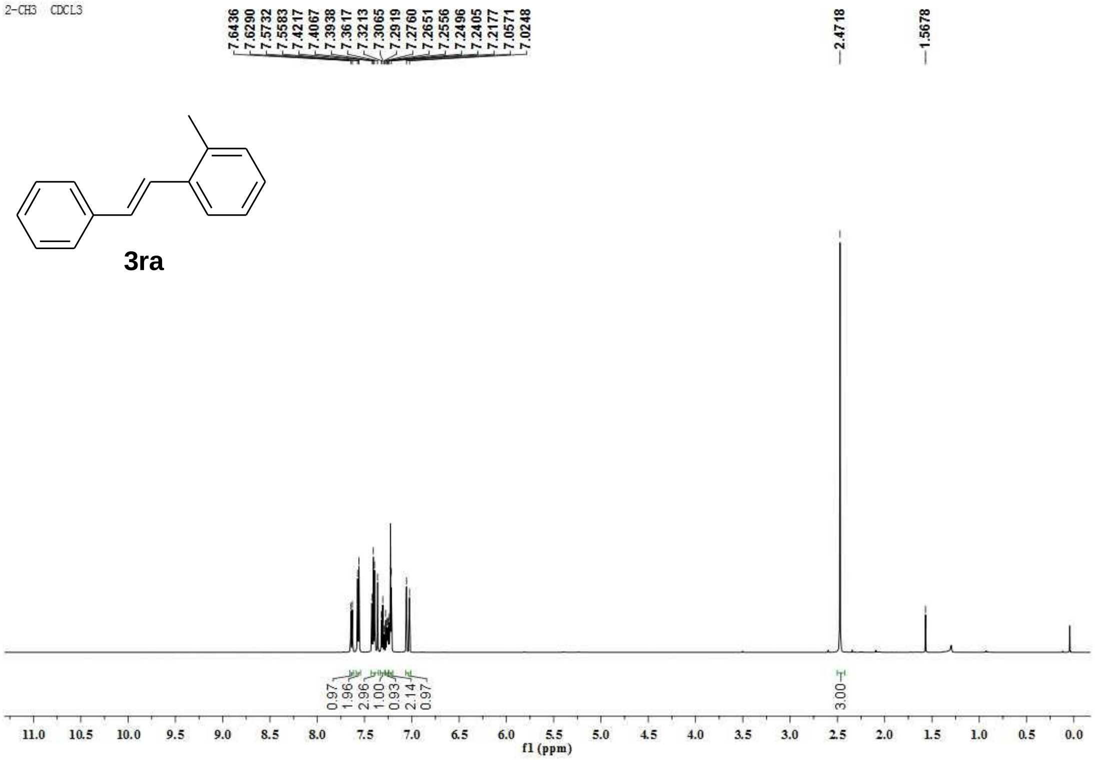
2-CH3 CDCL3
7.6436
7.6290
7.5732
7.5583
7.4217
7.4067
7.3938
7.3617
7.3213
7.3065
7.2919
7.2760
7.2651
7.2556
7.2496
7.2405
7.2177
7.0571
7.0248
2.4718
1.5678
3ra
0.97
1.96
2.96
1.00
0.93
2.14
0.97
3.00
11.0 10.5 10.0 9.5 9.0 8.5 8.0 7.5 7.0 6.5 6.0 5.5 5.0 4.5 4.0 3.5 3.0 2.5 2.0 1.5 1.0 0.5 0.0
f1 (ppm)

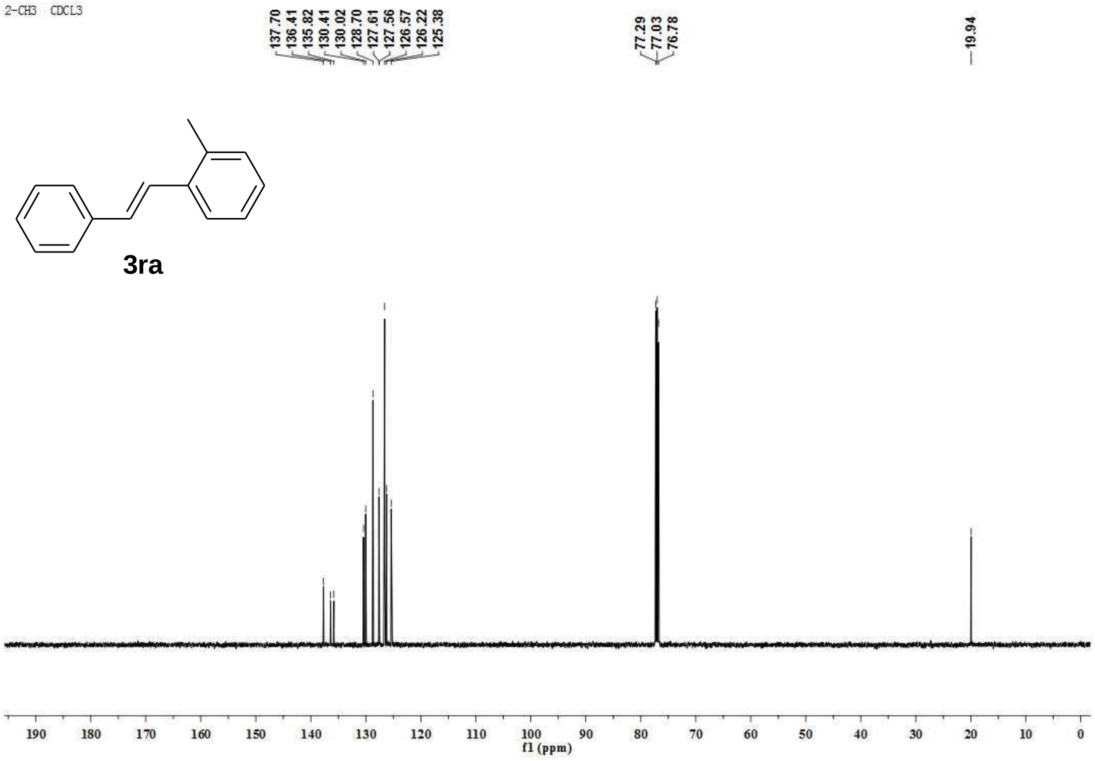
2-CH3 CDCL3
137.70
136.41
135.82
130.41
130.02
128.70
127.61
127.56
126.57
126.22
125.38
77.29
77.03
76.78
19.94
3ra
190 180 170 160 150 140 130 120 110 100 90 80 70 60 50 40 30 20 10 0
f1 (ppm)

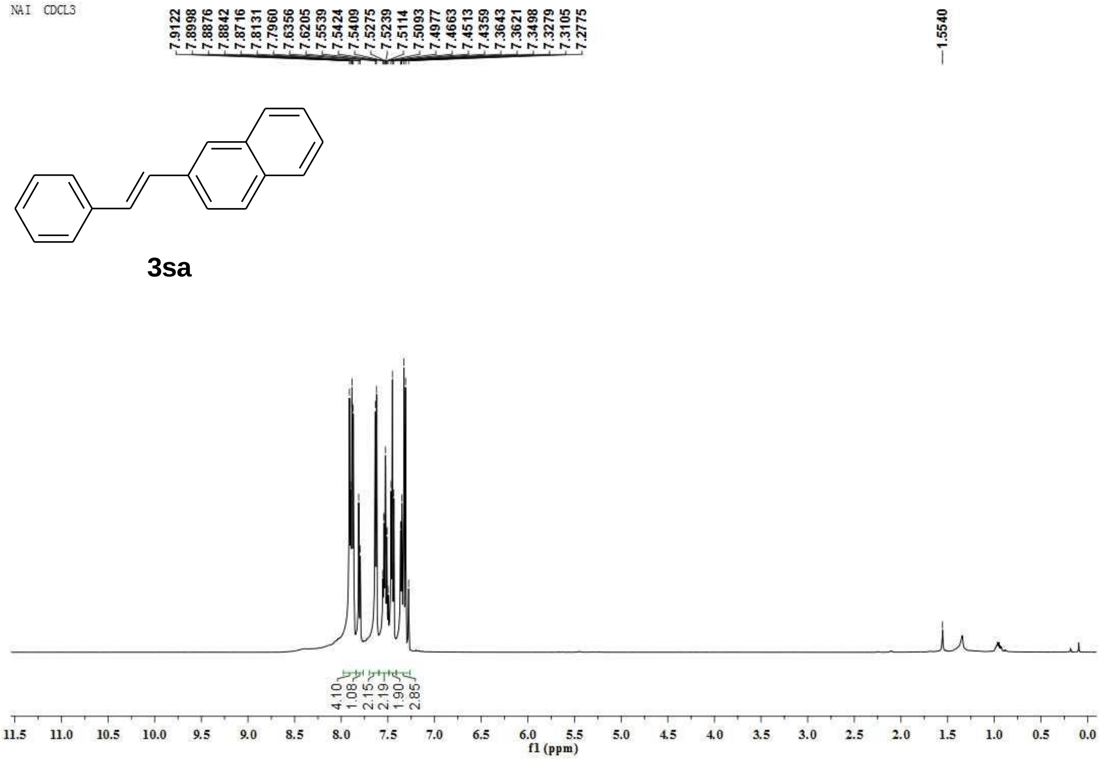
NAI CDCL3
7.9122
7.8998
7.8876
7.8842
7.8716
7.8131
7.7960
7.6356
7.6205
7.5539
7.5424
7.5409
7.5275
7.5239
7.5114
7.5093
7.4977
7.4663
7.4513
7.4359
7.3643
7.3621
7.3498
7.3279
7.3105
7.2775
1.5540
3sa
4.10
1.08
2.15
2.19
1.90
2.85
11.5 11.0 10.5 10.0 9.5 9.0 8.5 8.0 7.5 7.0 6.5 6.0 5.5 5.0 4.5 4.0 3.5 3.0 2.5 2.0 1.5 1.0 0.5 0.0
f1 (ppm)

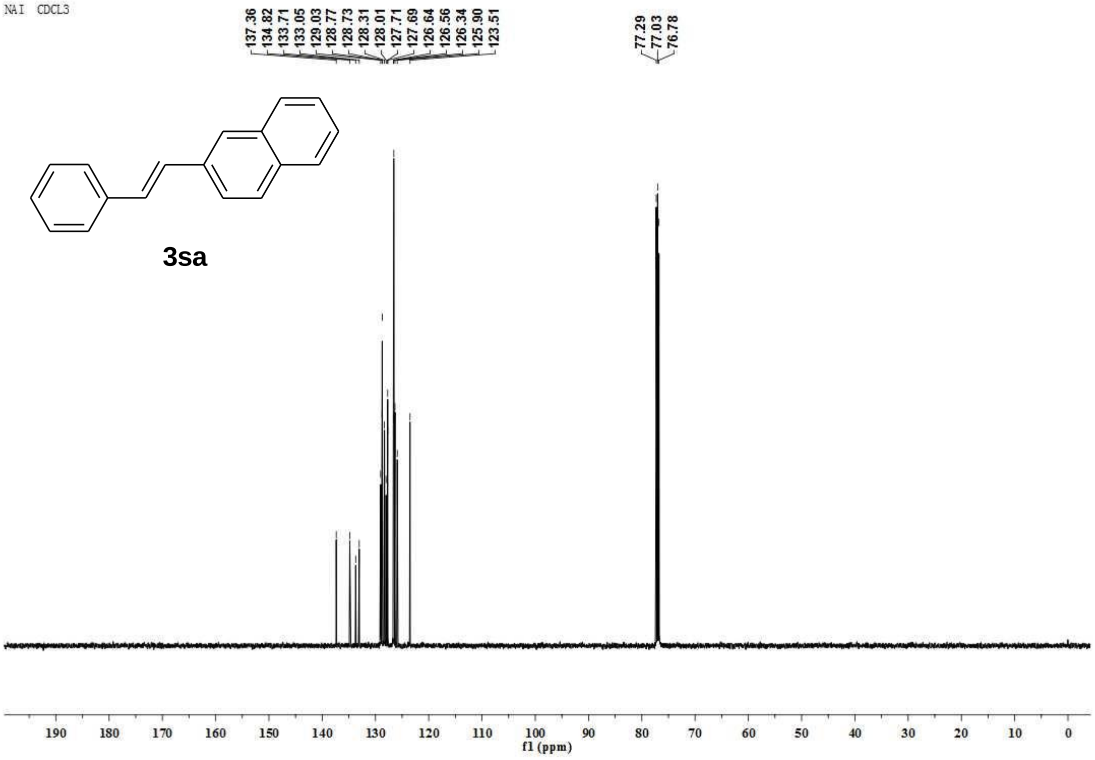
NAI CDCL3
137.36
134.82
133.71
133.05
129.03
128.77
128.73
128.31
128.01
127.71
127.69
126.64
126.56
126.34
125.90
123.51
77.29
77.03
76.78
3sa
190 180 170 160 150 140 130 120 110 100 90 80 70 60 50 40 30 20 10 0
f1 (ppm)

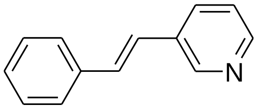

3ta

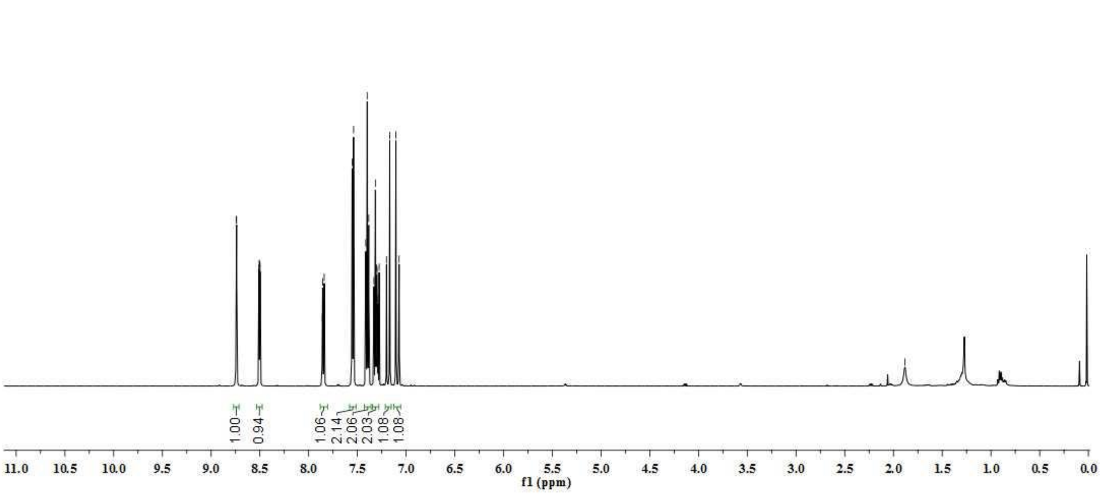
BIDING CDCL3
8.7432
8.7389
8.5100
8.5070
8.5005
8.4974
7.8598
7.8561
7.8523
7.8439
7.8402
7.8364
7.5536
7.5389
7.4135
7.4101
7.3988
7.3831
7.3321
7.3298
7.3275
7.3185
7.3146
7.3032
7.3002
7.2978
7.2879
7.2764
7.2007
7.1679
7.1044
7.0716
1.8828
1.00
0.94
1.06
2.14
2.06
2.03
1.08
1.08
f1 (ppm)

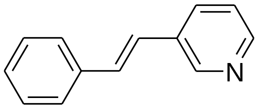

3ta

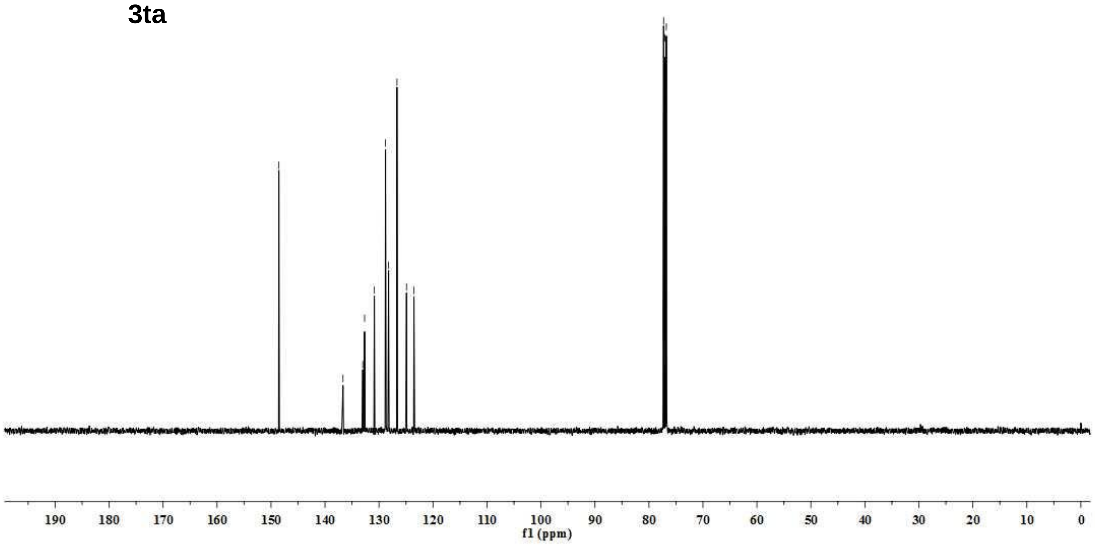
BIDING CDCL3
148.55
136.67
133.02
132.67
130.86
128.80
128.23
126.67
124.91
123.53
77.28
77.03
76.78
f1 (ppm)

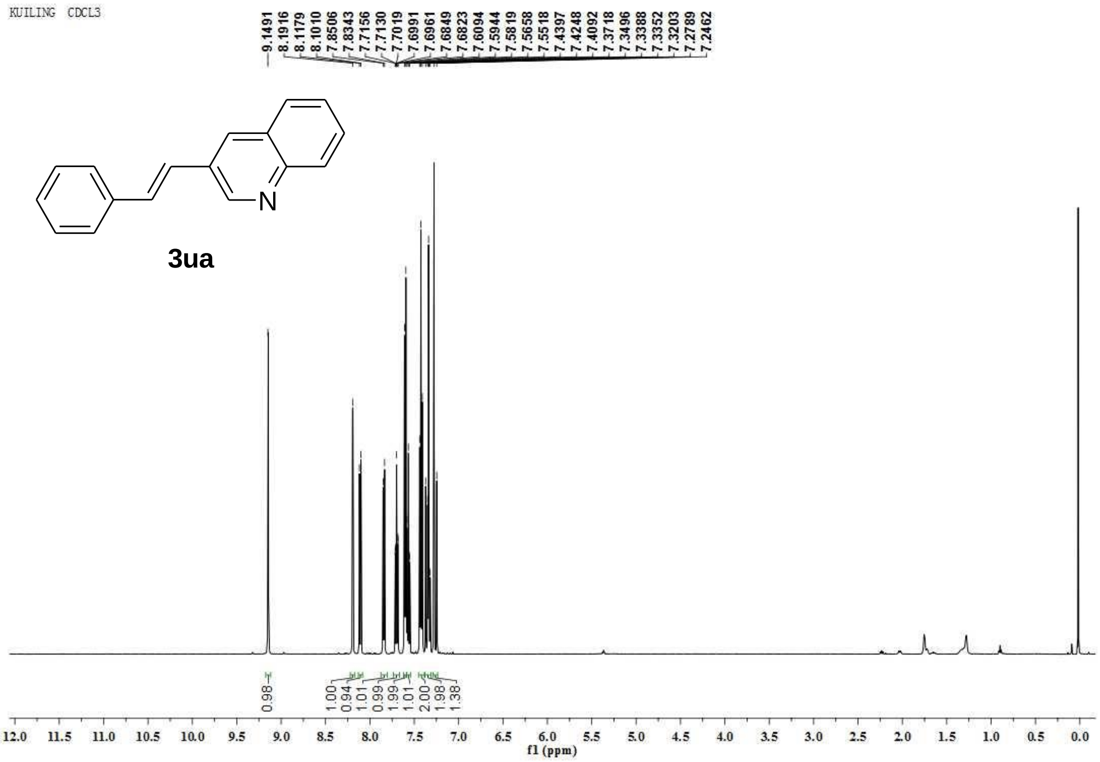
KUILING CDCL3
9.1491
8.1916
8.1179
8.1010
7.8506
7.8343
7.7156
7.7130
7.7019
7.6991
7.6961
7.6849
7.6823
7.6094
7.5944
7.5819
7.5658
7.5518
7.4397
7.4248
7.4092
7.3718
7.3496
7.3388
7.3352
7.3203
7.2789
7.2462
N
3ua
0.98
1.00
0.94
1.01
0.99
1.99
1.01
2.00
1.98
1.38
f1 (ppm)

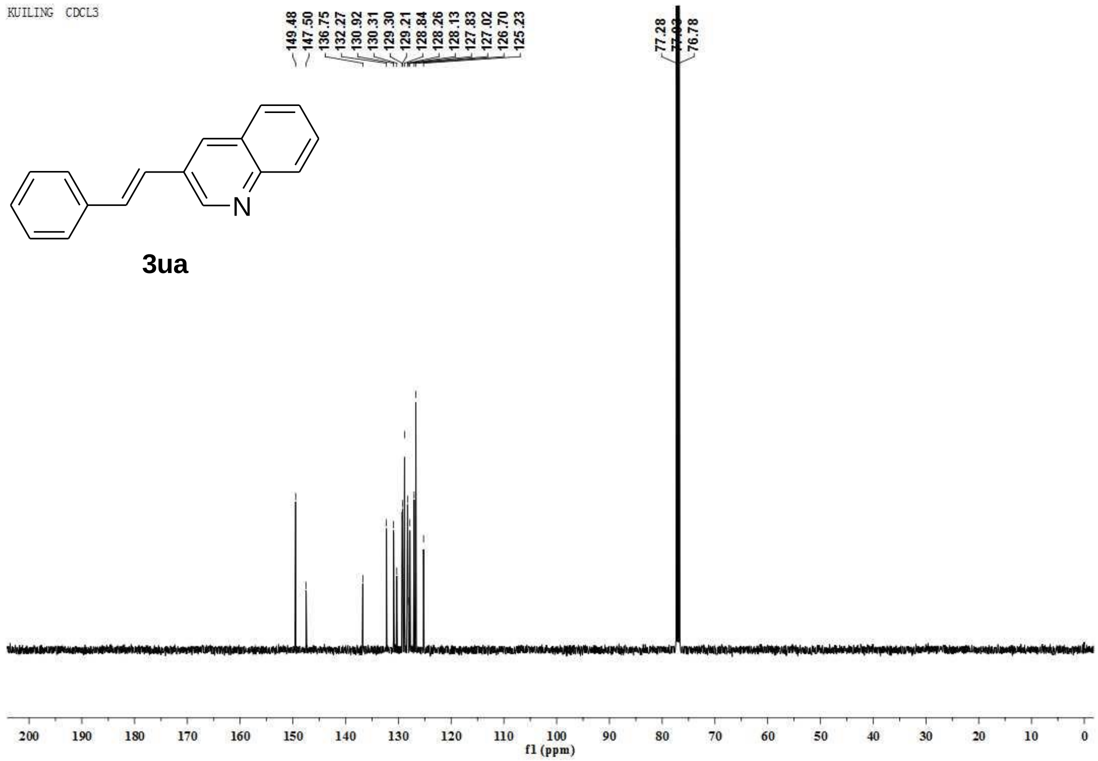
KUILING CDCL3
149.48
147.50
136.75
132.27
130.92
130.31
129.30
129.21
128.84
128.26
128.13
127.83
127.02
126.70
125.23
77.28
77.03
76.78
N
3ua
f1 (ppm)

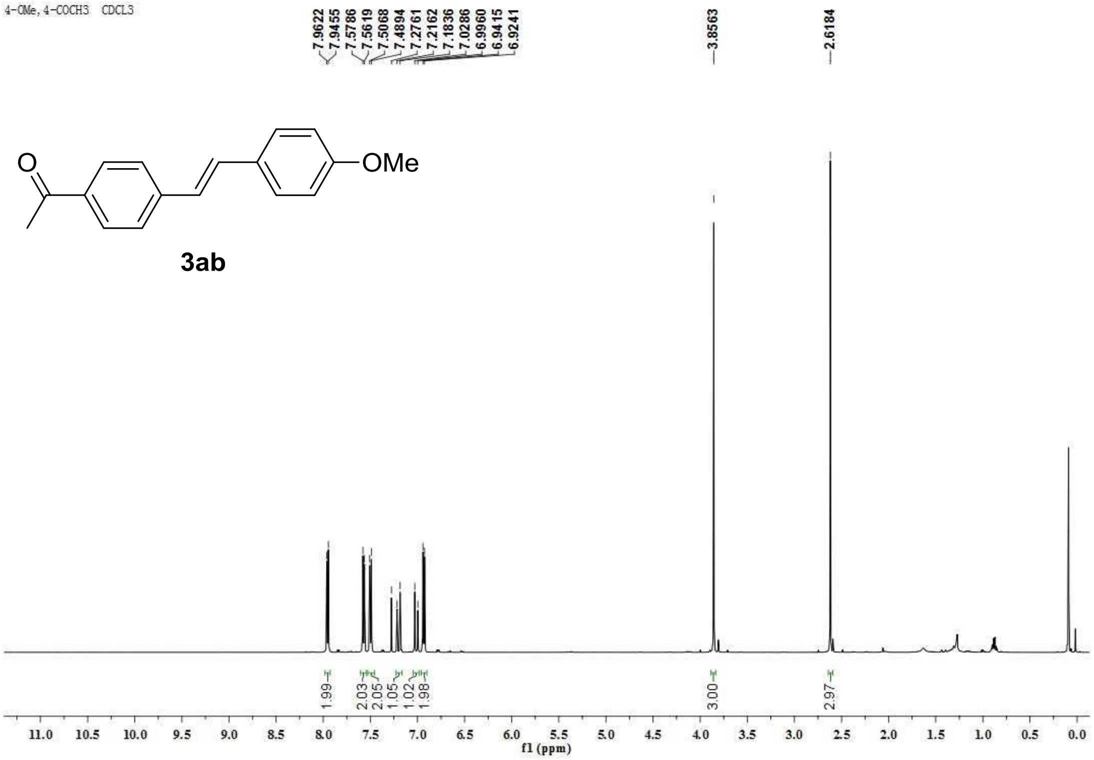
4-OMe,4-COCH3 CDCL3
7.9622
7.9455
7.5786
7.5619
7.5068
7.4894
7.2761
7.2162
7.1836
7.0286
6.9960
6.9415
6.9241
3.8563
2.6184
OMe
O
3ab
1.99
2.03
2.05
1.05
1.02
1.98
3.00
2.97
f1 (ppm)

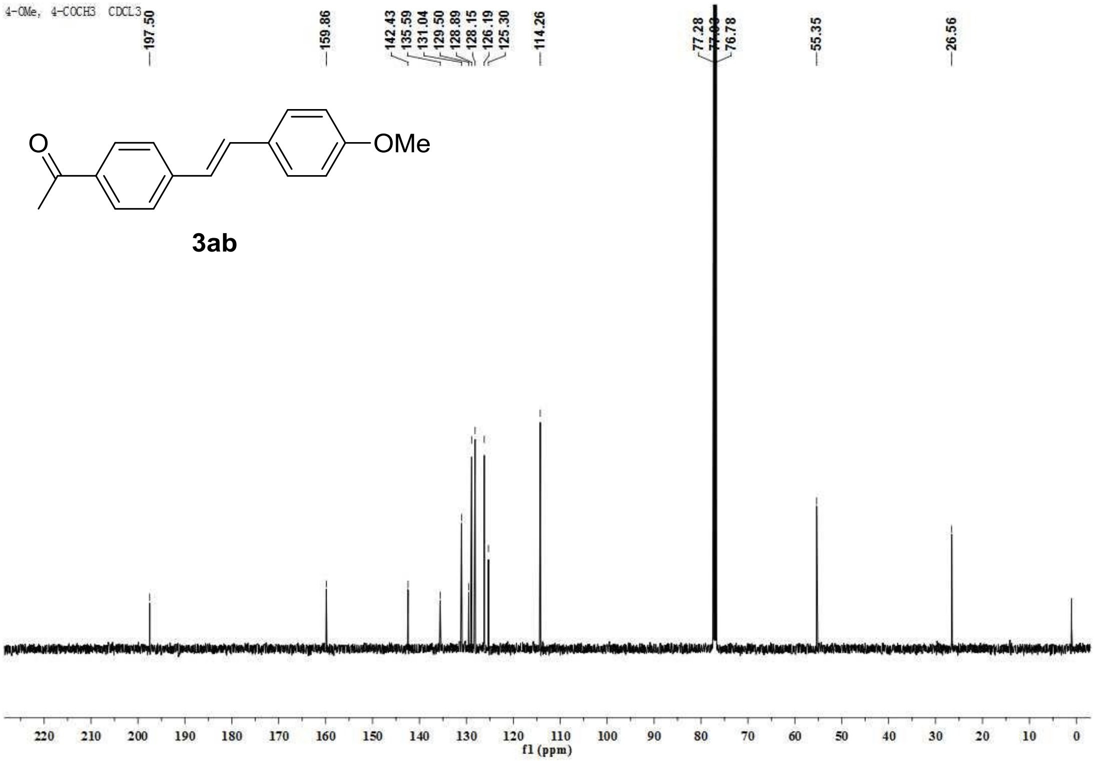
4-OMe, 4-COCH3 CDCL3
197.50
159.86
142.43
135.59
131.04
129.50
128.89
128.15
126.19
125.30
114.26
77.28
76.78
55.35
26.56
OMe
O
3ab
f1 (ppm)

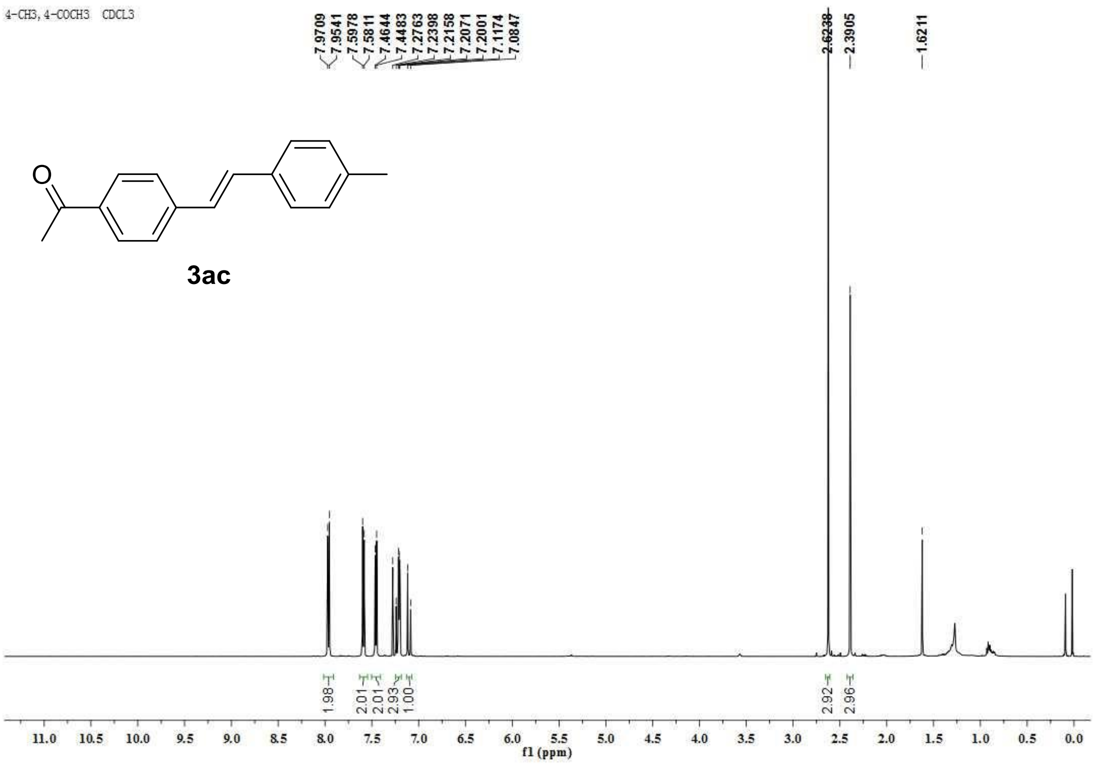
4-CH3, 4-COCH3 CDCL3
7.9709
7.9541
7.5978
7.5811
7.4644
7.4483
7.2763
7.2398
7.2158
7.2071
7.2001
7.1174
7.0847
2.6238
2.3905
1.6211
O
3ac
1.98
2.01
2.01
2.93
1.00
2.92
2.96
f1 (ppm)

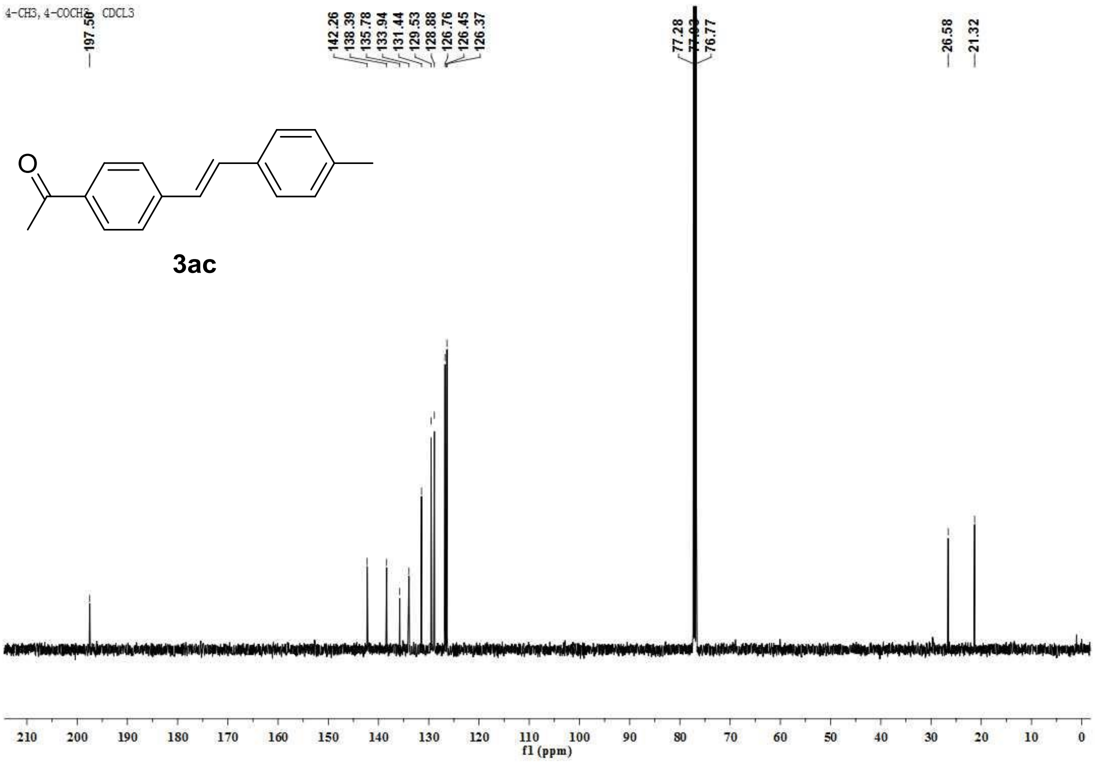
4-CH3, 4-COCH3 CDCL3
197.50
142.26
138.39
135.78
133.94
131.44
129.53
128.88
126.76
126.45
126.37
77.28
76.77
26.58
21.32
O
3ac
f1 (ppm)

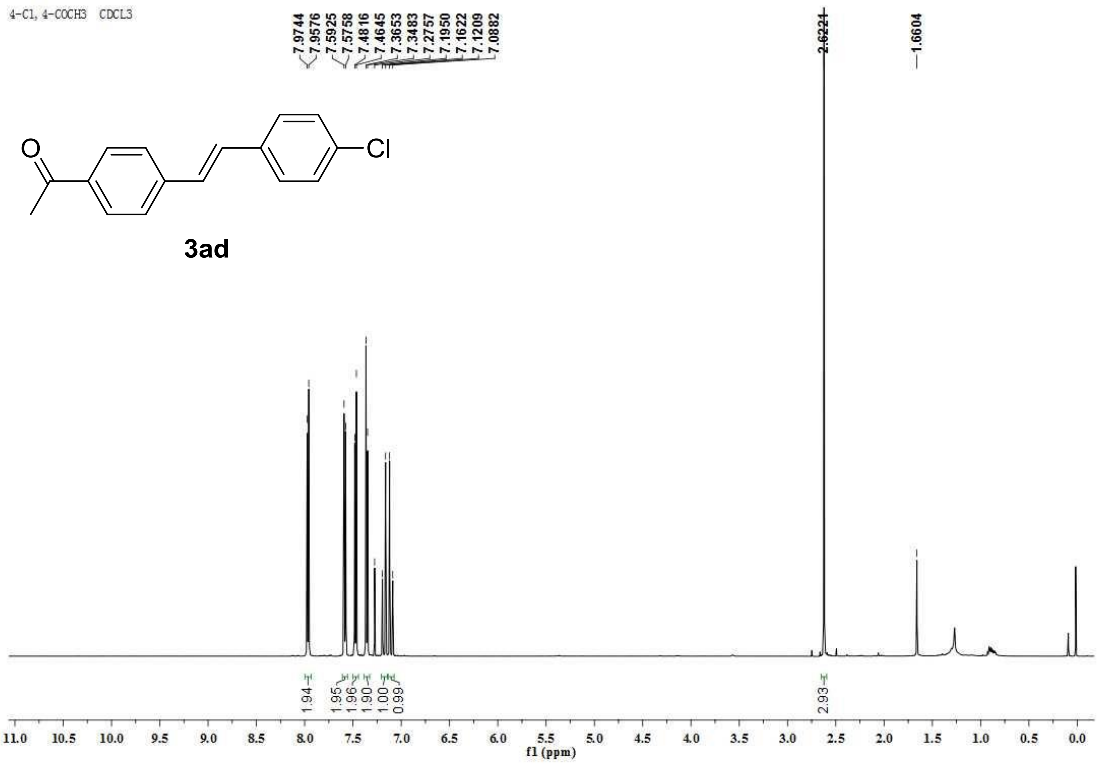
4-Cl, 4-COCH3 CDCL3
7.9744
7.9576
7.5925
7.5758
7.4816
7.4645
7.3653
7.3483
7.2757
7.1950
7.1622
7.1209
7.0882
2.6221
1.6604
Cl
O
3ad
1.94
1.95
1.96
1.90
1.00
0.99
2.93
f1 (ppm)

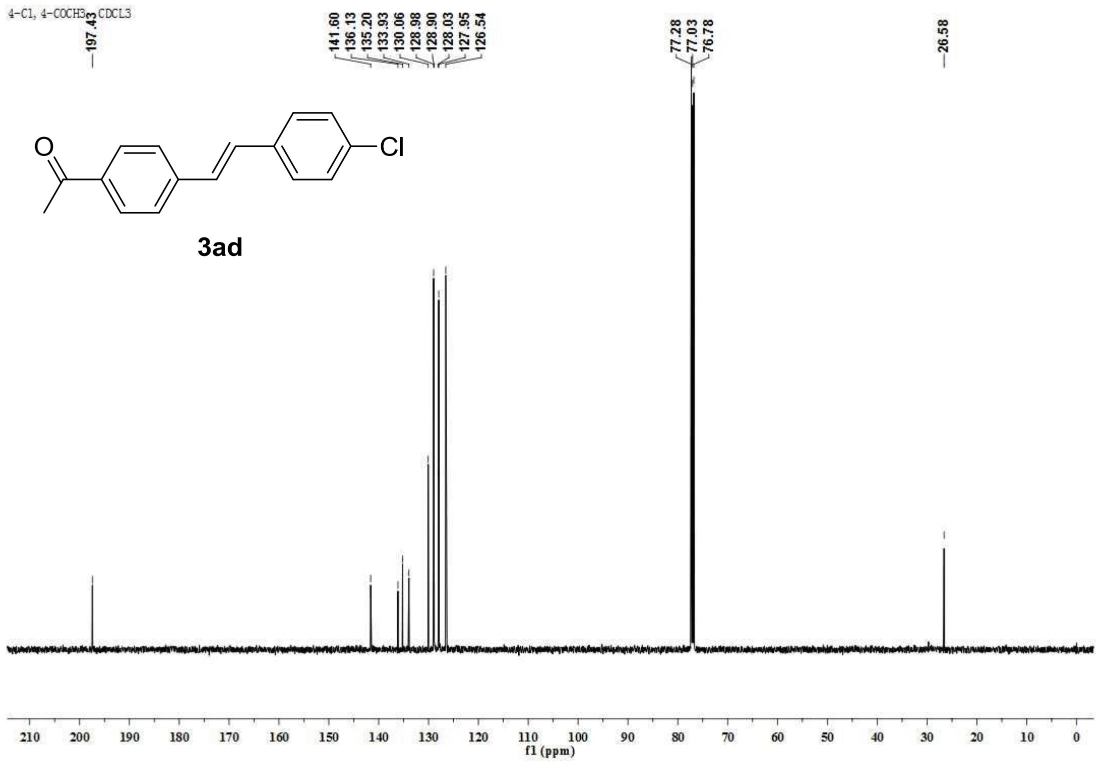
4-Cl, 4-COCH3 CDCL3
197.43
141.60
136.13
135.20
133.93
130.06
128.98
128.90
128.03
127.95
126.54
77.28
77.03
76.78
26.58
Cl
O
3ad
f1 (ppm)

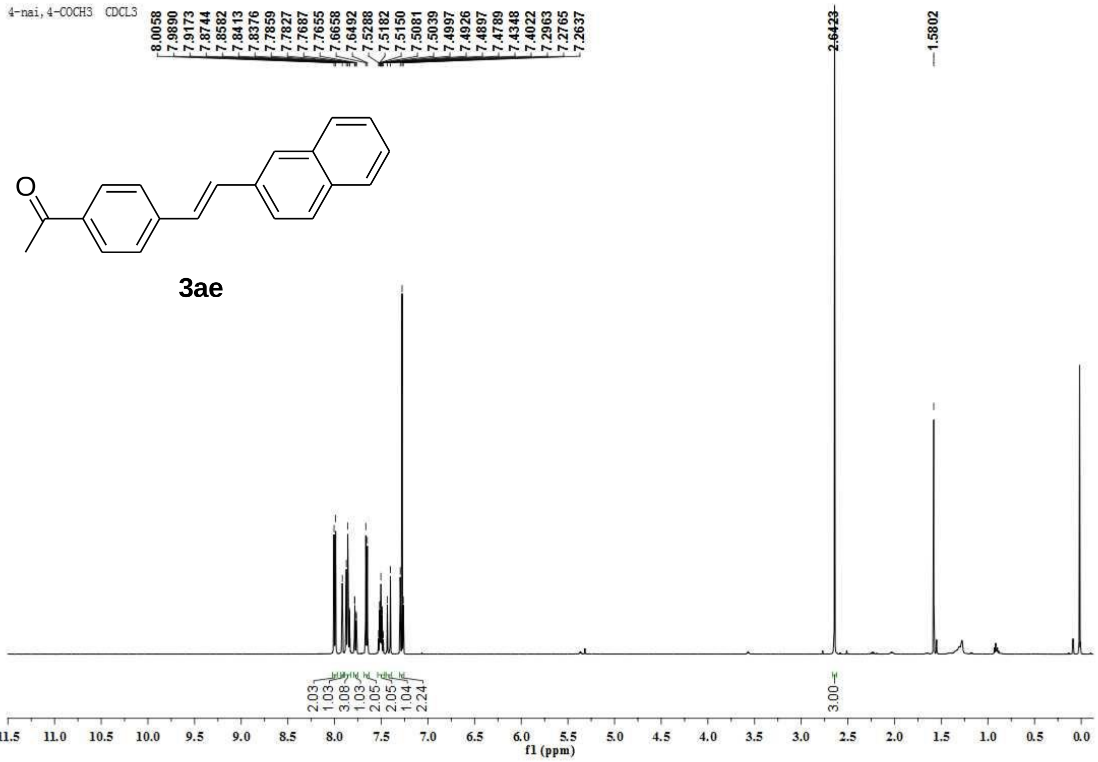
4-nai,4-COCH3 CDCL3
8.0058
7.9890
7.9173
7.8744
7.8582
7.8413
7.8376
7.7859
7.7827
7.7687
7.7655
7.6658
7.6492
7.5288
7.5182
7.5150
7.5081
7.5039
7.4997
7.4926
7.4897
7.4789
7.4348
7.4022
7.2963
7.2765
7.2637
2.6423
1.5802
2.03
1.03
3.08
1.03
2.05
2.05
1.04
2.24
3.00
f1 (ppm)

3ae

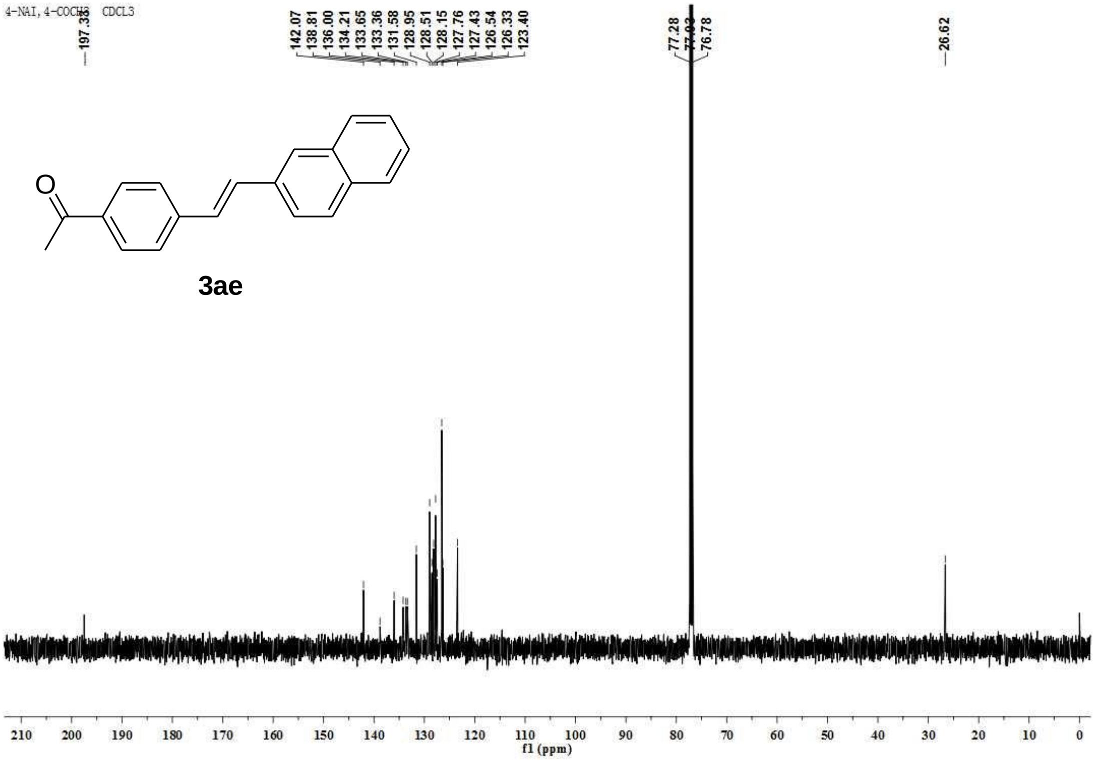
4-NAI,4-COCH3 CDCL3
197.33
142.07
138.81
136.00
134.21
133.65
133.36
131.58
128.95
128.51
128.15
127.76
127.43
126.54
126.33
123.40
77.28
77.02
76.78
26.62
f1 (ppm)

3ae

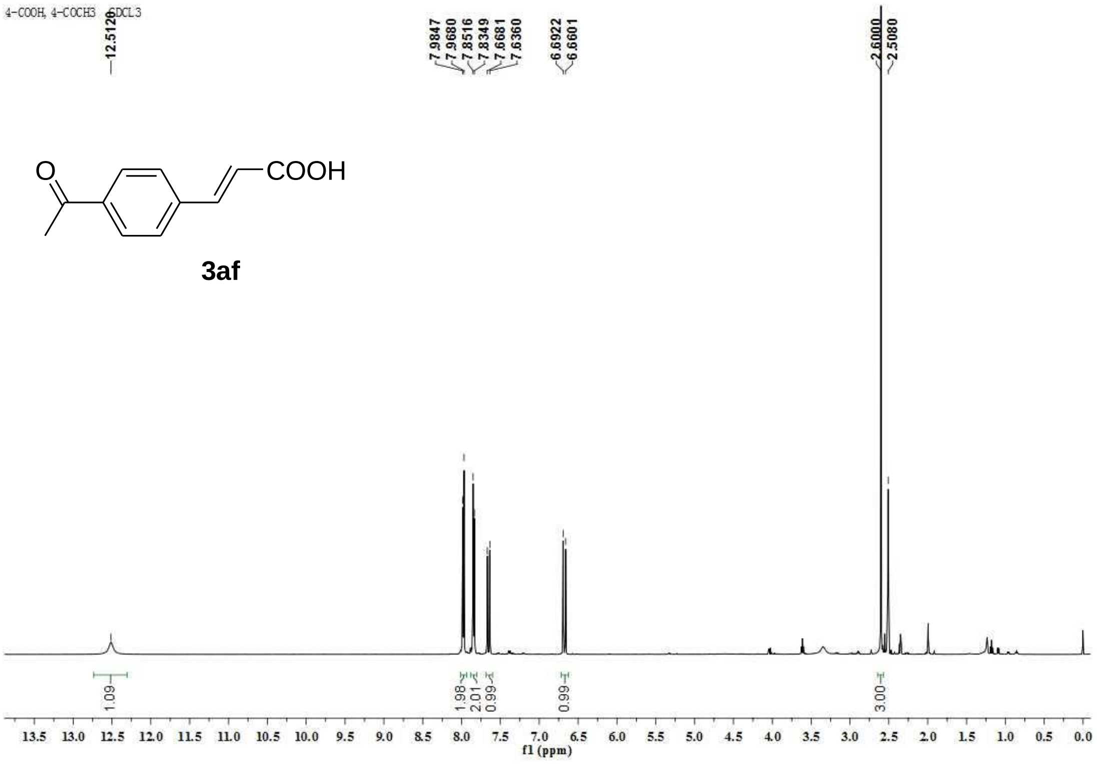
4-COOH, 4-COCH3 CDCL3
12.5129
7.9847
7.9680
7.8516
7.8349
7.6681
7.6360
6.6922
6.6601
2.6000
2.5080
COOH
O
3af
1.09
1.98
2.01
0.99
0.99
3.00
13.5 13.0 12.5 12.0 11.5 11.0 10.5 10.0 9.5 9.0 8.5 8.0 7.5 7.0 6.5 6.0 5.5 5.0 4.5 4.0 3.5 3.0 2.5 2.0 1.5 1.0 0.5 0.0
f1 (ppm)

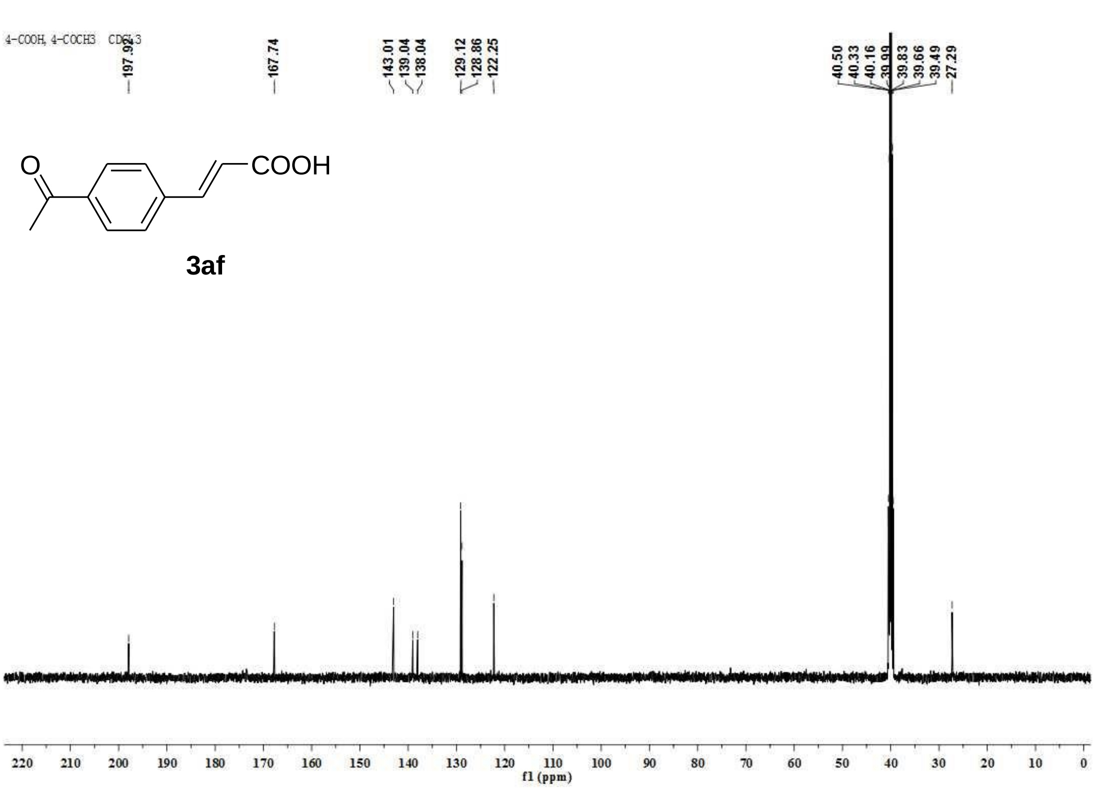
4-COOH, 4-COCH3 CDCL3
197.92
167.74
143.01
139.04
138.04
129.12
128.86
122.25
40.50
40.33
40.16
39.99
39.83
39.66
39.49
27.29
COOH
O
3af
220 210 200 190 180 170 160 150 140 130 120 110 100 90 80 70 60 50 40 30 20 10 0
f1 (ppm)

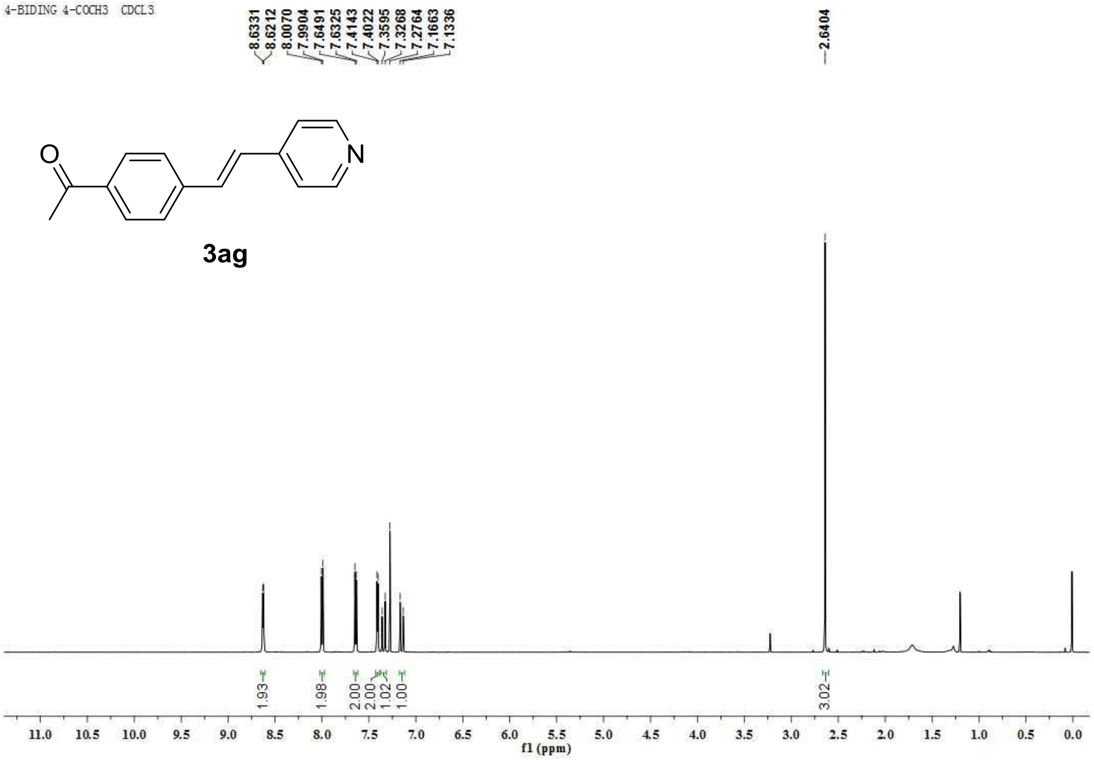
4-BIDING 4-COCH3 CDCL3
8.6331
8.6212
8.0070
7.9904
7.6491
7.6325
7.4143
7.4022
7.3595
7.3268
7.2764
7.1663
7.1336
2.6404
3ag
1.93
1.98
2.00
2.00
1.02
1.00
3.02
11.0 10.5 10.0 9.5 9.0 8.5 8.0 7.5 7.0 6.5 6.0 5.5 5.0 4.5 4.0 3.5 3.0 2.5 2.0 1.5 1.0 0.5 0.0
f1 (ppm)

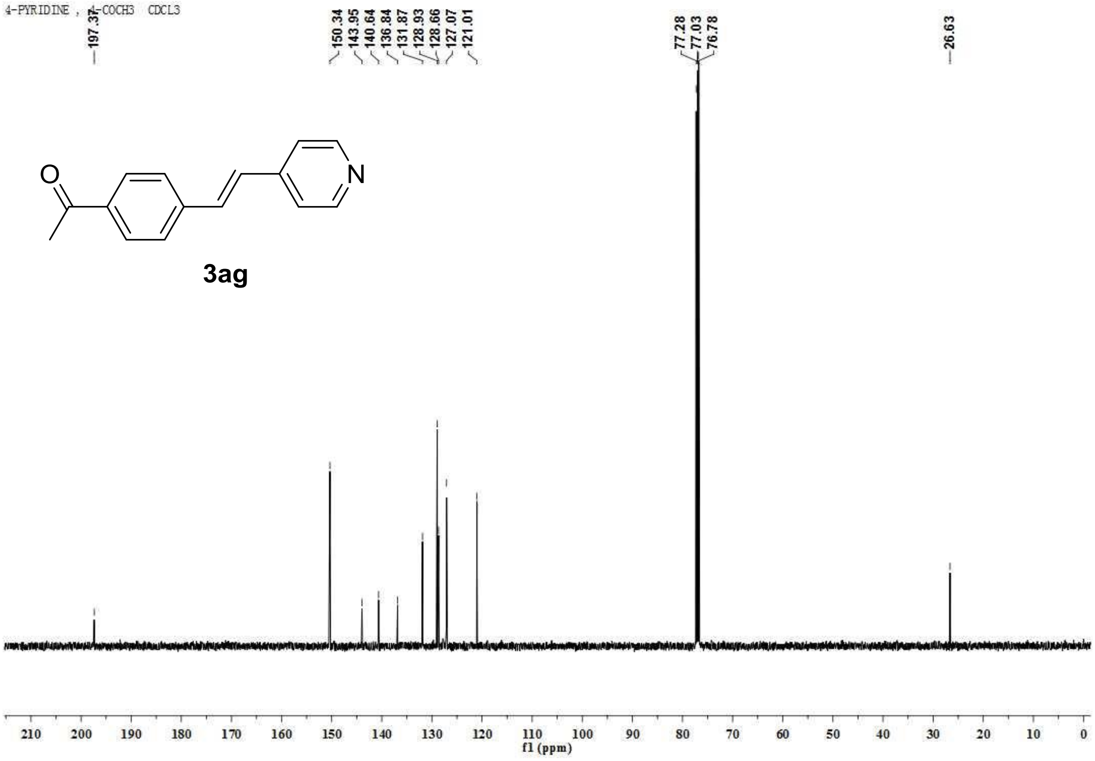
4-PYRIDINE , 4-COCH3 CDCL3
197.37
150.34
143.95
140.64
136.84
131.87
128.93
128.66
127.07
121.01
77.28
77.03
76.78
26.63
3ag
210 200 190 180 170 160 150 140 130 120 110 100 90 80 70 60 50 40 30 20 10 0
f1 (ppm)

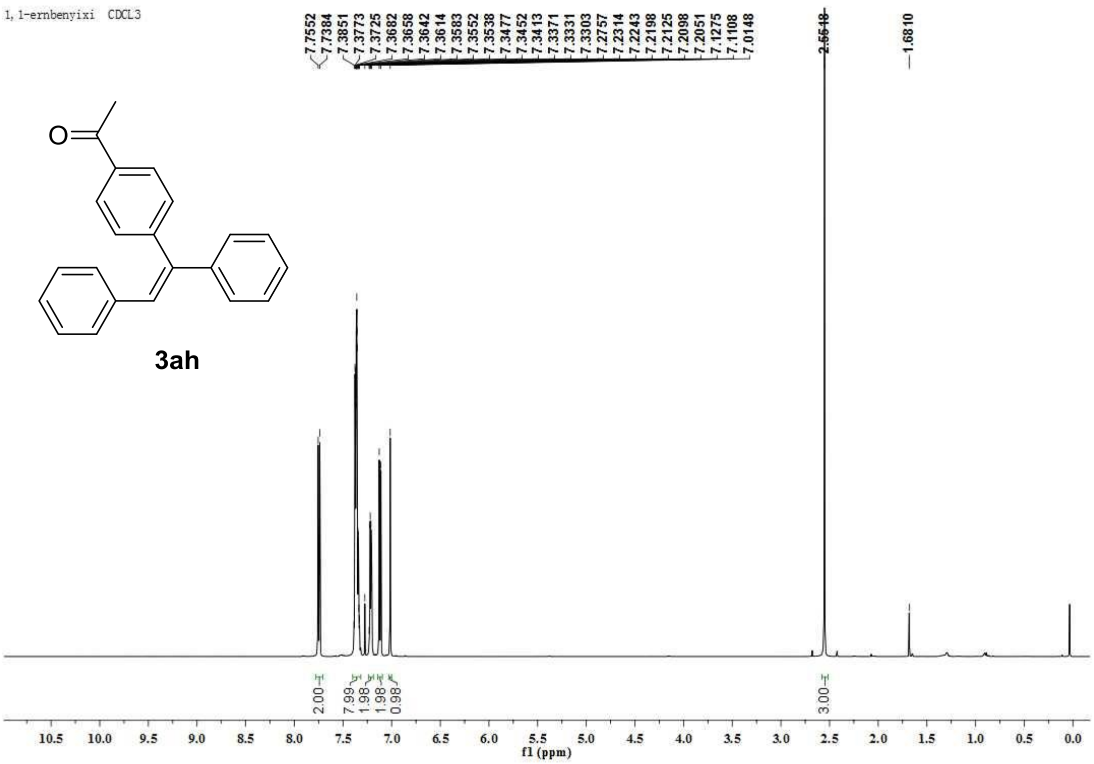
1, 1-ernbenyixi CDCL3
7.7552
7.7384
7.3851
7.3773
7.3725
7.3682
7.3658
7.3642
7.3614
7.3583
7.3552
7.3538
7.3477
7.3452
7.3413
7.3371
7.3331
7.3303
7.2757
7.2314
7.2243
7.2198
7.2125
7.2098
7.2051
7.1275
7.1108
7.0148
2.5518
1.6810
O
3ah
2.00
7.99
1.98
1.98
0.98
3.00
f1 (ppm)

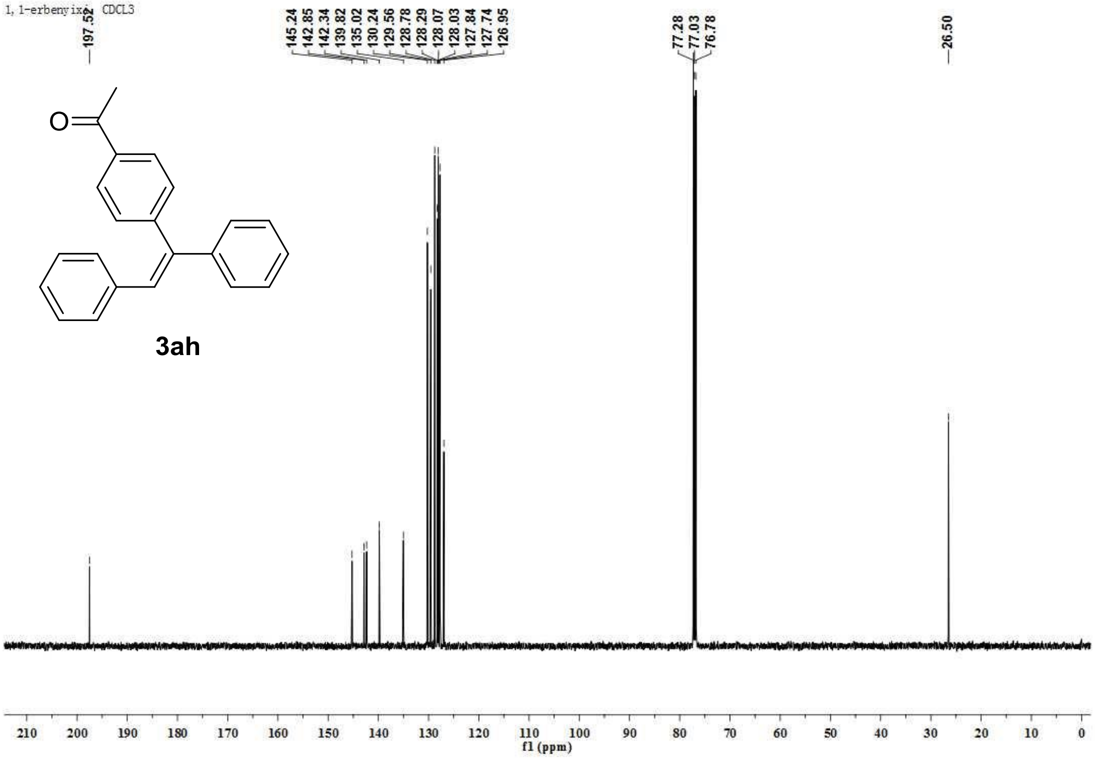
1, 1-erbenyixi CDCL3
197.52
145.24
142.85
142.34
139.82
135.02
130.24
129.56
128.78
128.29
128.07
128.03
127.84
127.74
126.95
77.28
77.03
76.78
26.50
O
3ah
f1 (ppm)
